# Copper(i)-catalyzed click chemistry in deep eutectic solvent for the syntheses of β-d-glucopyranosyltriazoles†

**DOI:** 10.1039/d3ra01844j

**Published:** 2023-04-03

**Authors:** Subrat Sethi, Narayan Ch. Jana, Surajit Panda, Suraj Kumar Maharana, Bidraha Bagh

**Affiliations:** a School of Chemical Sciences, National Institute of Science Education and Research (NISER), An OCC of Homi Bhabha National Institute Jatni, Khurda Bhubaneswar Odisha PIN 752050 India bidraha@niser.ac.in

## Abstract

In the last two decades, click chemistry has progressed as a powerful tool in joining two different molecular units to generate fascinating structures with a widespread application in various branch of sciences. copper(i)-catalyzed azide–alkyne cycloaddition (CuAAC) reaction, also known as click chemistry, has been extensively utilized as a versatile strategy for the rapid and selective formation of 1,4-disubstituted 1,2,3-triazoles. The successful use of CuAAC reaction for the preparation of biologically active triazole-attached carbohydrate-containing molecular architectures is an emerging area of glycoscience. In this regard, a well-defined copper(i)–iodide complex (1) with a tridentate NNO ligand (L_1_) was synthesized and effectively utilized as an active catalyst. Instead of using potentially hazardous reaction media such as DCM or toluene, the use of deep eutectic solvent (DES), an emerging class of green solvent, is advantageous for the syntheses of triazole-glycohybrids. The present work shows, for the first time, the successful use of DES as a reaction medium to click various glycosides and terminal alkynes in the presence of sodium azide. Various 1,4-disubstituted 1,2,3-glucopyranosyltriazoles were synthesized and the pure products were isolated by using a very simple work-up process (filtration). The reaction media was recovered and recycled in five consecutive runs. The presented catalytic protocol generated very minimum waste as reflected by a low *E*-factor (2.21–3.12). Finally, the optimized reaction conditions were evaluated with the CHEM21 green metrics toolkit.

## Abstract 2

A well-defined copper(i)–iodide complex was effectively utilized as an active catalyst for azide–alkyne cycloaddition to synthesize various 1,4-disubstituted 1,2,3-glucopyranosyltriazoles in deep eutectic solvents as a reusable reaction media.

## Graphical abstract

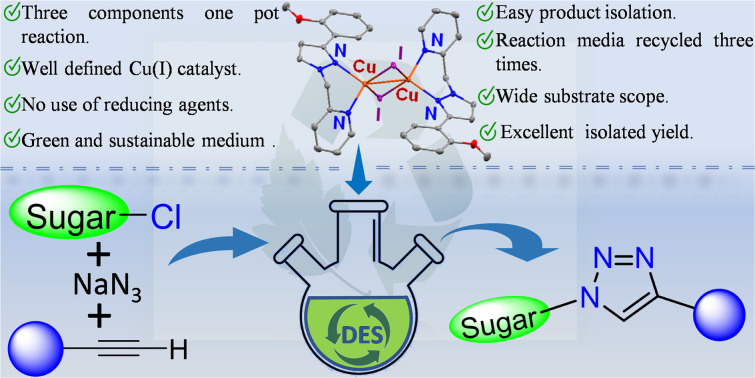

## Introduction

In 2002, Karl B. Sharpless^1^ and Morten P. Meldal^2^ independently discovered copper(i)-catalyzed 1,3-dipolar cycloaddition reaction between organic azides and terminal alkynes, commonly known as CuAAC or the “click reaction”. Later the reaction became popular to the scientific community because of its flexible and adaptable strategies for the easy syntheses of 1,4-disubstituted 1,2,3-triazoles which have widespread application in medicinal,^3–5^ pharmaceutical,^6,7^ biological,^8–12^ and material sciences,^13–17^ drug discoveries^18–21^ and catalysis.^22–26^ Indeed, the 2022 Nobel prize in Chemistry went to both Karl B. Sharpless and Morten P. Meldal along with Carolyn R. Bertozzi, for their seminal work on click and bioorthogonal chemistry. Unlike Huisgen's uncatalyzed thermal version,^27^ the copper(i) catalyzed version is absolutely regioselective and regiospecific. Not only that, the catalytic protocol is easy to perform under very mild conditions and provides high to excellent yields with a wide variety of substrate scope. Therefore, there is no doubt the CuAAC reaction is a novel discovery in the context of “click chemistry’’ as coined by Sharpless in 1999.^28^

The impact of click chemistry has already been spread out in diverse fields of science.^29–45^ Along with the successful development of CuAAC reactions in various fields of chemistry, its application in carbohydrate chemistry has been parallelly explored.^46–58^ In this regard, Tiwari and co-workers elegantly illustrated the application of click chemistry in glycoscience.^46,47^ Carbohydrates are very common class of biomolecules and an integral part of living cells. They are significantly important in the construction of structural building blocks of genetic materials and serve as necessary energy source.^59^ They play vital roles in different intracellular and intercellular activities in form of signal-transmeter, cell surface receptors and bacterial adhesives.^60,61^ Carbohydrate moieties are also boon for human use because of their hydrophilicity, minimum toxicity, biocompatibility and bioavailability.^59–63^ Due to all these superior properties, synthetic organic chemists showed an enormous interest in designing carbohydrate scaffolds for easy access to the biologically active molecules.^63^ In this context, an intriguing approach to click carbohydrate-based alkyne and azide functionality is the CuAAC reaction. CuAAC reaction allow facile access to emerging class of triazole-appended carbohydrate building blocks such as glycoconjugates,^46,47^ glycopolymers,^64^ glycohybrids,^65^ glycopeptides,^66^ glycoproteins,^67^ glycolipids,^68^ glycoclusters,^48^ and glycodendrimers.^69^ These glycoproducts have widespread applications including medicinal science, material science, gelation, chelation, sensing, glycosylation, phermacology and catalysis.^46–58^ However, sustainable synthetic approach of this modular and bio-orthogonal click-carbohydrate chemistry is less explored.

**Scheme 1:**
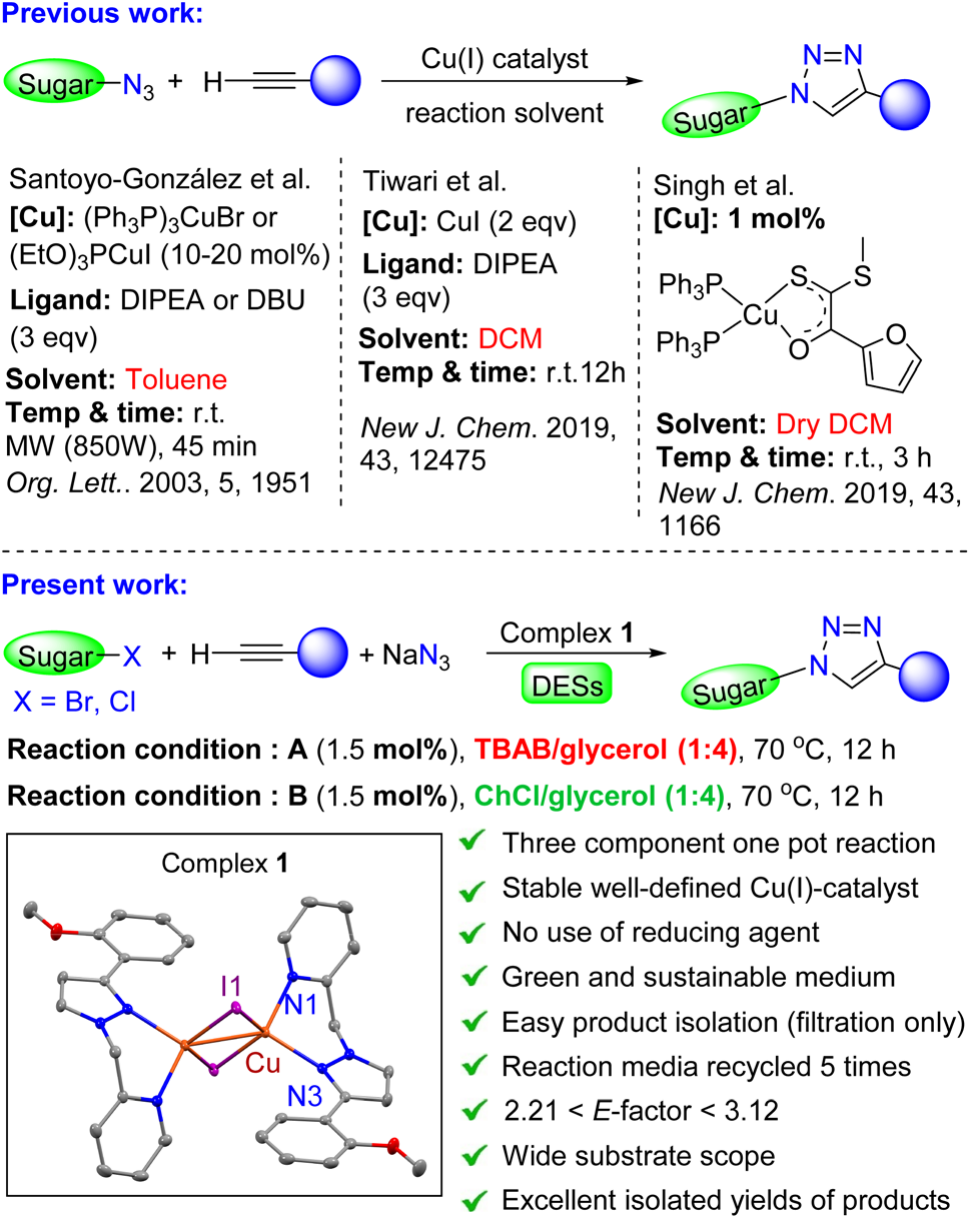
CuAAC reaction in synthesis of triazolyl glycoconjugates and sustainable features of the present work.

Many CuAAC reactions have been performed in conventional green solvents such as water, alcohols and their mixtures.^70–73^ However, various groups carried out CuAAC reactions in harmful organic solvents such acetonitrile, DCM, toluene and THF.^74–77^ There is a recent trend to find alternative (non-hazardous) solvents. Vaccaro *et al.* used a mixture of water and biomass-derived furfuryl alcohol^78^ and a mixture of water and Polarclean^79^ as green media. Various groups used ionic liquids^80^ and deep eutectic solvents (DESs)^81–83^ as alternative reaction media. Very recently, we reported CuAAC reaction in presence of copper(i) coordination polymer as effective catalyst in DES as sustainable solvent.^84^ The selection of sustainable solvents in “click chemistry” for the syntheses of glucopyranosyltriazoles is equally important. The catalytic protocols for the syntheses of 1,4-disubstituted 1,2,3-triazoles linked with glycoconjugates mostly used environmentally hazardous solvents like dichloromethane (DCM), toluene, dimethylformamide (DMF) and acetonitrile.^46,47^ In 2003, Santoyo-González have prepared multivalent neoglycoconjugates by using the organic-soluble copper complexes (Ph_3_P)_3_CuBr and (EtO)_3_PCuI as catalysts in toluene under microwave irradiation (Scheme 1).^85^ Tiwari and co-workers reported elegant syntheses of various triazolyl glycoconjugates embedded with ethisterone,^86^*o*-benzylquercertin,^87^ noscapine,^88^ bis-triazolyl,^89^ vanillin,^90^ 1,3,4-oxadiazole,^91^ noscapine^92,93^ and glycodendrimers^94^ in the presence of CuI and DIPEA in DCM (Scheme 1). However, a large amount of copper and ligands was used. Very recently, Singh and co-workers also utilized DCM as reaction medium for the efficient syntheses of a variety of glycoconjugate triazoles in presence of mono or dinuclear Cu(i) complexes.^95,96^ We paid serious attention to perform CuAAC reactions for the syntheses of glucopyranosyltriazoles, in which catalytic protocol does not require extra purification steps and can also be performed in environmentally benign solvents. In this context, Dondoni and co-workers validated the CuAAC reaction in 10 different ionic liquids which opens up a new perspective in glyco-chemistry using this prototype click method.^97^ However, a large majority of ionic liquids are expensive, very complex to prepare, highly toxic and non-biodegradable. In contrast, various synthetic research groups have considered DES (particularly, metal-free type-III DES) as an emerging class of green media with distinct properties which can balance the major disadvantages of ionic liquids.^98–103^ However in a very recent review, Marrucho and Guazzelli *et al.* expressed serious concerns for the general notion of considering DES as a much better alternative to ionic liquids.^104^ Although, they concluded DESs (with wisely selected starting components) as a promising environmentally benign solvent to replace traditional volatile organic solvents. To the best of our literature search, the use of DES in click inspired syntheses of glycoconjugates have not been investigated so far. Hence, we studied the potential applicability of DES as sustainable reaction medium for the synthesis of important glycoconjugate triazoles for the first time *via* CuAAC reaction (Scheme 1). Herein, we report readily accessible and well-defined copper(i)-iodide complex with NNO ligand framework as efficient catalyst for the syntheses of various glucopyranosyltriazoles in DESs (Scheme 1).

## Results and discussion

**Scheme 2:**
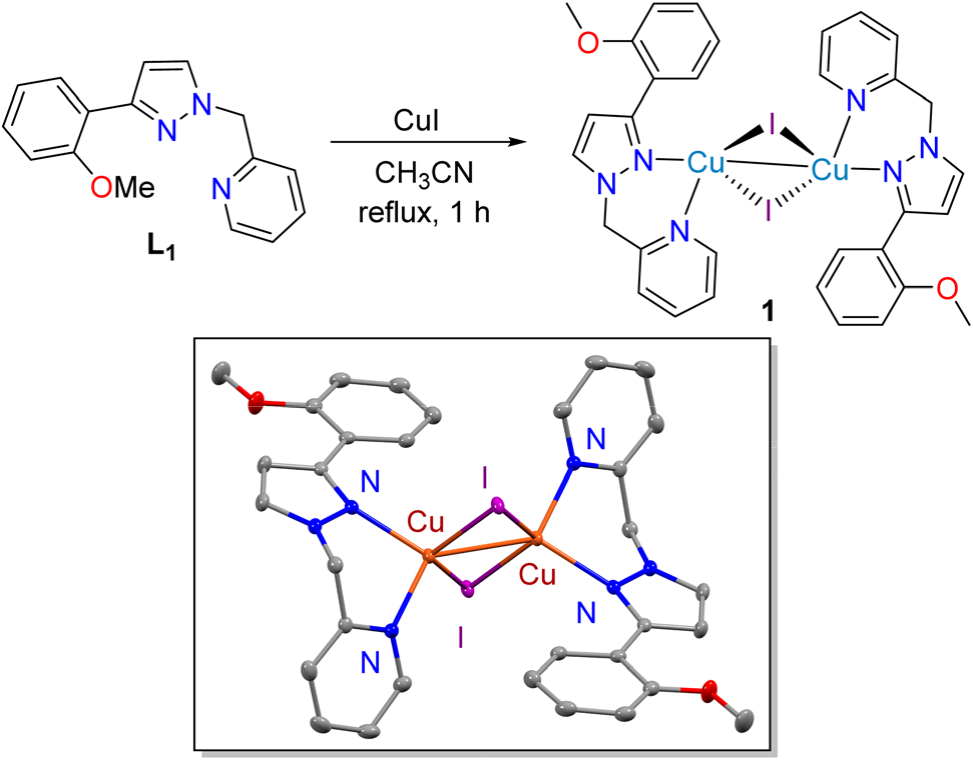
Synthesis of copper(i) iodide complex 1 (the molecular structure of complex 1 showing 30% ellipsoids and hydrogen atoms are omitted for clarity).

In the past two decades, various copper(i) complexes have been extensively used as catalysts in CuAAC reactions.^29–45^ The active copper(i) species required for this chemical transformation can also be generated *in situ* by the reaction of copper(ii) salt with a reducing agent such as sodium ascorbate. However, problem arises with the stability of the copper(i) complexes during the course of reaction and poor solubility of copper-(i) salts in common organic solvents. Therefore, considerable attention has been paid in recent years to synthesize stable as well active copper(i) complexes with appropriate ligand systems.^105^ In this regard, majority of the developed copper(i) systems are those with phosphines and *N*-heterocycle carbenes (NHCs).^106,107^ Phosphines and NHCs are the ligands with strong donor ability and resulting copper(i) complexes showed good catalytic activity. Very recently, phosphine-based copper(i) oxodithioester, dixanthate and xanthate complexes are used for synthesis of triazolyl glyconjugates under homogeneous conditions.^95,96^ Complexes with N, O and S donor ligand systems also used for this CuAAC reaction.^108–110^ Recently, our group reported an air-stable and well-defined copper(i)-chloride coordination polymer with NNS ligand framework for sustainable synthesis of various triazoles in green solvents.^84^ However, coordination of that NNS ligand with CuI resulted in insoluble solid and we could not reveal its identity. Similar tridentate NNO ligand system^111,112^ might also be effective to develop copper(i) catalyst with high efficiency. Therefore, we have selected a NNO ligand L_1_ and facile coordination of ligand L_1_ with CuI resulted in the formation of a dinuclear copper(i) complex 1 (Scheme 2). Complex 1 was characterized by ^1^H and ^13^C NMR spectroscopy, IR spectroscopy, mass analysis and elemental analysis. In the ^1^H NMR spectrum of complex 1, the methoxy methyl and methylene protons were appeared as singlet at 3.76 and 5.55 ppm, respectively. Aromatic protons were observed in the expected downfield region (6.70–8.52 ppm). In the IR spectrum of complex 1, mixed stretching vibration of C=N and C=C bonds of the pyrazole ring was slightly shifted to 1515 cm^−1^ in comparison to the free ligand L_1_ (1505 cm^−1^). Symmetric and asymmetric vibrations due to C(sp^3^)-H bonds stretching was observed in the range of 3130 to 2740 cm^−1^. The vibrational stretching bands in the range of 1400 to 1640 cm^−1^ were assigned for phenyl (C=C) and pyridyl (C=N) functionalities. Molecular ion peak was observed at 328.0517 in the mass spectrum of complex 1.

We have further used single crystal X-ray diffraction analysis to know the geometrical identity of complex 1 (Scheme 2). Single crystal X-ray diffraction analysis revealed that complex 1 crystallized in monoclinic system with space group *P*2_1_/*c*. The asymmetric unit of 1 consist of half of the centrosymmetric dimer in which the copper centre is surrounded by two nitrogen atoms of the NNO ligand and one iodide. It is worth to mention that the hard oxygen donor ether moiety did not bind to the copper centre and was moved away from the metal through the rotation of the C–C bond. Although, this NNO ligand has been reported as a tridentate ligand, L1 behaves as a bidentate ligand with CuI. The coordination geometry of Cu(i) center in complex 1 can be best described as highly distorted tetrahedral due to the presence of large iodide donors.^109,110^ The Cu⋯Cu distance in 1 is 2.6101(6) Å, which is in the range of Cu⋯Cu single bond distance (ideal Cu⋯Cu covalent bond distance is 2.64 Å while the corresponding van der walls' distance is 2.80 Å).^110^ The Cu–N_pyridyl_, Cu–N_pyrazolyl_ and Cu–I bond lengths are 2.091(5), 2.118 (5) and 2.627(13) Å, respectively. All these bond distances are in the expected range and are consistent with similar Cu(i) complexes having tetrahedral geometry.^109,110^ It is worth to mention that binuclear copper complex 1 is highly stable in solid state for weeks; however, it slowly decomposes upon long exposure in air in solution state.

A substantial amount of research effort has been devoted in search of better catalysts for click reaction and this can be considered as a general trend in different fields of catalysis. In contrast, traditionally research community has paid minor attentions in selecting sustainable reaction media. The click reaction in glyco-chemistry have already been explored and the reaction is performed in various hazardous organic solvents such as toluene, DCM, acetonitrile, DMF and THF.^46–58,86–96^ Herein, for the first time, we studied the use of deep eutectic solvents (DESs) as reaction media for the syntheses of triazolyl glycoconjugates. In our present work, a series of DESs were prepared through the combination of various hydrogen-bond acceptor such as choline chloride (ChCl), tetra butyl ammonium bromide (TBAB), tetramethyl ammonium chloride (TMAC) and methyl triphenyl phosphonium bromide (MTPB) and various hydrogen bond donors, namely urea, thiourea, glycerol and ethylene glycol. A literature ratio^100,113^ of this hydrogen-bond acceptor and hydrogen bond donors was used to prepared total ten DESs and all were screened for the syntheses of triazolyl glycoconjugates *via* prototype click reaction catalyzed by Cu(i) iodide complex 1 (Table 1). It is worth to mention that we used type III DESs as a class of metal-free green solvents.

**Table 1:** Catalytic activity of complex 1 for CuAAC reaction in various DESs^a^

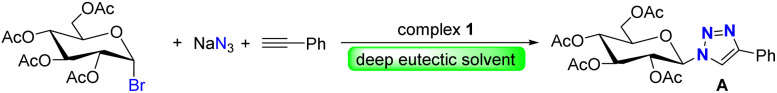					
Ent.	Cat. 1 (mol%)	Solvent mixture (ratio)	Temp. (°C)	Time (h)	Yield^b^ (%)
1	1.5	ChCl/glycerol (1 : 2)	r.t.	24	35
2	1.5	ChCl/glycerol (1 : 2)	70	24	97
3	1.5	ChCl/ethylene glycol (1 : 2)	70	24	83
4	1.5	ChCl/thiourea (1 : 2)	70	24	97
5	1.5	ChCl/urea (1 : 2)	70	24	70
6	1.5	TBAB/glycerol (1 : 4)	70	24	98
7	1.5	TBAB/ethylene glycol (1 : 4)	70	24	82
8	1.5	TMAC/glycerol (1 : 2)	70	24	56
9	1.5	TMAC/ethylene glycol (1 : 2)	70	24	60
10	1.5	MTPB/glycerol (1 : 3)	70	24	97
11	1.5	MTPB/ethylene glycol (1 : 3)	70	24	98
**12**	**1.5**	**ChCl/glycerol (1** : **2)**	**70**	**12**	**94**
13	1.5	ChCl/thiourea (1 : 2)	70	12	58
**14**	**1.5**	**TBAB/glycerol (1** : **4)**	**70**	**12**	**98**
15	1.5	MTPB/glycerol (1 : 3)	70	12	80
16	1.5	MTPB/ethylene glycol (1 : 3)	70	12	76
17	1.5	TBAB/glycerol (1 : 4)	70	10	87
18	1.5	TBAB/glycerol (1 : 4)	70	8	75
19	1	TBAB/glycerol (1 : 4)	70	12	76
20	1.5	TBAB/glycerol (1 : 4)	r.t.	12	31

a Reactions conducted in a vial (10 mL) with 0.25 mmol of acetobromo-α-d-glucose, 0.25 mmol of phenyl acetylene, 0.75 mmol of sodium azide, 1/1.5 mol% 1 (2/3 mol% [Cu]) in 2 mL of DES at r.t./70 °C.

b Isolated yields of triazole product A.

**Fig. 1:**
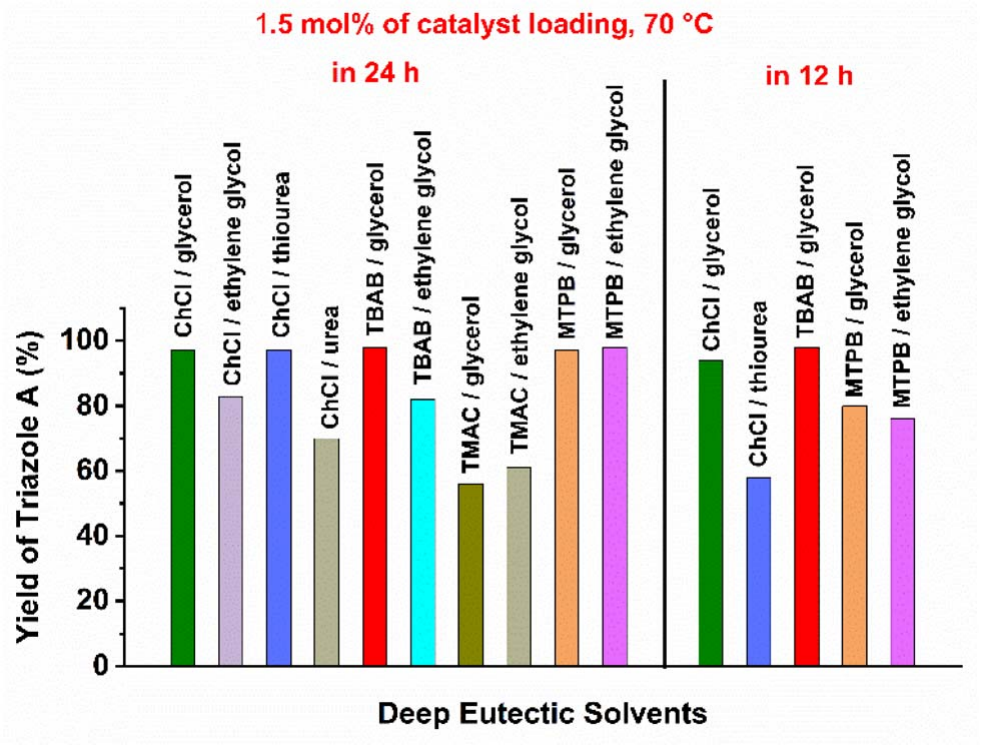
Comparative catalytic performance of complex 1 in various DESs.

With a readily available well-defined copper(i) complex in hand, we investigated the catalytic efficiency of 1 for a three component CuAAC reaction with phenyl acetylene, sodium azide and acetobromo-α-d-glucose as standard substrates (Table 1). Instead of using expensive and unstable organoazides, *in situ* generated organoazides by reacting sodium azide and halo-derivatives brings certain advantages. Thus, we focused on the three component CuAAC reaction to test the catalytic activity of complex 1 in various DESs (Table 1 and Fig. 1). For the first set of experiments, a mixture of choline chloride (ChCl) and glycerol (1 : 2), a heavily used and a very common DES, was used at r.t. Using 1.5 mol% catalyst loading (3 mol% [Cu]), we found only 34% yield of triazolyl glycoconjugate A in 24 h (entry 1). The triazole product A was analyzed by using ^1^H-NMR spectroscopy. The starting α-d-glucose is converted into a β-isomer of glucopyranosyltriazole *via in situ* formation of stable acetoazido-β-d-glucose. This initial catalytic activity of complex 1 in ChCl/glycerol (1 : 2) was found to be poor at r.t. Hence, the following CuAAC reactions were conducted at elevated temperature (70 °C). According to CHEM21 green metrics toolkit, 70 °C falls in the acceptable range of reaction temperature.^114^ To our satisfaction, quantitative yield of glucopyranosyltriazole A was obtained if the reaction was performed in ChCl/glycerol (1 : 2) at 70 °C in presence of 1.5 mol% of complex 1 (entry 2). Thereafter, we explored various hydrogen bond donors in DES preparation. ChCl/ethylene glycol (1 : 2) and ChCl/thiourea (1 : 2) as reaction medium resulted in 83% (entry 3) and quantitative (entry 4) yields of A, respectively. If we use a mixture of choline chloride and urea in 1 : 2 ratio, we found roughly 70% yields of desired triazole A under (entry 5). Thus, combination of ChCl as a hydrogen-bond acceptor, and glycerol or thiourea as hydrogen bond donors displayed better catalytic performance as compared to other ChCl based DESs. Next, we explored various other well-known DESs by varying both hydrogen-bond donors and hydrogen-bond acceptors. We tested six other DESs namely TBAB/glycerol (1 : 4), TBAB/ethylene glycol (1 : 4), TMAC/glycerol (1 : 2), TMAC/ethylene glycol (1 : 2), MTPB/glycerol (1 : 3), and MTPB/ethylene glycol (1 : 3). Under unaltered reaction conditions except various solvents (as used in entry 2 to 5), we found quantitative yields in case of TBAB/glycerol (1 : 4) (entry 6), MTPB/glycerol (1 : 3) (entry 10), and MTPB/ethylene glycol (1 : 3) (entry 11). In contrast, comparatively less catalytic efficiency was noted in TBAB/ethylene glycol (1 : 4) (80%, entry 7), TMAC/glycerol (1 : 2) (56%, entry 8) and TMAC/ethylene glycol (1 : 2) (60%, entry 9). Hence, it can be concluded that glycerol is an effective hydrogen-bond donor in the present CuAAC reaction. Thereafter, we turned our attention to those DESs which showed quantitative formation of A and explored the catalytic activity of complex 1 at reduced reaction time. We performed the reaction at 70 °C for 12 h in ChCl/glycerol (1 : 2), ChCl/thiourea (1 : 2), TBAB/glycerol (1 : 4), MTPB/glycerol (1 : 3) and MTPB/ethylene glycol (1 : 3) in presence of 1.5 mol% 1. We observed almost quantitative formation of A only in ChCl/glycerol (1 : 2) (entry 12) and TBAB/glycerol (1 : 4) (entry 14). In all other cases, we found incomplete conversion of starting materials and roughly 60 to 80% yields of A were obtained (entry 13: 58%, entry 15: 80% and entry 16: 76%). Thus, we concluded that ChCl/glycerol (1 : 2) and TBAB/glycerol (1 : 4) were the best reaction media as full conversion of substrates and quantitative yield of A were observed. Further reduction of reaction time to 10 and 8 h in TBAB/glycerol (1 : 4) resulted in incomplete conversions with small amount of unreacted starting materials (entry 17: 87% and entry 18: 75%). The catalyst loading was also reduced to 1 mol% in TBAB/glycerol (1 : 4) and again we found less yield of A (entry 19: 76%). If the reaction was conducted with 1.5 mol% of catalyst loading at r.t. for 12 h in TBAB/glycerol (1 : 4), we observed poor yield (entry 20: 30%). From these reaction optimizations, the best conditions were concluded as the following: entry 12: 1.5 mol% of catalyst loading, ChCl/glycerol (1 : 4), 70 °C, 12 h; entry 14: 1.5 mol% of catalyst loading, TBAB/glycerol (1 : 4), 70 °C, 12 h (entry 12 and 14: depicted in bold and green).

In this current work, we studied the possible use of DES as an emerging class of environmentally benign solvent for CuAAC reaction to synthesize glucopyranosyltriazole and we feel the necessity to evaluate the green and sustainable aspects of our optimized catalytic protocols with the help of “The 12 Principles of Green Chemistry”.^115–120^ In the present protocol for CuAAC reaction, we did not use any additive or reducing agent to stabilise or generate the copper(i) oxidation state, thus no doubt this is an advantage. A readily available, stable and well-defined copper(i)-iodide complex is directly used for three component cycloaddition reaction between acetobromo-α-d-glucose and phenyl acetylene in presence of sodium azide. Thereafter, the best optimized reaction conditions (entry 12 and 14 in Table 1) were evaluated with the help of CHEM21 green metrics toolkit which is a reckonable extension of “The 12 Principles of Green Chemistry”.^114^ The results of these two methods (Method A and B) are summarised in Table 2. In past, several research groups (including us) utilized this toolkit to determine the green and sustainable aspects of various catalytic transformations.^111,121–125^ Herein, we examined the optimized CuAAC reaction protocols by utilizing zero pass and first pass of CHEM21 green metrics toolkit. This toolkit also has second and third pass, but these are considered as industrial toolkits and outside the scope of the present study. The desirable and undesirable processes are highlighted by three different flags; green flag defines an acceptable process; amber flag stands for acceptable with concerns and red flag indicates undesirable process. First, we checked the metrics yield, conversion and selectivity. As both of the optimized reaction conditions gave full conversion, almost quantitative yields and excellent regioselectivity, green flags were assigned for all three metrics yield, conversion (conv.) and selectivity. High atom economy (A.E.), reaction mass efficiency (M.E.), optimum efficiency and mass intensity demonstrated high efficiency of both catalytic processes. Solvent is a crucial metric and we have used TBAB/glycerol (1 : 4) and ChCl/glycerol (1 : 2) as a metal-free type-III DES in our optimized protocols. As TBAB has potential issues as reflected with amber and red H-codes, method A received red flag for solvent metric. As both ChCl and glycerol as associated with green H-codes, solvent metric for method B earned green flag. As our methods are catalytic, they received green flag. However, we were unable to recover the catalyst. Thus, catalyst recovery metric received red flags both Method A and B. After completion of CuAAC reaction, triazole product binds copper center and the copper catalyst is lost with the product. After product isolation, the reaction medium was free of copper and thus, the catalyst could not be recycled. However, the reaction media were recovered successfully. The recovered DESs were reused and recycled five times to perform new sets of CuAAC reactions (see ESI† for details) and hence, this metric earned green flag. Although copper is very cheap and an earth-abundant base metal, worldwide availability of copper is dependent on geo-political issues. In that sense, copper as catalyst received amber flag in element metric. Next amber flag was assigned for reactor as we performed the reactions in batch. Generally, a reaction conducted in continuous flow process receives green flag. Then the work-up processes were analyzed. In our optimized catalytic protocols, we utilized very common and easy work-up techniques. The desired triazole product was simply isolated by dilution of the reaction media followed by filtration and washing with aq. ammonia solution. The isolated product was pure which does not require any further complex purification steps such as column chromatography. Thus, the present method is highly sustainable and assigned with green flag. Thereafter, the energy parameter of our catalytic protocol was considered. CHEM 21 green metrics toolkit also guided us in structuring a chemical process; an acceptable temperature range in terms of favorable energy is 0 to 70 °C. We have performed our reactions at 70 °C and hence, they earned green flag in energy metric. Overall, our catalytic protocols are sustainable as most of the metrices earn green flags. However, Method B with ChCl/glycerol (1 : 2) can be considered as a better one (green flag for solvent metric). Most importantly use of DESs as environmentally benign reaction media opens up a new pathway for the syntheses of important glycoconjugates.

**Table 2:** Calculation of different metrics of CuAAC reaction under optimized condition

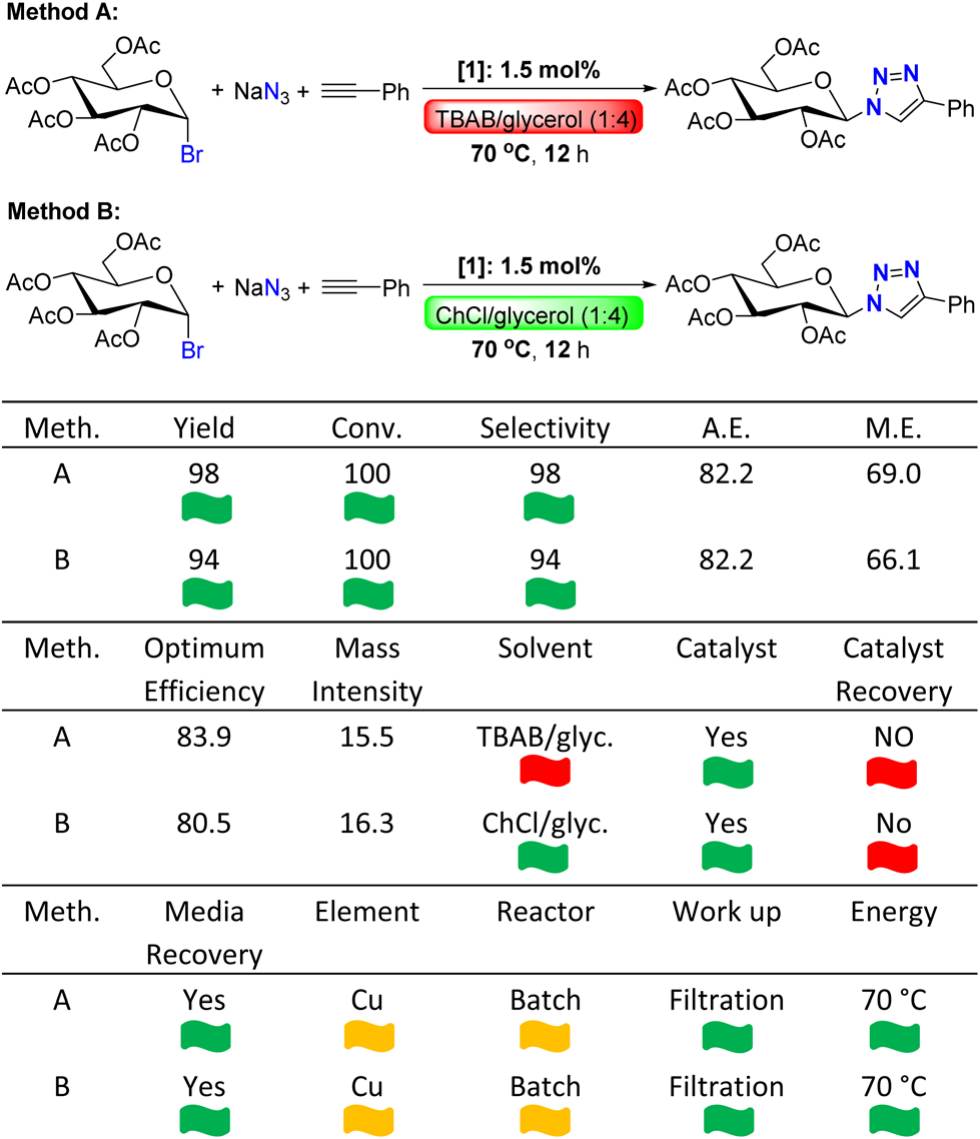

To achieve green and sustainable development, waste management (remaining starting materials, byproducts, unrecovered catalysts and solvent losses *etc.*) in chemical processes is a crucial factor. In this regard, *E*-factor gives us a general idea to estimate the quantity of waste generated for the production of one kilogram of desired product.^126,127^ Thus, calculation of *E*-factor is essential to evaluate real applicability and environmental acceptability of a chemical process. Usually, favourable *E*-factor should be in the range of 1 to 5. The calculated *E*-factors (see details in ESI†) of present optimized catalytic processes of CuAAC reactions are in the range of 2 to 4 (Method A: 2.21–3.02, Method B: 2.38–3.12), which reflects the potential applicability of the present optimized protocol for the bulk production of important glycoconjugates. Additionally, two gram-scale reactions were also effectively conducted using 10 mmol acetobromo-α-d-glucose and phenyl acetylene in presence of sodium azide (25 mmol) and we observed close to quantitative yields of desired triazole A. Hence, these catalytic methodologies illustrate the possible use of DES as a reuseable solvent for the syntheses of triazole equipped glycoderivatives (in contrast to the commonly use hazardous organic solvents) and this might also be realistic for probable industrial application.

**Scheme 3:**
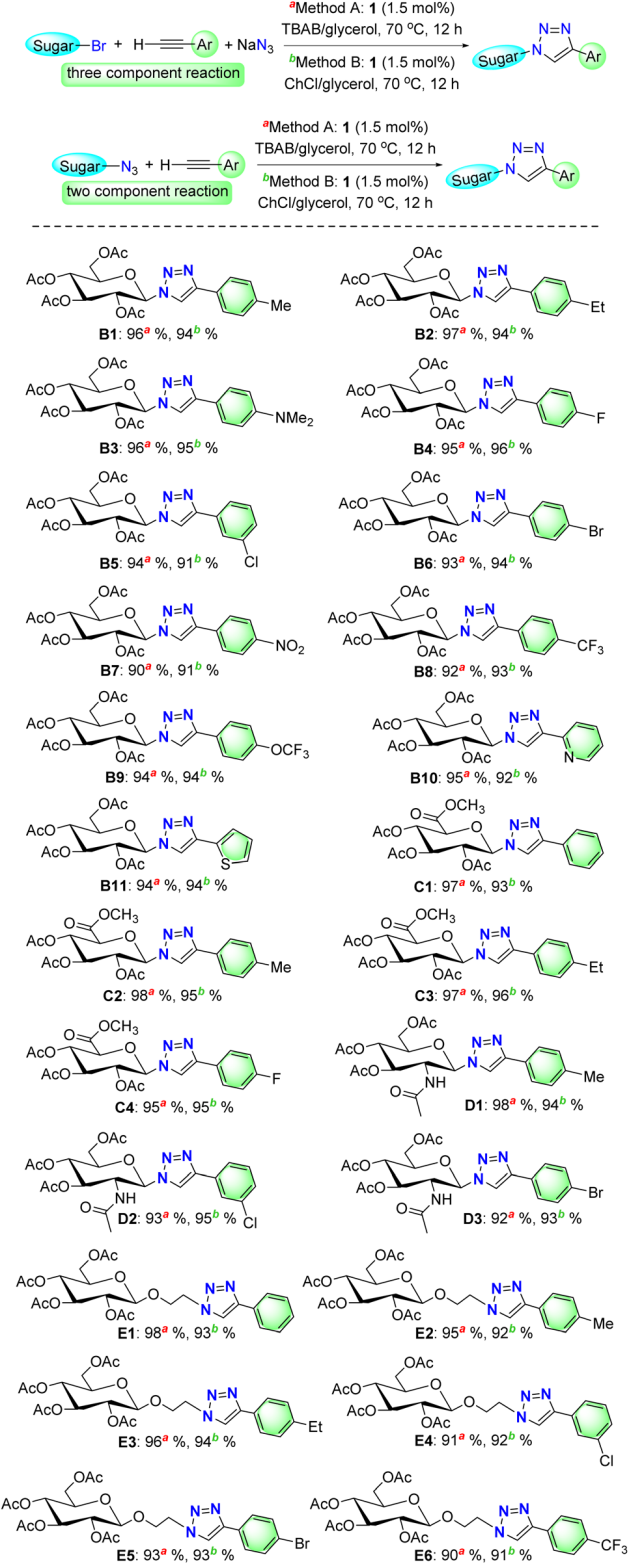
CuAAC reactions of various glycosides and terminal alkynes in TBAB/glycerol (1 : 4) and ChCl/glycerol (1 : 2) using optimized methods.

To probe the practical applicability of our approach, we explored the substrate scope under both of the optimized reaction conditions (Scheme 3). For this purpose, we carried out CuAAC reactions with the combination of four different glycosides containing halide or azide functionality. Halide compounds were *in situ* converted to the corresponding azides by reacting with sodium azide. We did not use sodium azide when the starting glycosides had an azide functionality. We also tested various terminal alkynes. Primarily under the optimized reaction conditions, acetobromo-α-d-glucose (in presence of sodium azide) was successfully clicked with different terminal alkynes having electron donating and withdrawing functionalities and we found excellent yields of various 1,4-disubstituted 1,2,3-glucopyranosyltriazoles (B1, B2, B3, B4, B5, B6, B7, B8, B9). Therefore, good tolerance to various electronic environment of the functional groups was observed. It is noteworthy to mention that all these triazolyl glyco-products were isolated by simple dilution of reaction media followed filtration and washing with aqueous ammonia solution. Thereafter, the combination of acetobromo-α-d-glucose with terminal alkynes having five- or six-member heterocycles also gave excellent yields of desired triazole products (B10, B11). Then, we tested another glycoside acetobromo-*α*-d-glucuronic acid methyl ester (at C5 position). Acetobromo-α-d-glucuronic acid methyl ester was also effectively clicked with various terminal alkynes with electron donating as well electron withdrawing groups and expected triazole products were obtained in excellent yields (C1, C2, C3, C4). Thereafter, we tested a glycoside which has acetamide substitution at C2 instead of acetoxy group. 2-Acetamido-2-deoxy-α-d-glucopyranosyl chloride 3,4,6-triacetate was also combined smoothly with various terminal alkynes. Good tolerance to various electronic environment was noted with excellent yields of triazole products (D1, D2, and D3). Finally, 2-azidoethyl 2,3,4,6-tetra-*O*-acetyl-β-d-glucopyranoside was tested to click with various terminal alkynes and we observed excellent yields of the corresponding triazoles (E1, E2, E3, E4, E5, E6). Thus, the electronic nature of the substituents has almost no effect in this CuAAC protocol catalyzed by complex 1 in DESs.

## Conclusions

Facile complexation of a tridentate NNO ligand L_1_ with copper iodide resulted in the formation a dimeric copper(i) complex 1. Complex 1 was characterised by standard techniques such as elemental analysis, mass spectrosmetry, IR and NMR spectroscopy and single crystal X-ray crystallography. Complex 1 proved to be an effective catalyst for the synthesis of numerous triazole equipped glycoconjugates using CuAAC reaction. This 1,3-cycloaddition of azido-glycose and terminal alkyne was performed in eleven different DESs. Among those, TBAB/glycerol (1 : 4) and ChCl/glycerol (1 : 2) were found to be the best reaction media and almost quantitative yields of the desired 1,4-disubstituted 1,2,3-glucopyranosyltriazoles product were achieved in 12 h at 70 °C in presence of 1.5 mol% of catalyst loading. This study showed, for the first time, the straightforward use of reusable and environmentally benign type-III DES as reaction medium for the syntheses of various glucopyranosyltriazoles using click chemistry. Purification of product by using very simple work-up procedure (filtration) is also advantageous. Although used copper catalyst could not be recovered, the used reaction media was reused several times. The sustainable features of the optimized reaction protocols were examined with the help of CHEM21 green metrics toolkit. Additionally, the catalytic protocols generated minimum waste as reflected by low *E*-factor of 2.21 < *E*-factor < 3.12. Thus, the present catalytic system is highly sustainable and opens up a new perspective in successful use of DES as a reaction medium in glyco-chemistry inspired by prototype click reaction.

## Author contributions

The manuscript was written through contributions of all authors. All authors have given approval to the final version of the manuscript.

## Conflicts of interest

There are no conflicts to declare.

## Supplementary Material

RA-013-D3RA01844J-s001

RA-013-D3RA01844J-s002

## References

[cit1] Rostovtsev V. V., Green L. G., Fokin V. V., Sharpless K. B. (2002). Angew. Chem., Int. Ed..

[cit2] Tornøe C. W., Christensen C., Meldal M. (2002). J. Org. Chem..

[cit3] Zhang B. (2019). Eur. J. Med. Chem..

[cit4] Prasher P., Sharma M. (2019). MedChemComm.

[cit5] Bonandi E., Christodoulou M. S., Fumagalli G., Perdicchia D., Rastelli G., Passarella D. (2017). Drug Discovery Today.

[cit6] Kluba C. A., Mindt T. L. (2013). Molecules.

[cit7] Moses J. E., Moorhouse A. D. (2007). Chem. Soc. Rev..

[cit8] Amblard F., Cho J. H., Schinazi R. F. (2009). Chem. Rev..

[cit9] Angell Y. L., Burgess K. (2007). Chem. Soc. Rev..

[cit10] El-Sagheer A. H., Brown T. (2010). Chem. Soc. Rev..

[cit11] Saha P., Panda D., Dash J. (2019). Chem. Commun..

[cit12] El-Sagheer A. H., Brown T. (2012). Acc. Chem. Res..

[cit13] Akter M., Rupa K., Anbarasan P. (2022). Chem. Rev..

[cit14] Golas P. L., Matyjaszewski K. (2010). Chem. Soc. Rev..

[cit15] Qin A., Lam J. W. Y., Tang B. Z. (2010). Chem. Soc. Rev..

[cit16] Nandivada H., Jiang X. W., Lahann J. (2007). Adv. Mater..

[cit17] Aromí G., Barrios L. A., Roubeau O., Gamez P. (2011). Coord. Chem. Rev..

[cit18] Kolb H. C., Sharpless K. B. (2003). Drug Discovery Today.

[cit19] Sharpless K. B., Manetsch R. (2006). Expert Opin. Drug Discovery.

[cit20] Thirumurugan P., Matosiuk D., Jozwiak K. (2013). Chem. Rev..

[cit21] Rani A., Singh G., Singh A., Maqbool U., Kaur G., Singh J. (2020). RSC Adv..

[cit22] Huang D., Zhao P., Astruc D. (2014). Coord. Chem. Rev..

[cit23] Ghosh R., Jana N. C., Panda S., Bagh B. (2021). ACS Sustainable Chem. Eng..

[cit24] Bagh B., McKinty A. M., Lough A. J., Stephan D. W. (2014). Dalton Trans..

[cit25] Bagh B., McKinty A. M., Lough A. J., Stephan D. W. (2015). Dalton Trans..

[cit26] Bagh B., Stephan D. W. (2014). Dalton Trans..

[cit27] Huisgen R. (1963). Angew. Chem., Int. Ed. Engl..

[cit28] Kolb H. C., Finn M. G., Sharpless K. B. (2001). Angew. Chem., Int. Ed..

[cit29] Meldal M., Tornøe C. W. (2008). Chem. Rev..

[cit30] Kowalsk K. (2023). Coord. Chem. Rev..

[cit31] Vivancos Á., Segarra C., Albrecht M. (2018). Chem. Rev..

[cit32] Alonso F., Moglie Y., Radivoy G. (2015). Acc. Chem. Res..

[cit33] Hein J. E., Fokin V. V. (2010). Chem. Soc. Rev..

[cit34] Liang L., Astruc D. (2011). Coord. Chem. Rev..

[cit35] Ladomenou G. K., Nikolaou V., Charalambidis G., Coutsolelos A. (2016). Coord. Chem. Rev..

[cit36] Meldal M. (2008). Macromol. Rapid Commun..

[cit37] Kappe C. O., Van der Eycken E. (2010). Chem. Soc. Rev..

[cit38] Rinaldi L., Martina K., Baricco F., Rotolo L., Cravotto G. (2015). Molecules.

[cit39] Gopalan B. and Balasubramanian K. K., Applications of Click Chemistry in Drug Discovery and Development, in Click Reactions in Organic Synthesis, ed. S. Chandrasekaran, Wiley-VCH, 2020, pp. 25−76

[cit40] Wang C., Ikhlef D., Kahlal S., Saillard J. Y., Astruc D. (2016). Coord. Chem. Rev..

[cit41] Hema K., Sureshan K. M. (2019). Acc. Chem. Res..

[cit42] Mandoli A. (2016). Molecules.

[cit43] Li P. Z., Wang X. J., Zhao Y. (2019). Coord. Chem. Rev..

[cit44] Chandrasekeran S. , Click Reactions in Organic Synthesis, John Wiley & Sons, New York, 2016

[cit45] Kumar G. S., Lin Q. (2021). Chem. Rev..

[cit46] Tiwari V. K., Mishra B. B., Mishra K. B., Mishra N., Singh A. S., Chen X. (2016). Chem. Rev..

[cit47] Agrahari A. K., Bose P., Jaiswal M. K., Rajkhowa S., Singh A. S., Hotha S., Mishra N., Tiwari V. K. (2021). Chem. Rev..

[cit48] Dondoni A., Marra A. (2010). Chem. Rev..

[cit49] Chandrasekaran V., Lindhorst T. K. (2012). Chem. Commun..

[cit50] Kufleitner M., Haibe L. M., Wittmann V. (2023). Chem. Soc. Rev..

[cit51] Kushwaaha D., Pandey P., Kale R. R., Tiwari V. K. (2012). Trends Carbohydr. Res..

[cit52] Cecioni S., Imberty A., Vidal S. (2015). Chem. Rev..

[cit53] Oliva C. G., Merchán A., Perona A., Hoyos P., Rumbero Á., Hernáiz M. J. (2022). New J. Chem..

[cit54] Mishra A., Tiwari V. K. (2015). J. Org. Chem..

[cit55] Kuijpers B. H. M., Groothuys S., Keereweer A. (B. ) R., Quaedflieg P. J. L. M., Blaauw R. H., van Delft F. L., Rutjes F. P. J. T. (2004). Org. Lett..

[cit56] Dondoni A., Marra A. (2006). J. Org. Chem..

[cit57] Potopnyk M. A. and Jarosz S., Sweet Sucrose Macrocycles via a Click Chemistry Route, in Click Chemistry in Glycoscience, New Developments and Strategies, ed. Witczak, Z. J. and Bielski, R., John Wiley and Sons, 2013, pp. 235−252

[cit58] Isobe H., Cho K., Solin N., Werz D. B., Seeberger P. H., Nakamura E. (2007). Org. Lett..

[cit59] Varki A. (1993). Glycobiology.

[cit60] Bertozzi C. R., Kiessling L. L. (2001). Science.

[cit61] Angata T., Varki A. (2002). Chem. Rev..

[cit62] Schmidt R. R., Kinzy W. (1994). Adv. Carbohydr. Chem. Biochem..

[cit63] Tiwari V. K., Mishra R. C., Sharma A., Tripathi R. P. (2012). Mini-Rev. Med. Chem..

[cit64] Kempe K., Krieg A., Becer C. R., Schubert U. S. (2012). Chem. Soc. Rev..

[cit65] Kuijpers B. H. M., Groothuys S., Hawner C., ten Dam J., Quaedflieg P. J. L. M., Schoemaker H. E., van Delft F. L., Rutjes F. P. J. T. (2008). Org. Process Res. Dev..

[cit66] Lee D. J., Mandal K., Harris P. W. R., Brimble M. A., Kent S. B. H. (2009). Org. Lett..

[cit67] Rabuka D., Hubbard S. C., Laughlin S. T., Argade S. P., Bertozzi C. R. (2006). J. Am. Chem. Soc..

[cit68] Tang S., Wang Q., Guo Z. (2010). Chem.–Eur. J..

[cit69] Joosten J. A. F., Tholen N. T. H., Ait El Maate F., Brouwer A. J., van Esse G. W., Rijkers D. T. S., Liskamp R. M. J., Pieters R. J. (2005). Eur. J. Org. Chem..

[cit70] Deraedt C., Pinaud N., Astruc D. (2014). J. Am. Chem. Soc..

[cit71] Tasca E., La Sorella G., Sperni L., Strukul G., Scarso A. (2015). Green Chem..

[cit72] Brotherton W. S., Michaels H. A., Simmons J. T., Clark R. J., Dalal N. S., Zhu L. (2009). Org. Lett..

[cit73] Pachón L. D., van Maarseveen J. H., Rothenberg G. (2005). Adv. Synth. Catal..

[cit74] Kamata K., Nakagawa Y., Yamaguchi K., Mizuno N. (2008). J. Am. Chem. Soc..

[cit75] Meng J.-c., Fokin V. V., Finn M. G. (2005). Tetrahedron Lett..

[cit76] Nallagangula M., Namitharan K. (2017). Org. Lett..

[cit77] Hein J. E., Tripp J. C., Krasnova L. B., Sharpless K. B., Fokin V. V. (2009). Angew. Chem., Int. Ed..

[cit78] Rasina D., Lombi A., Santoro S., Ferlin F., Vaccaro L. (2016). Green Chem..

[cit79] Luciani L., Goff E., Lanari D., Santoroa S., Vaccaro L. (2018). Green Chem..

[cit80] Ahmady A. Z., Heidarizadeh F., Keshavarz M. (2013). Synth. Commun..

[cit81] Martins M. A. P., Paveglio G. C., Rodrigues L. V., Frizzo C. P., Zanatta N., Bonacorso H. G. (2016). New J. Chem..

[cit82] Nolan M. D., Mezzetta A., Guazzelli L., Scanlan E. M. (2022). Green Chem..

[cit83] Giofrè S. V., Tiecco M., Ferlazzo A., Romeo R., Ciancaleoni G., Germani R., Iannazzo D. (2021). Eur. J. Org. Chem..

[cit84] Sethi S., Jana N. C., Behera S., Behera R. R., Bagh B. (2023). ACS Omega.

[cit85] Perez-Balderas F., Ortega-Munoz M., Morales-Sanfrutos J., Hernandez-Mateo F., Calvo-Flores F. G., Calvo-Asin J. A., Isac-Garcia J., Santoyo-González F. (2003). Org. Lett..

[cit86] Kumar D., Mishra K. B., Mishra B. B., Mondal S., Tiwari V. K. (2014). Steroids.

[cit87] Dwivedi P., Mishra K. B., Mishra B. B., Singh N., Singh R. K., Tiwari V. K. (2015). Glycoconjugate J..

[cit88] Mishra K. B., Shashi S., Tiwari V. K. (2016). Trends Carbohydr. Res..

[cit89] Mishra A., Tiwari V. K. (2017). ChemistrySelect.

[cit90] Dwivedi P., Mishra K. B., Mishra B. B., Tiwari V. K. (2017). Glycoconjugate J..

[cit91] Kushwaha D., Tiwari V. K. (2017). J. Heterocycl. Chem..

[cit92] Mishra K. B., Mishra R. C., Tiwari V. K. (2015). RSC Adv..

[cit93] Mishra K. B., Tiwari N., Bose P., Singh R., Rawat A. K., Mishra R. C., Singh R. K., Tiwari V. K. (2019). ChemistrySelect.

[cit94] Agrahari A. K., Singh A. S., Singh A. K., Mishra N., Singh M., Prakash P., Tiwari V. K. (2019). New J. Chem..

[cit95] Kumari K., Singh A. S., Manar K. K., Yadav C. L., Tiwari V. K., Drew M. G. B., Singh N. (2019). New J. Chem..

[cit96] Kushwaha A., Agrihari A. K., Manar K., Yadav C., Tiwari V. K., Drew M. G. B., Singh N. (2019). New J. Chem..

[cit97] Marra A., Vecchi A., Chiappe C., Melai B., Dondoni A. (2008). J. Org. Chem..

[cit98] Alonso D. A., Baeza A., Chinchilla R., Guillena G., Pastor I. M., Ramón D. J. (2016). Eur. J. Org. Chem..

[cit99] Abbott A. P., Capper G., Davies D. L., Rasheed R. K., Tambyrajah V. (2003). Chem. Commun..

[cit100] Smith E. L., Abbott A. P., Ryder K. S. (2014). Chem. Rev..

[cit101] Nebra N., García-Álvarez J. (2020). Molecules.

[cit102] Xiong X., Yi C., Liao X., Lai S. (2019). Tetrahedron Lett..

[cit103] Kafle A., Handy S. T. (2017). Tetrahedron.

[cit104] Afonso J., Mezzetta A., Marrucho I. M., Guazzelli L. (2023). Green Chem..

[cit105] Díez-González S. (2011). Catal. Sci. Technol..

[cit106] Díez-González S., Nolan S. P. (2008). Angew. Chem., Int. Ed..

[cit107] Collinson J. M., Wilton-Ely J. D. E. T., Díez-González S. (2013). Chem. Commun..

[cit108] Rodionov V. O., Presolsky S. I., Gardinier S., Lim Y. H., Finn M. G. (2007). J. Am. Chem. Soc..

[cit109] Sareen N., Singh A. S., Tiwari V. K., Kant R., Bhattacharya S. (2017). Dalton Trans..

[cit110] Joshi D. K., Mishra K. B., Tiwari V. K., Bhattacharya S. (2014). RSC Adv..

[cit111] Ghosh R., Behera R. R., Panda S., Behera S. K., Jana N. C., Bagh B. (2022). ChemCatChem.

[cit112] Behera R. R., Panda S., Ghosh R., Kumar A. A., Bagh B. (2022). Org. Lett..

[cit113] Hansen B. B., Spittle S., Chen B., Poe D., Zhang Y., Klein J. M., Horton A., Adhikari L., Zelovich T., Doherty B. W., Gurkan B., Maginn E. J., Ragauskas A., Dadmun M., Zawodzinski T. A., Baker G. A., Tuckerman M. E., Savinell R. F., Sangoro J. R. (2021). Chem. Rev..

[cit114] McElroy C. R., Constantinou A., Jones L. C., Summerton L., Clark J. H. (2015). Green Chem..

[cit115] Green Chemistry: Theory and Practice, ed. P. Anastas and J. C. Warner, Oxford University Press, Oxford, 1998

[cit116] Anastas P., Eghbali N. (2010). Chem. Soc. Rev..

[cit117] Sheldon R. A. (2012). Chem. Soc. Rev..

[cit118] Prat D., Wells A., Hayler J., Sneddon H., McElroy C. R., Abou-Shehada S., Dunn P. J. (2016). Green Chem..

[cit119] Allen D. T., Gathergood N., Licence P., Subramaniam B. (2020). ACS Sustainable Chem. Eng..

[cit120] Kuok Hii K., Moores A., Pradeep T., Sels B., Allen D. T., Licence P., Subramaniam B. (2020). ACS Sustainable Chem. Eng..

[cit121] Jana N. C., Sethi S., Saha R., Bagh B. (2022). Green Chem..

[cit122] Allen D. T., Carrier D. J., Gong J., Hwang B.-J., Licence P., Moores A., Pradeep T., Sels B., Subramaniam B., Tam M. K. C., Zhang L., Williams R. M. (2018). ACS Sustainable Chem. Eng..

[cit123] Droesbeke M. A., Du Prez F. E. (2019). ACS Sustainable Chem. Eng..

[cit124] Prieschl M., García-Lacuna J., Munday R., Leslie K., O'Kearney-McMullan A., Hone C. A., Kappe C. O. (2020). Green Chem..

[cit125] Dalidovich T., Mishra K. A., Shalima T., Kudrjašova M., Kananovich D. G., Aav R. (2020). ACS Sustainable Chem. Eng..

[cit126] Sheldon R. A. (2007). Green Chem..

[cit127] Lam C. H., Escande V., Mellor K. E., Zimmerman J. B., Anastas P. T. (2019). J. Chem. Educ..

