# Supplementary material for: Copper(i)-catalyzed click chemistry in deep eutectic solvent for the syntheses of β-d-glucopyranosyltriazoles
Source: RSC Adv. 2023 Apr 3;13(15):10424–32. doi: 10.1039/d3ra01844j (PMC10069229; doi:10.1039/d3ra01844j)
Supplement: RA-013-D3RA01844J-s001 [file RA-013-D3RA01844J-s001.pdf]

Electronic Supplementary Material (ESI) for RSC Advances.
This journal is © The Royal Society of Chemistry 2023

Supporting Information

Copper(I)-Catalyzed Click Chemistry in Deep Eutectic Solvent for the Syntheses of β-D-Glucopyranosyltriazoles

Subrat Sethi,[1] Narayan Ch. Jana,[1] Surajit Panda,[1] Suraj Kumar Maharana[1] and Bidraha Bagh *[1]

[1]School of Chemical Sciences, National Institute of Science Education and Research (NISER), An OCC of Homi Bhabha National Institute, Bhubaneswar, Jatni, Khurda, Odisha, PIN 752050, India.

*Corresponding author, E-mail: bidraha@niser.ac.in

Number of pages: 55

Number of figures: 59

Number of tables: 7

Table of Contents

General experimental	S3
Synthesis of ligand **L_1** and copper(I) complex **1**	S4
Characteristic spectra of ligand **L_1** and copper(I) complex **1**	S6
General experiments of CuAAC reactions in DESs	S9
NMR data of the products	S13
NMR spectra of the products	S21
Molecular structure determination by single crystal X-ray crystallography	S45
Zero Pass of optimized reaction condition by the CHEM21 green metrics toolkit	S49
First Pass of optimized reaction condition by the CHEM21 green metrics toolkit	S50
References	S55

General experimental

The catalytic reactions were performed under nitrogen atmosphere. Chemicals were purchased from Sigma-Aldrich, TCI, Alfa-aesar, Himedia chemicals and used without further purification. Solvents, purchased from commercial suppliers were dried prior to synthesis of copper complexes. Copper catalysed azide alkyne cycloaddition reaction (CuAAC) were performed under inert condition while deep eutectic solvents were prepared inside the globe box. Deuterated solvents DMSO-d_6 and $CDCl_3$ were purchased from Sigma-Aldrich and used without further purification for recording NMR spectra. ^{1}H and ^{13}C NMR spectra were recorded at Bruker AV-400 and 700 spectrometers (^{1}H at 400 MHz and ^{13}C at 101 MHz). ^{1}H NMR chemical shifts are referenced in parts per million (ppm) with respect to tetramethyl silane (δ 0.00 ppm) and $^{13}C\{^{1}H\}$ NMR chemical shifts are referenced in ppm with respect to DMSO-d_6 (δ 39.52 ppm) and $CDCl_3$ (δ 77.16 ppm). The coupling constants (*J*) are reported in hertz (Hz). The following abbreviations are used to describe multiplicity: bs = broad signal, s = singlet, d = doublet, t = triplet, q = quadrate, m = multiplate. High resolution mass spectra were recorded on a Bruker micrOTOF-Q II Spectrometer. Elemental analysis was carried out on a EuroEA Elemental Analyser. Crystal data were collected with Rigaku Oxford diffractometer and with INCOATEC micro source (Mo-Kα radiation, λ = 0.71073 Å, multilayer optics) at 100 K.

Synthesis of ligand L_1 and copper(I) complex 1

Synthesis of ligand L_1:[S1] Compound 3-(2-methoxyphenyl)-1H-pyrazole is a known compound and was synthesized using following procedure. A solution of 2-Methoxy acetophenone (1.50 g, 10.0 mmol) in a 1:1 mixture of DMF-DMA (15 mL) was reflux for 3 days under inert condition to give an orange brown solution. Removal of solvents under vacuum gave a brown oil. Thereafter, ethanol (20 mL) and hydrazine hydrate (1.28 g, 40.0 mmol) were added and the reaction mixture was refluxed for 2 hours. The resulting yellow solution was cooled to room temperature and cold water (15 mL) was added giving an off-white precipitate. The mixture was kept at 0-4 °C overnight to allow complete precipitation and the solid was collected after filtration. The solid was washed with cold water (3 x 20 mL) to give a white solid as pure product (1.67 g, 96%). ^{1}H NMR ($CDCl_3$): δ 12.50 (br, 1H), 7.73 (m, 1H), 7.69 (s, 1H), 7.09 (m, 2H), 6.67 (s, 1H), 4.02 (s, 3H).

In a pressure tube, 3-(2-methoxyphenyl)-1H-pyrazole (1.74 g, 10.0 mmol), 2-(chloromethyl) pyridine hydrochloride (1.64 g,10.0 mmol), NaOH solution (40%, 5 mL) and toluene (15 mL) were added and the reaction mixture was stirred at r.t. for 15 mins. Then tert-butylammonium hydroxide (4 mL) was added and the reaction mixture was stirred at 130 °C temperature for 24 h. The resulting red mixture was cooled down to r.t. and extracted with ethyl acetate (3 x 50 mL). All volatiles were removed under high vacuum to give 2-((3-(2-methoxyphenyl)-1H-pyrazol-1-yl) methyl) pyridine as pure product (2.27 g, 73%).[S1] ^{1}H NMR (400 MHz, $CDCl_3$) δ 8.59 (s, 1H), 7.98 (dd, *J* = 7.7, 1H), 7.62–7.64 (m, *J* = 7.7, 1H), 7.56 (d, *J* = 2.3 Hz, 1H), 7.28–7.30 (m, *J* = 8.2, 1.7 Hz, 1H), 7.20 (dd, *J* = 7.0, Hz, 1H), 7.08–6.96 (m, 3H), 6.87 (d, *J* = 2.3 Hz, 1H), 5.53 (s, 2H), 3.91 (s, 3H). ^{13}C NMR (101 MHz, $CDCl_3$) δ 157.13, 156.87, 149.34, 149.01, 137.20, 130.53, 128.87, 122.76, 122.45, 121.83, 120.91, 111.39, 107.79, 57.73, 55.57. HRMS (ESI-TOF) m/z: $[M + H]^+$ calcd for $C_{16}H_{16}N_3O$ 266.1293; found 266.1285. Anal. Calcd

for $C_{16}H_{15}N_3O$ (265.12): C, 72.43; H, 5.70; N, 15.84. Found: C, 72.25; H, 5.56; N, 15.68. FTIR ν_{max} (cm^{-1}): 2800–3100 (C–H), 1505 (N−N), 1410–1600 (C=N, py; C=C, ph).

Synthesis of copper (I) complex 1

The ligand (0.066 g, 0.25 mmol) was dissolved in 5 mL CH_3CN in a Schlenk tube inside the glovebox. CuI (0.047 g, 0.25 mmol) suspension in acetonitrile was then added to the ligand solution with continuous stirring. The reaction mixture turned into whitish color solution and the stirring was continued for 24 hours. The reaction mixture was then dried in high vacuum and the NMR of the crude solid was recorded in DMSO-d_6. The crude solid was then dissolved in DMF and taken into a 5 mL vial. The crystallization was carried out by diffusing with diethyl ether. Block shape white crystals suitable for X-ray crystallography were grown at the bottom of the vial after two days. Yield: (0.093 g, 82%). ^{1}H NMR (700 MHz, DMSO) δ 8.51 (s, 1H), 8.03 (s, 1H), 7.95 (t, J = 7.5 Hz, 1H), 7.84 (d, J = 6.5 Hz, 1H), 7.57 (s, 1H), 7.44 (s, 1H), 7.30 (t, J = 7.3 Hz, 1H), 7.03 (d, J = 8.3 Hz, 1H), 6.88 (t, J = 7.3 Hz, 1H), 6.69 (s, 1H), 5.55 (s, 2H), 3.75 (s, 3H). ^{13}C NMR (101 MHz, DMSO) δ 156.47, 154.19, 149.78, 148.38, 138.51, 132.36, 129.62, 129.51, 124.14, 123.97, 120.89, 120.14, 111.49, 107.21, 55.42, 54.92. Anal. Calcd. for $C_{32}H_{30}Cu_2I_2N_6O_2$ (911.50): C, 47.17; H, 3.32; N, 9.22; Found: C, 47.03; H, 3.37; N, 9.10. HRMS (ESI-TOF) m/z: $[M]^+$ calcd for $C_{16}H_{15}CuN_3O$ 328.0511; found 328.0517. FTIR ν_{max} (cm^{-1}): 2740–3130 (C–H), 1515 (N–N), 1400–1640 (C=N, py; C=C, ph).

Characteristic spectra of ligand L_1 and copper(I) complex 1

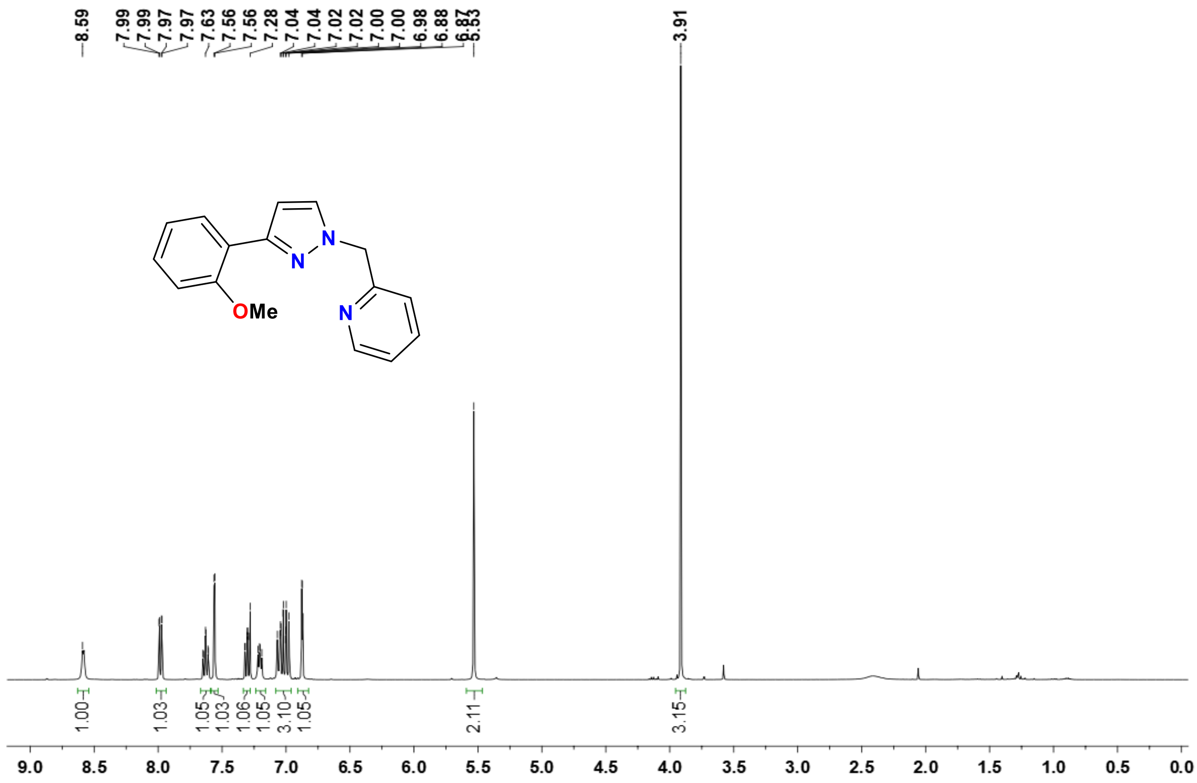

Figure S1. ^{1}H NMR of 2-((3-(2-methoxyphenyl)-1H-pyrazol-1-yl) methyl)pyridine (**L_1**) in $CDCl_3$ at r.t.

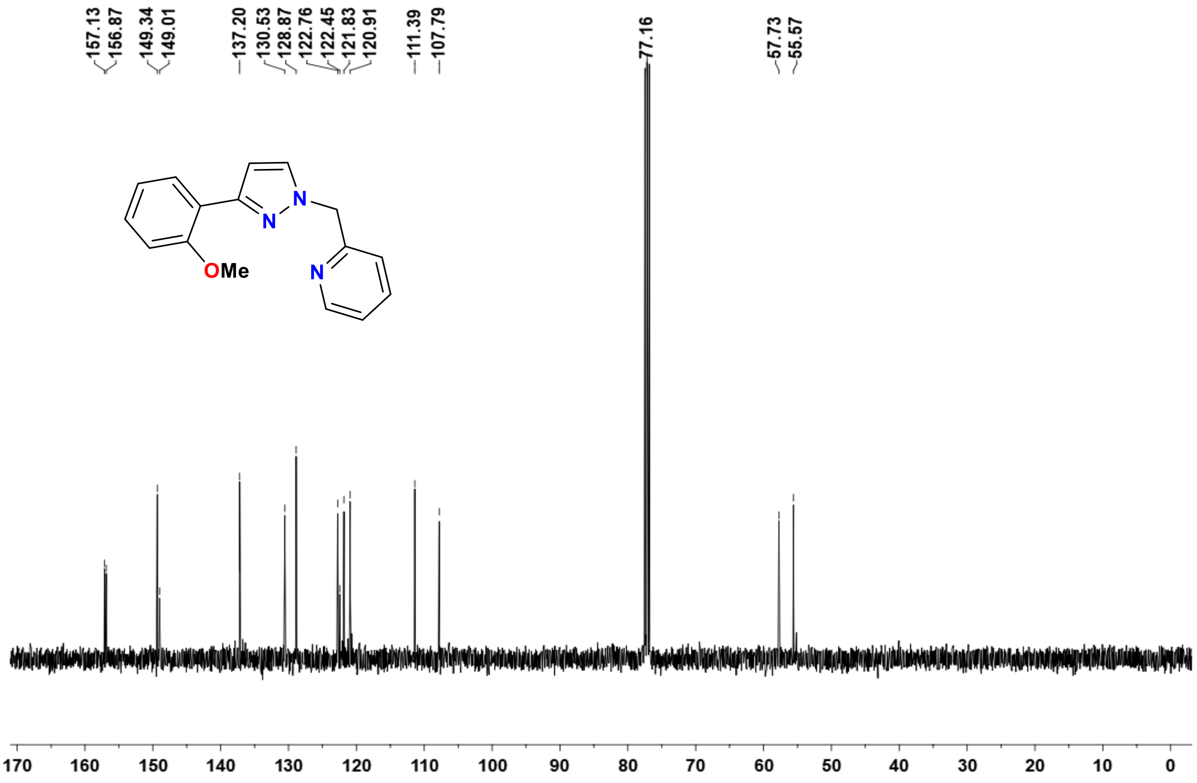

Figure S2. ^{13}C NMR of 2-((3-(2-methoxyphenyl)-1H-pyrazol-1-yl) methyl)pyridine (**L_1**) in $CDCl_3$ at r.t.

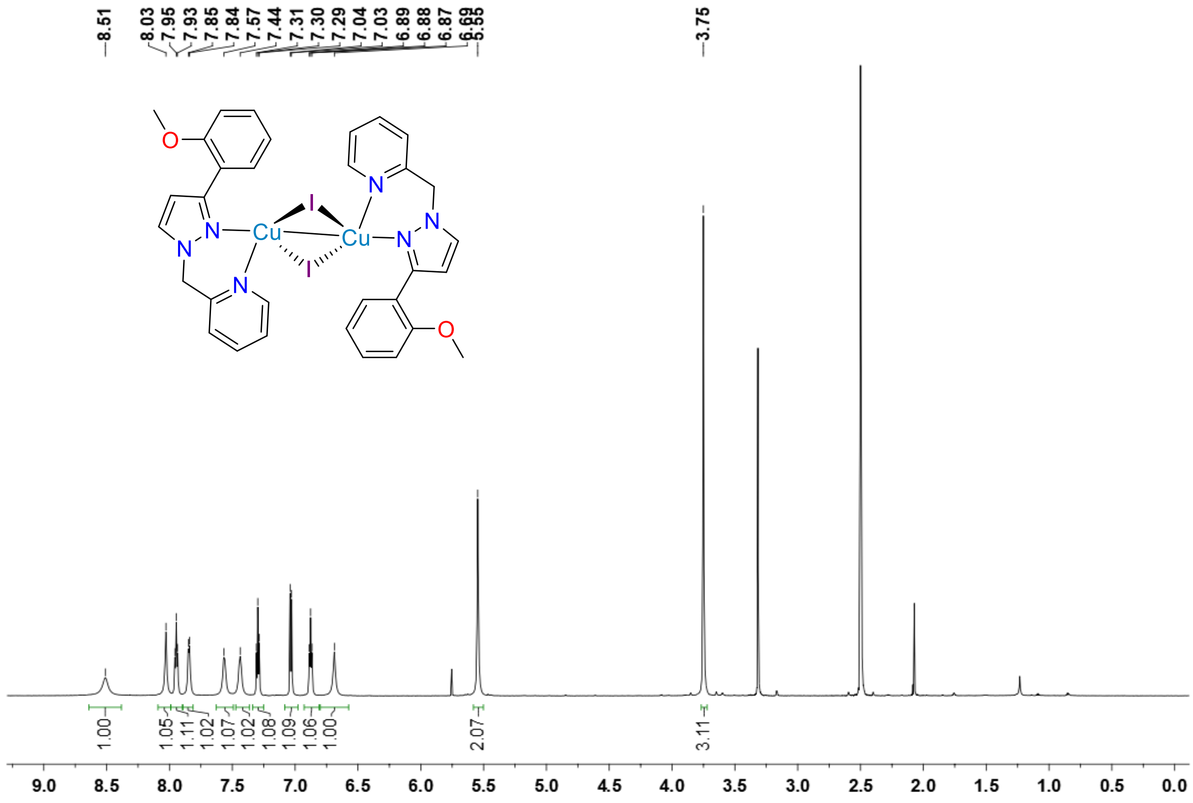

Figure S3: ^{1}H NMR of complex **1** in DMSO-d$_6$.

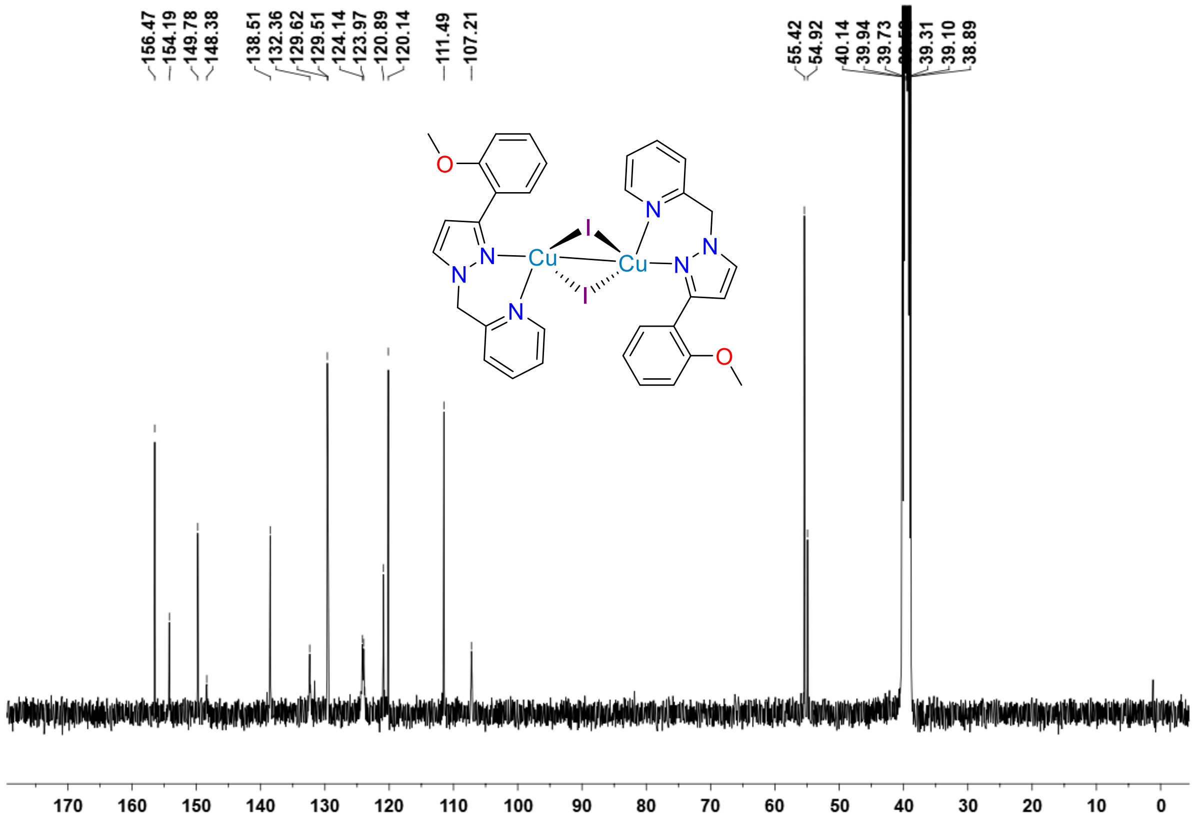

Figure S4: ^{13}C NMR of complex **1** in DMSO-d$_6$.

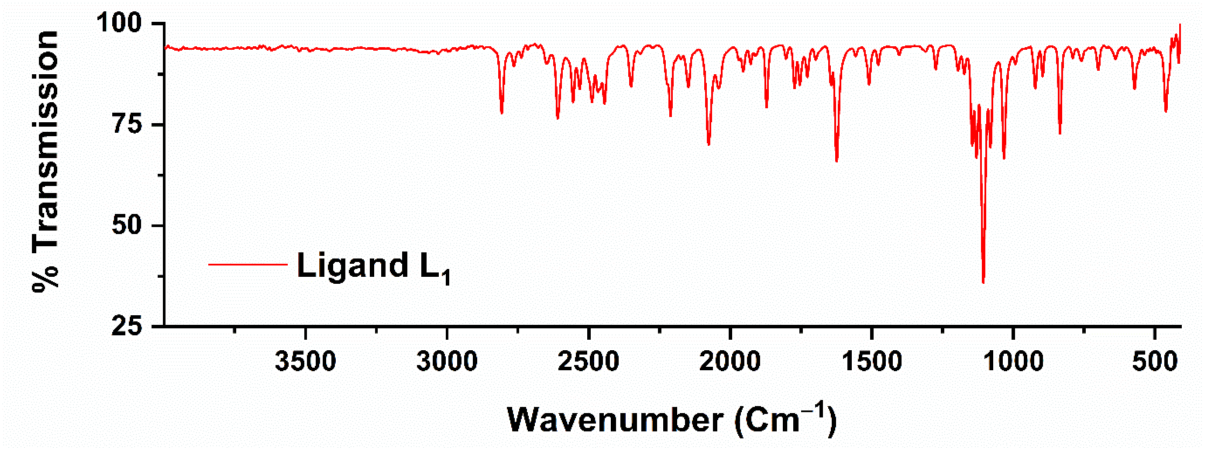

Figure S5: FTIR Spectrum of ligand $\mathbf{L_1}$.

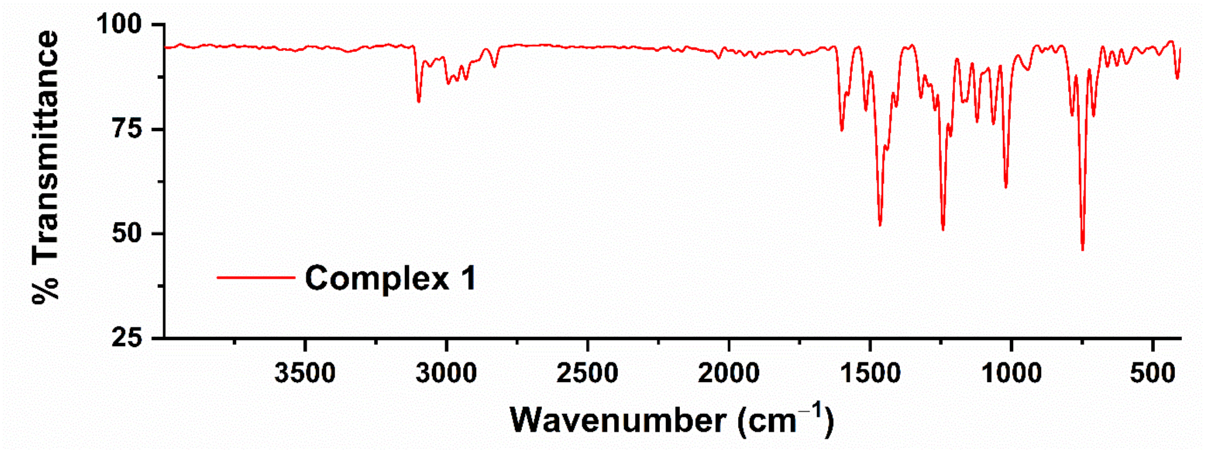

Figure S6: FTIR Spectrum of complex **1**.

General experiments of CuAAC reactions in DESs

General procedure for CuAAC reactions (for reaction optimisation).

All the CuAAC reactions were performed under inert condition. 1-Bromo-α-D-glucose tetraacetate, 2,3,4,6-Tetra-O-acetyl-α-D-glucopyranosyl bromide (0.102 g, 0.25 mmol), phenyl acetylene (0.025 g, 0.25mmol), NaN_3 (0.040 g, 0.625 mmol), and 1.5 mol% copper complex **1** (3 mol% [Cu]), were weighed in a vial (10 mL). Thereafter, different deep eutectic solvent for different experiments was added to it. The resultant mixture was heated at appropriate temperature (r.t./ 70 °C) for an appropriate time with stirring. Formation of solid crude was observed after the desire reaction time. The crude solid product was then filtered and washed with additional 2 mL of 25 % ammonia solution to remove copper from the triazole products. The compound was then dried under vacuum and white crude solid compound was isolated as pure form. The product was dissolved in $CDCl_3$ and ^1H NMR spectrum was recorded. The anomeric configuration of the triazole was compared with literature reported compound and the NMR data/spectra of our products are identical with the reported β-isomer of glucopyranosyltriazoles.[S2] The NMR signature of the corresponding α-D-Glucopyranosyltriazole is different.[S3]

General procedure for gram-scale CuAAC reactions:

The optimised reaction conditions in TBAB/glycerol (Method **A**; 1.5 mol% of catalyst loading, ChCl/glycerol (1:2), 70 °C, 12 h) or in ChCl/glycerol (Method **B**; 1.5 mol% of catalyst loading, ChCl/glycerol (1:2), 70 °C, 12 h) was selected for gram-scale (10 mmol) CuAAC reactions as reaction medium. For this purpose, acetobromo-α-D-glucose (4.112 g, 10 mmol), phenyl acetylene (1.021 g, 10 mmol), NaN_3 (1.625 g, 25 mmol), and copper complex **1** (1.5 mol%), were added in 40 mL of TBAB/glycerol (1:4) or ChCl/glycerol (1:2). After the desired reaction time the crude solid product was filtered and washed with additional 20 mL of 25 % ammonia solution. The compound was then dried under vacuum and white crude solid compound was isolated as pure form. The product was then dissolved in $CDCl_3$ and ^1H NMR spectrum was recorded.

General procedure for substrate scope:

Both the optimised reaction conditions (Method A; 1.5 mol% of catalyst loading, TBAB/glycerol (1:4), 70 °C, 12 h; Method B; 1.5 mol% of catalyst loading, ChCl/glycerol (1:2), 70 °C, 12 h) were utilized for the synthesis of various *β*-D-glucopyranosyltriazoles. In this regard, four different glucopyran-derivatives (0.25 mmol), and alkynes (0.25 mmol) were combined with sodium azide (0.62 mmol) and copper complex **1** (1.5 mol %) in a vial (10 mL).

It is important to mention that, we did not use sodium azide when the starting glycosides had an azide functionality i.e. the substrates scope with 2-azidoethyl 2,3,4,6-tetra-O-acetyl-β-D-glucopyranoside. Thereafter, DESs ChCl/glycerol or TBAB/glycerol (1:4) (2 mL) was added to it. The reaction mixture was placed under optimized reaction conditions. After that, the reaction mixture was diluted with water followed by filtered and washed with 25 % ammonia solution to get desire *β*-D-glucopyranosyltriazoles product. The compound was then dried under vacuum and crude solid compound was isolated as pure form. Occasionally the crude product was purified by column chromatography using silica as stationary phase and ethyl acetate-hexane mixture (1:9) as eluent. The product was then dissolved in $CDCl_3$ and NMR (^1H and ^{13}C) spectrum was recorded. Most of the products glucopyranosyltriazoles (β-isomer) are reported compounds and the NMR data/spectra of our products are identical with the reported compounds.[S2] The NMR signature of the corresponding α-D-Glucopyranosyltriazole is different.[S3]

General procedure for the recycling of the reaction medium and calculation of *E*-factor: The reaction medium was recycled as follows: For this purpose, 2 mmol reaction scale was conducted with, acetobromo-α-D-glucose (0.822 g, 2 mmol), phenyl acetylene (0.204 g, 2 mmol), NaN_3 (0.325 g, 5 mmol), and 1.5 mol% copper complex **1** (3 mol% [Cu]), using 4 mL (4.788 g) of TBAB/glycerol (1:4) or ChCl/glycerol (1:4) as reaction medium. The solid product was filtered with a Büchner funnel, washed with 25 % ammonia solution (2 mL) by portion and dried under vacuum. Thereafter, the volume of the aqueous phase was reduced to 4 mL and placed in the reaction vial equipped with a magnetic stirring bar. Acetobromo-α-D-glucose (0.102 g, 0.25 mmol), phenyl acetylene (0.025 g, 0.25mmol), NaN_3 (0.016 g, 0.25 mmol), and 1.5 mol% copper complex **1** (3 mol% [Cu]) were added and the reaction was stirred for 12 h at 70 °C. Here it is worth to mention that only 1 eqv. amount of sodium azide is used instead of 2.5 eqv. as we assumed that recovered reaction media already contain 1.5 eqv sodium azide. The entire process was repeated four times. Thus, the reaction medium was recycled five times and no change in catalytic activity was observed. The amount of media recovered in both the methods is as follows: **Method A**: 1st run: 4.726 g; 2nd cycle: 4.672 g; 3rd cycle: 4.545 g; 4th cycle: 4.320 g; 5th cycle: 4.150 g; **Method B**: 1st run: 4.776 g; 2nd cycle: 4.650 g; 3rd cycle: 4.625 g; 4th cycle: 4.598 g; 5th cycle: 4.550 g.

The triazole product **A** was obtained in excellent isolated yield in all reaction runs: **Method A**: (1st run: 931 mg; 2nd cycle: 921 mg; 3rd cycle: 921 mg; 4th cycle: 931 mg; 5th cycle: 921 mg);

Method B: (1st run: 893 mg; 2nd cycle: 903 mg; 3rd cycle: 892 mg; 4th cycle: 897 mg; 5th cycle: 905 mg).

Overall E-factor for five consecutive reaction runs in Method A and Method B

Method A: 1.5 mol% of catalyst loading, 70 °C, 12 h in TBAB/glycerol (1:4)

Substrate:	acetobromo-α-D-glucose (2 mmol)	= 0.822 g
	Phenyl acetylene (2 mmol)	= 0.204 g
	Sodium azide (5 mmol)	= 0.325 g
Catalyst:	Cu(I) iodide complex **1** (1.5 mol %)	= 0.013 g
Solvent:	TBAB/glycerol (4 mL) x 1.1817	= 4.726 g
	25 % ammonia solution, 2 mL (used to wash the product) x 0.9	= 1.8 g

$$E\text{-factor} = \frac{\textit{mass (waste)}}{\textit{mass (product)}}$$

$$= \frac{0.195 \text{ g (sodium azide)} + 0.065 \text{ g (copper catalyst)} + 4.726 \text{ g (reaction medium)} + 9 \text{ g (ammonia solution used to wash the product)}}{0.931 \text{ g} + 0.921 \text{ g} + 0.921 \text{ g} + 0.931 \text{ g} + 0.921 \text{ g (product)}}$$

= 13.986 g / 4.625 g

= **3.02 kg waste / 1 kg of product**

Method B: 1.5 mol% of catalyst loading, 70 °C, 12 h in ChCl/glycerol (1:2),

Substrate:	acetobromo-α-D-glucose (2 mmol)	= 0.822 g
	Phenyl acetylene (2 mmol)	= 0.204 g
	Sodium azide (5 mmol)	= 0.325 g
Catalyst:	Cu(I) iodide complex **1** (1.5 mol %)	= 0.013 g
Solvent:	ChCl/glycerol (4 mL) x 1.194	= 4.776 g
	25 % ammonia solution, 2 mL (used to wash the product) x 0.9	= 1.8 g

$$E\text{-factor} = \frac{\textit{mass (waste)}}{\textit{mass (product)}}$$

$$= \frac{0.195 \text{ g (sodium azide)} + 0.065 \text{ g (copper catalyst)} + 4.776 \text{ g (reaction medium)} + 9 \text{ g (ammonia solution used to wash the product)}}{0.893 \text{ g} + 0.903 \text{ g} + 0.892 \text{ g} + 0.897 \text{ g} + 0.905 \text{ g (product)}}$$

= 14.036 g / 4.49 g

= **3.12 kg waste / 1 kg of product**

E-factor for a single reaction run in in Method A and Method B: Assuming that the reaction medium can be infinitely recycled.

Method A: 1.5 mol% of catalyst loading, 70 °C, 12 h in TBAB/glycerol (1:4)

$$E\text{-factor} = \frac{mass\ (waste)}{mass\ (product)}$$

$$= \frac{0.195\text{ g (sodium azide)} + 0.013\text{ g (copper catalyst)} + 0.054\text{ (solvent TBAB/glycerol)} + 1.8\text{ g (ammonia solution used to wash the product)}}{0.931\text{ g (product)}}$$

$$= 2.062\text{ g} / 0.931\text{ g}$$

$$= \mathbf{2.21\ kg\ waste\ /\ 1\ kg\ of\ product}$$

Method B: 1.5 mol% of catalyst loading, 70 °C, 12 h in ChCl/glycerol (1:2),

$$E\text{-factor} = \frac{mass\ (waste)}{mass\ (product)}$$

$$= \frac{0.195\text{ g (sodium azide)} + 0.013\text{ g (copper catalyst)} + 0.126\text{ (solvent ChCl/glycerol)} + 1.8\text{ g (ammonia solution used to wash the product)}}{0.893\text{ g (product)}}$$

$$= 2.134\text{ g} / 0.893\text{ g}$$

$$= \mathbf{2.38\ kg\ waste\ /\ 1\ kg\ of\ product}$$

Synthesis and isolation of acetoazido-*β*-D-glucose from acetobromo-*α*-D-glucose followed by the reaction with phenyl acetylene to form β-D-Glucopyranosyltriazole:

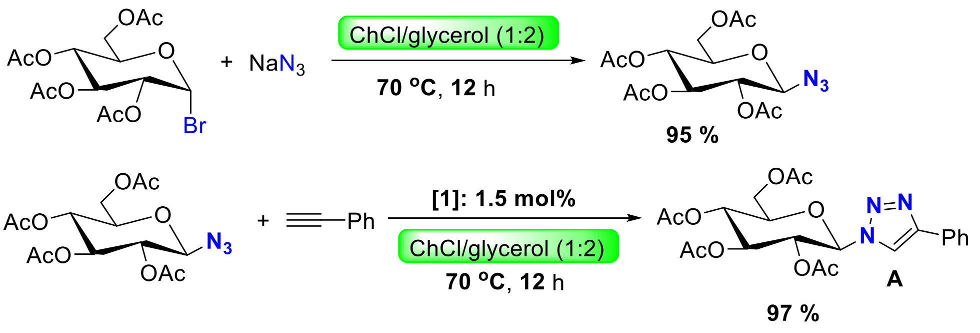

In the first step, acetobromo-*α*-D-glucose (0.206 g, 0.5 mmol) and NaN_3 (0.040 g, 1.25 mmol) were weighed in a vial (10 mL). Thereafter, ChCl/Glycerol (1:2) or TBAB/glycerol (1:4) was added into the vial. The resultant mixture was heated at 70 °C for 12 h with stirring. Formation of solid crude was observed after the desire reaction time. The crude solid product was then filtered and washed with additional 2 mL of water. The crude was then dried under vacuum and white solid compound was isolated as pure form. The product (2R,3R,4S,5R,6R)-2-(acetoxymethyl)-6-azidotetrahydro-2H-pyran-3,4,5-triyl triacetate was dissolved in $CDCl_3$ and

^{1}H NMR spectrum was recorded. Yield: 176 mg, 95%). ^{1}H NMR (700 MHz, $CDCl_3$) δ 5.21 (t, *J* = 9.5 Hz, 1H), 5.09 (t, *J* = 9.8 Hz, 1H), 4.94 (t, *J* = 9.2 Hz, 1H), 4.64 (d, *J* = 8.9 Hz, 1H), 4.26 (dd, *J* = 12.5, 4.8 Hz, 1H), 4.16 (dd, *J* = 12.4, 2.0 Hz, 1H), 3.79 (ddd, *J* = 10.0, 4.7, 2.2 Hz, 1H), 2.09 (s, 3H), 2.07 (s, 3H), 2.02 (s, 3H), 2.00 (s, 3H). ^{13}C NMR (101 MHz,) δ 170.73, 170.24, 169.43, 169.33, 88.04, 74.15, 72.73, 70.77, 68.01, 61.78, 20.81, 20.66.

In the following step, 0.25 mmol of acetoazido-*β*-D-glucose is treated with 0.25 mmol of phenyl acetylene in presence of catalyst **1** (1.5 mol %) in ChCl/Glycerol or TBAB/glycerol for 12 h and the resultant mixture was heated at 70 °C with stirring. Formation of solid crude was observed after the desire reaction time. The crude solid product was then filtered and washed with additional 2 mL of 25 % ammonia solution. The compound was then dried under vacuum and white crude solid compound was isolated as pure form. The product was dissolved in $CDCl_3$ and ^{1}H NMR spectrum was recorded.

NMR data of the products:

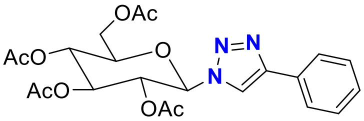

(2R,3R,4S,5R,6R)-2-(acetoxymethyl)-6-(4-phenyl-1H-1,2,3-triazol-1-yl)tetrahydro-2H-pyran-3,4,5-triyl triacetate (A): Isolated as white solid (46.5 mg, 98%). ^{1}H NMR (400 MHz, $CDCl_3$) δ 8.00 (s, 1H), 7.86 – 7.80 (m, 2H), 7.43 (t, *J* = 7.5 Hz, 2H), 7.36 (d, *J* = 7.4 Hz, 1H), 5.93 (d, *J* = 9.3 Hz, 1H), 5.52 (t, *J* = 9.4 Hz, 1H), 5.44 (t, *J* = 9.4 Hz, 1H), 5.27 (t, *J* = 9.7 Hz, 1H), 4.33 (dd, *J* = 12.6, 5.1 Hz, 1H), 4.16 (dd, *J* = 12.6, 2.0 Hz, 1H), 4.03 (ddd, *J* = 10.1, 5.0, 2.1 Hz, 1H), 2.08 (d, *J* = 3.9 Hz, 6H), 2.04 (s, 3H), 1.88 (s, 3H). ^{13}C NMR (101 MHz, $CDCl_3$) δ 170.65, 170.07, 169.53, 169.16, 148.65, 130.01, 129.02, 128.72, 126.05, 117.86, 85.94, 75.31, 72.87, 70.33, 67.88, 61.72, 20.82, 20.68, 20.66, 20.32.

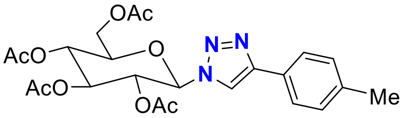

(2R,3R,4S,5R,6R)-2-(acetoxymethyl)-6-(4-(p-tolyl)-1H-1,2,3-triazol-1-yl)tetrahydro-2H-pyran-3,4,5-triyl triacetate (B_1): Isolated as off white solid (47 mg, 96%). ^{1}H NMR (700 MHz, $CDCl_3$) δ 7.96 (d, *J* = 2.8 Hz, 1H), 7.77 – 7.67 (m, 2H), 7.24 (d, *J* = 4.5 Hz, 2H), 5.93 (dd, *J* = 9.3, 2.8 Hz, 1H), 5.52 (td, *J* = 9.4, 2.8 Hz, 1H), 5.44 (dd, *J* = 9.3, 2.8 Hz, 1H), 5.27 (dd, *J* = 9.7, 2.7 Hz, 1H), 4.40 – 4.24 (m, 1H), 4.16 (d, *J* = 12.6 Hz, 1H), 4.03 (d, *J* = 4.8 Hz, 1H), 2.38 (d, *J* = 2.4 Hz, 3H), 2.08 (dd, *J* = 6.4, 3.0 Hz, 6H), 2.03 (d, *J* = 2.8 Hz, 3H), 1.88 (d, *J* = 2.8 Hz, 3H). ^{13}C NMR (176 MHz, $CDCl_3$) δ 170.66, 170.07, 169.53, 169.15, 148.72, 138.63, 129.68, 127.18, 125.95, 117.47, 85.90, 75.27, 72.91, 70.30, 67.88, 61.72, 21.43, 20.82, 20.67, 20.66, 20.31.

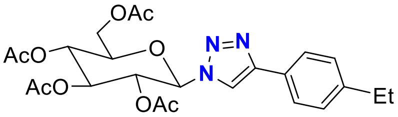

(2R,3R,4S,5R,6R)-2-(acetoxymethyl)-6-(4-(4-ethylphenyl)-1H-1,2,3-triazol-1-yl)tetrahydro-2H-pyran-3,4,5-triyl triacetate(B_2): Isolated as white solid (49 mg, 97%). ^{1}H NMR (400 MHz, $CDCl_3$) δ 7.96 (s, 1H), 7.76 (s, 1H), 7.74 (s, 1H), 7.27 (s, 1H), 7.25 (s, 1H), 5.93 (d, *J* = 9.3 Hz, 1H), 5.52 (t, *J* = 9.4 Hz, 1H), 5.44 (t, *J* = 9.4 Hz, 1H), 5.31 – 5.22 (m, 1H), 4.40 – 4.25 (m, 1H), 4.16 (dd, *J* = 12.6, 2.1 Hz, 1H), 4.03 (ddd, *J* = 10.1, 5.0, 2.1 Hz, 1H), 2.68 (q, *J* = 7.6 Hz, 2H), 2.09 (s, 3H), 2.07 (s, 3H), 2.03 (s, 3H), 1.87 (s, 3H), 1.25 (t, *J* = 7.6 Hz, 3H). ^{13}C NMR (101 MHz, $CDCl_3$) δ 170.66, 170.07, 169.53, 169.13, 148.74, 145.02, 128.51, 127.43, 126.05, 117.47 (s), 85.92, 75.29, 72.91, 70.31, 67.90, 61.73, 28.82, 20.83, 20.68, 20.65, 20.31, 15.64.

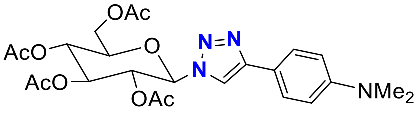

(2R,3R,4S,5R,6R)-2-(acetoxymethyl)-6-(4-(4-(dimethylamino)phenyl)-1H-1,2,3-triazol-1-yl)tetrahydro-2H-pyran-3,4,5-triyl triacetate(B_3): Isolated as white solid (50 mg, 97%).^{1}H NMR (400 MHz, $CDCl_3$) δ 7.84 (s, 1H), 7.72 – 7.67 (m, 2H), 6.78 – 6.74 (m, 1H), 5.91 (d, *J* = 9.4 Hz), 5.53 (t, *J* = 9.5 Hz, 2H), 5.42 (t, *J* = 9.4 Hz, 1H), 5.32 – 5.19 (m, 1H), 4.32 (dd, *J* = 12.6, 5.1 Hz, 1H), 4.15 (dd, *J* = 12.6, 2.1 Hz, 1H), 4.01 (ddd, *J* = 10.1, 5.0, 2.1 Hz, 1H), 2.99 (s, 3H), 2.08 (s, 3H), 2.07 (s, 3H), 2.03 (s, 3H), 1.87 (s, 3H).^{13}C NMR (101 MHz,$CDCl_3$) δ 170.67, 170.09, 169.53, 169.13, 150.80, 149.16, 127.00, 118.11, 116.11, 112.53, 85.85, 75.22, 73.03, 70.25, 67.93, 61.76, 40.56, 20.83, 20.68, 20.67, 20.33.

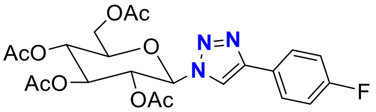

(2R,3R,4S,5R,6R)-2-(acetoxymethyl)-6-(4-(4-fluorophenyl)-1H-1,2,3-triazol-1-yl)tetrahydro-2H-pyran-3,4,5-triyl triacetate (B_4): Isolated as white solid (46.8 mg, 95%). ^{1}H NMR (400 MHz, $CDCl_3$) δ 8.01 (s, 1H), 7.71 (d, J = 8.4 Hz, 2H), 7.56 (d, J = 8.4 Hz, 2H), 5.93 (d, J = 9.0 Hz, 1H), 5.47 (dt, J = 24.7, 9.5 Hz, 2H), 5.26 (t, J = 9.6 Hz, 1H), 4.33 (dd, J = 12.7, 5.0 Hz, 1H), 4.15 (d, J = 12.6 Hz, 1H), 4.03 (dd, J = 10.1, 3.2 Hz, 1H), 2.08 (d, J = 3.4 Hz, 6H), 2.04 (s, 3H), 1.89 (s, 3H). ^{13}C NMR (101 MHz, $CDCl_3$) δ 170.65, 170.06, 169.53, 169.20, 147.61, 132.18, 128.93, 127.55, 122.68, 118.01, 85.95, 77.48, 77.16, 76.84, 75.32, 72.75, 70.31, 67.78, 61.67, 20.83, 20.68, 20.66, 20.33.

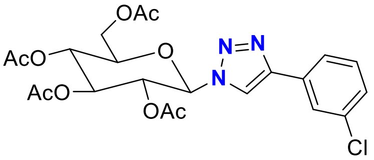

(2R,3R,4S,5R,6R)-2-(acetoxymethyl)-6-(4-(3-chlorophenyl)-1H-1,2,3-triazol-1-yl) tetrahydro-2H-pyran-3,4,5-triyl triacetate (B_5): Isolated as white solid (47.8 mg, 94%). ^{1}H NMR (400 MHz, $CDCl_3$) δ 8.03 (s, 1H), 7.83 (t, J = 1.6 Hz, 1H), 7.70 (dt, J = 7.4, 1.5 Hz, 1H), 7.32 (ddd, J = 11.0, 8.4, 4.9 Hz, 2H), 5.93 (d, J = 9.0 Hz, 1H), 5.47 (dt, J = 18.5, 9.5 Hz, 2H), 5.33 – 5.19 (m, 1H), 4.32 (dd, J = 12.7, 5.0 Hz, 1H), 4.15 (dd, J = 12.6, 2.0 Hz, 1H), 4.04 (ddd, J = 10.1, 5.0, 2.1 Hz, 1H), 2.07 (d, J = 3.6 Hz, 6H), 2.03 (s, 3H), 1.88 (s, 3H). ^{13}C NMR (101 MHz, $CDCl_3$) δ 170.60, 170.01, 169.49, 169.13, 147.30, 134.96, 131.75, 130.28, 128.66, 126.07, 124.08, 118.39, 85.95, 75.30, 72.73, 70.38, 67.81, 61.66, 20.78, 20.64, 20.61, 20.27.

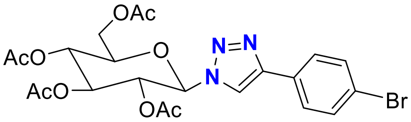

(2R,3R,4S,5R,6R)-2-(acetoxymethyl)-6-(4-(4-bromophenyl)-1H-1,2,3-triazol-1-yl)tetrahydro-2H-pyran-3,4,5-triyl triacetate (B_6): Isolated as white solid (51.43 mg, 93%). ^{1}H NMR (400 MHz, $CDCl_3$) δ 8.01 (s, 1H), 7.71 (d, J = 8.4 Hz, 2H), 7.56 (d, J = 8.4 Hz, 2H), 5.93 (d, J = 9.0 Hz, 1H), 5.47 (dt, J = 25.0, 9.5 Hz, 2H), 5.26 (t, J = 9.6 Hz, 1H), 4.33 (dd, J = 12.7, 5.0 Hz, 1H), 4.15 (d, J = 12.6 Hz, 1H), 4.03 (dd, J = 10.1, 3.3 Hz, 1H), 2.08 (d, J = 3.7 Hz, 6H), 2.04 (s, 3H), 1.89 (s, 3H). ^{13}C NMR (101 MHz, $CDCl_3$) δ 170.65, 170.06, 169.54, 169.20, 132.19, 128.97, 127.54, 122.69, 86.02, 75.34, 72.75, 70.31, 67.78, 61.67, 20.85, 20.70, 20.67, 20.35.

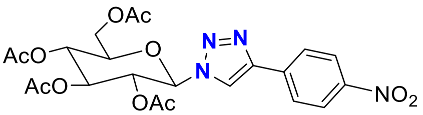

(2R,3R,4S,5R,6R)-2-(acetoxymethyl)-6-(4-(4-nitrophenyl)-1H-1,2,3-triazol-1-yl)tetrahydro-2H-pyran-3,4,5-triyl triacetate (B_7): Isolated as yellow solid (46.8 mg, 90%). ^{1}H NMR (700 MHz, $CDCl_3$) δ 8.31 (d, J = 8.5 Hz, 2H), 8.16 (s, 1H), 8.02 (d, J = 8.5 Hz, 2H), 5.95 (d, J = 8.8 Hz, 1H), 5.48 (dq, J = 18.6, 9.5 Hz, 2H), 5.27 (t, J = 9.5 Hz, 1H), 4.35 (dd, J = 12.7, 5.0 Hz, 1H), 4.17 (d, J = 12.7 Hz, 1H), 4.05 (dd, J = 10.1, 4.9 Hz, 1H), 2.09 (d, J = 5.1 Hz, 6H), 2.05 (s, 3H), 1.90 (s, 3H). ^{13}C NMR (101 MHz, $CDCl_3$) δ 170.60, 170.01, 169.52, 169.25, 147.80, 146.48, 136.23, 126.60, 124.48, 119.54, 86.13, 75.53, 72.64, 70.49, 67.81, 61.67, 20.83, 20.68, 20.66, 20.33.

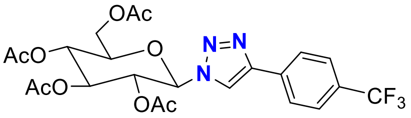

(2R,3R,4S,5R,6R)-2-(acetoxymethyl)-6-(4-(4-(trifluoromethyl)phenyl)-1H-1,2,3-triazol-1-yl)tetrahydro-2H-pyran-3,4,5-triyl triacetate(B_8): Isolated as off white solid (50 mg, 92%). ^1H NMR (400 MHz, $CDCl_3$) δ 8.10 (s, 1H), 7.96 (d, *J* = 8.1 Hz, 2H), 7.69 (d, *J* = 8.2 Hz, 2H), 5.95 (d, *J* = 9.0 Hz, 1H), 5.48 (dt, *J* = 18.4, 9.5 Hz, 2H), 5.32 – 5.22 (m, 1H), 4.34 (dd, *J* = 12.7, 5.1 Hz, 1H), 4.16 (dd, *J* = 12.6, 2.0 Hz, 1H), 4.05 (ddd, *J* = 10.1, 5.0, 2.0 Hz, 1H), 2.09 (s, 3H), 2.08 (s, 3H), 2.04 (s, 3H), 1.89 (s, 3H). ^{13}C NMR (101 MHz, $CDCl_3$) δ 170.64, 170.04, 169.54, 169.22, 147.26, 133.44, 130.75, 130.42, 126.22, 126.06, 126.02, 125.50, 122.80, 118.80, 86.03, 75.42, 72.73, 70.42, 67.83, 61.69, 20.82, 20.67, 20.64, 20.31.

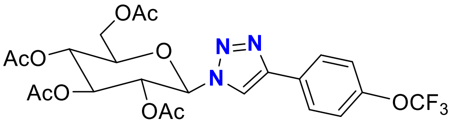

(2R,3R,4S,5R,6R)-2-(acetoxymethyl)-6-(4-(4-(trifluoromethoxy)phenyl)-1H-1,2,3-triazol-1-yl)tetrahydro-2H-pyran-3,4,5-triyl triacetate (B_9): Isolated as white solid (52.55 mg, 94%). ^1H NMR (700 MHz, $CDCl_3$) δ 8.01 (s, 1H), 7.86 (d, *J* = 8.4 Hz, 2H), 7.28 (d, *J* = 8.2 Hz, 2H), 5.93 (d, *J* = 9.3 Hz, 1H), 5.50 (t, *J* = 9.4 Hz, 1H), 5.45 (t, *J* = 9.4 Hz, 1H), 5.27 (t, *J* = 9.7 Hz, 1H), 4.33 (dd, *J* = 12.7, 4.9 Hz, 1H), 4.16 (d, *J* = 12.5 Hz, 1H), 4.04 (dd, *J* = 9.5, 4.0 Hz, 1H), 2.08 (d, *J* = 5.6 Hz, 6H), 2.04 (s, 3H), 1.89 (s, 3H). ^{13}C NMR (101 MHz, $CDCl_3$) δ 170.68, 170.08, 169.56, 169.22, 149.36, 147.37, 128.74, 127.46, 121.55, 118.10, 85.97, 75.33, 72.71, 70.31, 67.77, 61.66, 20.85, 20.70, 20.67, 20.33. HRMS (ESI-TOF) m/z: $[M + H]^+$ calcd for $C_{23}H_{25}F_3N_3O_{10}$ 560.1492; Found 560.1494.

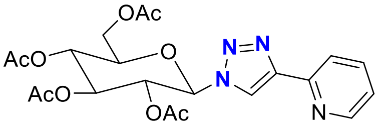

(2R,3R,4S,5R,6R)-2-(acetoxymethyl)-6-(4-(pyridin-2-yl)-1H-1,2,3-triazol-1-yl)tetrahydro-2H-pyran-3,4,5-triyl triacetate(B_{10}): Isolated as brown solid (45 mg, 95%). ^1H NMR (700 MHz, $CDCl_3$) δ 8.60 (s, 1H), 8.40 (s, 1H), 8.14 (d, *J* = 7.3 Hz, 1H), 7.77 (d, *J* = 6.8 Hz, 1H), 7.25 (s, 1H), 5.92 (d, *J* = 8.9 Hz, 1H), 5.47 (dt, *J* = 31.7, 8.3 Hz, 2H), 5.33 – 5.23 (m, 2H), 4.31 (dd, *J* = 12.5, 4.7 Hz, 1H), 4.16 (d, *J* = 12.5 Hz, 1H), 4.02 (d, *J* = 5.3 Hz, 1H), 2.08 (d, *J* = 12.7 Hz, 6H), 2.04 (s, 3H), 1.89 (s, 3H). ^{13}C NMR (101 MHz, $CDCl_3$) δ 170.61, 170.06, 169.41, 168.93, 149.63, 149.05, 136.97, 123.27, 120.68, 120.47, 85.93, 75.18, 72.75, 70.56, 67.72, 61.58, 20.74, 20.61, 20.60, 20.26.

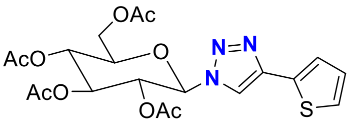

(2R,3R,4S,5R,6R)-2-(acetoxymethyl)-6-(4-(thiophen-2-yl)-1H-1,2,3-triazol-1-yl)tetrahydro-2H-pyran-3,4,5-triyl triacetate(B_{11}): Isolated as off white solid (45 mg, 94%). ^1H NMR (400 MHz, $CDCl_3$) δ 7.91 (s, 1H), 7.41 (dd, *J* = 3.6, 1.0 Hz, 1H), 7.32 (dd, *J* = 5.0, 1.0 Hz, 1H), 7.08 (dd, *J* = 5.0, 3.6 Hz, 1H), 5.91 (d, *J* = 9.1 Hz, 1H), 5.46 (dt, *J* = 25.3, 9.5 Hz, 2H), 5.26 (t, *J* = 9.6 Hz, 1H), 4.33 (dd, *J* = 12.6, 5.1 Hz, 1H), 4.16 (dd, *J* = 12.6, 1.9 Hz, 1H), 4.02 (ddd, *J* = 10.0, 5.0, 2.0 Hz, 1H), 2.08 (d, *J* = 6.0 Hz, 6H), 2.03 (s, 3H), 1.89 (s, 3H). ^{13}C NMR (101 MHz, $CDCl_3$) δ 170.65, 170.06, 169.51, 169.15, 143.77, 132.22, 127.82, 125.71, 124.96, 117.25, 85.98, 75.35, 72.83, 70.34, 67.83, 61.70, 20.83, 20.68, 20.65, 20.33.

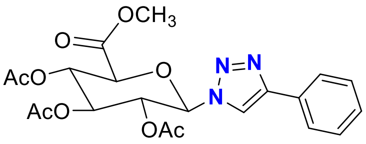

(2S,3S,4S,5R,6R)-2-(methoxycarbonyl)-6-(4-phenyl-1H-1,2,3-triazol-1-yl)tetrahydro-2H-pyran-3,4,5-triyl triacetate(C_1): Isolated as white solid (44 mg, 95%).^{1}H NMR (400 MHz, DMSO) δ 9.05 (s, 1H), 7.84 (d, *J* = 7.3 Hz, 2H), 7.48 (t, *J* = 7.6 Hz, 2H), 7.38 (d, *J* = 7.4 Hz, 1H), 6.47 (d, *J* = 9.1 Hz, 1H), 5.77 (t, *J* = 9.3 Hz, 1H), 5.68 (t, *J* = 9.4 Hz, 1H), 5.24 (t, *J* = 9.8 Hz, 1H), 4.87 (d, *J* = 10.0 Hz, 1H), 3.64 (s, 3H), 2.04 (s, 3H), 2.00 (s, 3H), 1.81 (s, 3H). ^{13}C NMR (101 MHz,) δ 169.52, 169.34, 168.53, 166.58, 147.02, 129.96, 129.04, 128.36, 125.22, 120.57, 83.84, 72.88, 71.42, 69.93, 68.47, 52.68, 23.05, 20.26, 20.22, 19.89.

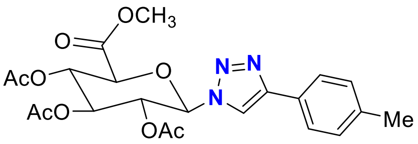

(2S,3S,4S,5R,6R)-2-(methoxycarbonyl)-6-(4-(p-tolyl)-1H-1,2,3-triazol-1-yl)tetrahydro-2H-pyran-3,4,5-triyl triacetate(C_2): Isolated as off white solid (46.5 mg, 98%). ^{1}H NMR (400 MHz, $CDCl_3$) δ 7.80 (s, 1H), 7.75 (d, *J* = 8.0 Hz, 2H), 7.23 (d, *J* = 7.9 Hz, 2H), 5.16 (t, *J* = 9.5 Hz, 1H), 5.11 – 4.99 (m, 2H), 4.68 (dd, *J* = 11.3, 3.5 Hz, 1H), 4.58 – 4.48 (m, 1H), 4.45 (d, *J* = 7.9 Hz, 1H), 4.31 – 4.23 (m, 2H), 4.12 (d, *J* = 12.4 Hz, 1H), 3.94 – 3.86 (m, 1H), 3.69 (dd, *J* = 9.9, 2.4 Hz, 1H), 2.37 (s, 3H), 2.08 (s, 3H), 2.02 (s, 3H), 1.98 (s, 3H), 1.71 (s, 3H).^{13}C NMR (101 MHz, DMSO) δ 169.50, 169.31, 168.50, 166.57, 147.06, 137.71, 129.55, 127.19, 125.15, 120.09, 83.80, 72.85, 71.44, 69.90, 68.46, 52.65, 20.83, 20.25, 20.21, 19.89. HRMS (ESI-TOF) m/z: $[M + H]^+$ calcd for $C_{22}H_{26}N_3O_9$ 476.1669; Found 476.1689.

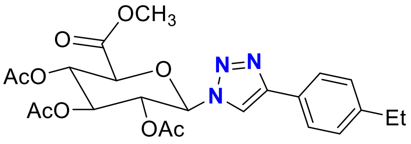

(2R,3R,4S,5S,6S)-2-(4-(4-ethylphenyl)-1H-1,2,3-triazol-1-yl)-6-(methoxycarbonyl)tetrahydro-2H-pyran-3,4,5-triyl triacetate(C_3): Isolated as white solid (47.4 mg, 97%). NMR (400 MHz, $CDCl_3$) δ 8.00 (s, 1H), 7.73 (d, *J* = 8.0 Hz, 2H), 7.25 (s, 2H), 5.95 (d, *J* = 8.9 Hz, 1H), 5.50 (dd, *J* = 11.8, 8.9 Hz, 2H), 5.38 (s,1H), 4.32 (d, *J* = 9.8 Hz, 1H), 3.74 (s, 3H), 2.66 (d, *J* = 7.6 Hz, 2H), 2.05 (d, *J* = 9.4 Hz, 6H), 1.86 (s, 3H), 1.24 (t, *J* = 7.6 Hz, 3H). ^{13}C NMR (101 MHz, $CDCl_3$) δ 169.91, 169.49, 169.06, 166.36, 148.85, 145.05, 128.53, 127.38, 126.06, 117.61, 85.64, 75.10, 72.16, 70.05, 69.18, 53.31, 28.84, 20.66, 20.60, 20.29, 15.64. HRMS(ESI-TOF)m/z: $[M+H]^+$ calcd for $C_{23}H_{28}N_3O_9$ 490.1826; Found 490.1811

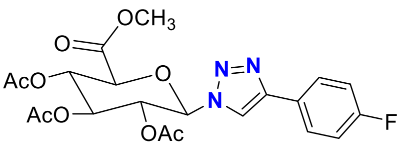

(2R,3R,4S,5S,6S)-2-(4-(4-fluorophenyl)-1H-1,2,3-triazol-1-yl)-6-(methoxycarbonyl)tetrahydro-2H-pyran-3,4,5-triyl triacetate(C_4): Isolated as of white solid (45 mg, 94%).^{1}H NMR (400 MHz, $CDCl_3$) δ 8.03 (s, 1H), 7.84 – 7.76 (m, 2H), 7.12 (dd, *J* = 12.0, 5.4 Hz, 2H), 6.01 – 5.93 (m, 1H), 5.56 – 5.46 (m, 2H), 5.39 (t, *J* = 9.5 Hz, 1H), 4.35 (d, *J* = 9.8 Hz, 1H), 3.75 (s, 3H), 2.07 (s, 3H), 2.05 (s, 3H), 1.89 (s, 3H).^{13}C NMR (101 MHz, $CDCl_3$) δ 169.87, 169.48, 169.10, 166.34, 161.83, 147.87, 127.85, 126.19, 117.81, 116.15,

115.94, 85.67, 75.07, 72.06, 70.11, 69.15, 53.32, 20.65, 20.58, 20.30. HRMS (ESI-TOF) m/z: $[M + H]^+$ calcd for $C_{21}H_{23}N_3O_9$ 480.1418; Found 480.1389.

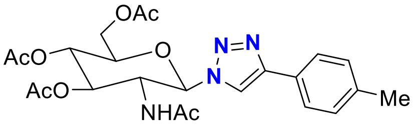

(2R,3S,6S)-5-acetamido-2-(acetoxymethyl)-6-(4-(p-tolyl)-1H-1,2,3-triazol-1-yl)tetrahydro-2H-pyran-3,4-diyl diacetate(D_1): Isolated as white solid (48.8 mg, 98%). ^{1}H NMR (400 MHz, DMSO) δ 8.79 (s, 1H), 8.13 (d, *J* = 9.2 Hz, 1H), 7.72 (d, *J* = 8.1 Hz, 2H), 7.28 (d, *J* = 8.0 Hz, 2H), 6.13 (d, *J* = 9.9 Hz, 1H), 5.39 (t, *J* = 9.9 Hz, 1H), 5.11 (t, *J* = 9.8 Hz, 1H), 4.65 (d, *J* = 9.7 Hz, 1H), 4.28 (ddd, *J* = 10.1, 5.0, 2.2 Hz, 1H), 4.17 (dd, *J* = 12.5, 5.1 Hz, 1H), 4.08 (dd, *J* = 12.4, 2.0 Hz, 1H), 2.33 (s, 3H), 2.02 (s, 3H), 2.00 (s, 3H), 1.96 (s, 3H), 1.59 (s, 3H).^{13}C NMR (101 MHz,DMSO) δ 169.50, 169.31, 168.50, 166.57, 147.06, 137.71, 129.55, 127.19, 125.15, 120.09, 83.80, 72.85, 71.44, 69.90, 68.46, 52.65, 20.83, 20.25, 20.21, 19.89. HRMS (ESI-TOF) m/z: $[M + H]^+$ calcd for $C_{23}H_{29}N_4O_8$ 489.1985; Found 489.1937.

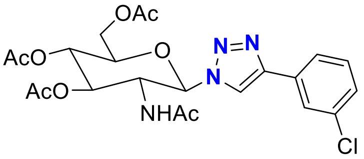

(2R,3S,6S)-5-acetamido-2-(acetoxymethyl)-6-(4-(3-chlorophenyl)-1H-1,2,3-triazol-1-yl)tetrahydro-2H-pyran-3,4-diyl diacetate(D_2): Isolated as white solid (47.3 mg, 93%). ^{1}H NMR (400 MHz, DMSO) δ 9.01 (s, 1H), 8.15 (d, *J* = 9.1 Hz, 1H), 7.90 (s, 1H), 7.82 (d, *J* = 7.9 Hz, 1H), 7.52 (t, *J* = 7.9 Hz, 1H), 7.44 (d, *J* = 8.0 Hz, 1H), 6.16 (d, *J* = 9.9 Hz, 1H), 5.38 (d, *J* = 9.9 Hz, 1H), 5.10 (t, *J* = 9.8 Hz, 1H), 4.63 (d, *J* = 9.8 Hz, 1H), 4.32 (d, *J* = 7.3 Hz, 1H), 4.19 (dd, *J* = 12.6, 5.0 Hz, 1H), 4.09 (d, *J* = 10.7 Hz, 1H), 2.03 (s, *J* = 7.1 Hz, 3H), 2.02 (s, 3H), 1.97 (s, 3H), 1.60 (s, 3H). ^{13}C NMR (101 MHz, DMSO) δ 170.03, 169.58, 169.50, 169.37, 145.49, 131.99, 129.48, 127.17, 121.18, 120.65, 84.95, 73.38, 72.25, 68.02, 61.74, 52.35, 22.31, 20.52, 20.42, 20.28. HRMS (ESI-TOF) m/z: $[M + H]^+$ calcd for $C_{22}H_{26}ClN_4O_8$ 509.1439; Found 509.1446.

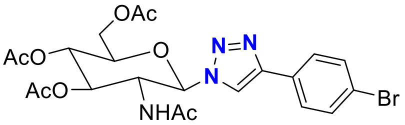

(2R,3S,6S)-5-acetamido-2-(acetoxymethyl)-6-(4-(3-bromophenyl)-1H-1,2,3-triazol-1-yl)tetrahydro-2H-pyran-3,4-diyl diacetate(D_3): Isolated as white solid (51.4 mg, 92%).^{1}H NMR (400 MHz, DMSO) δ 8.92 (s, 1H), 8.11 (d, *J* = 9.2 Hz, 1H), 7.81 – 7.77 (m, 2H), 7.69 – 7.66 (m, 2H), 6.15 (d, *J* = 9.9 Hz, 1H), 5.39 (t, *J* = 9.9 Hz, 1H), 5.11 (t, *J* = 9.8 Hz, 1H), 4.64 (q, *J* = 9.8 Hz, 1H), 4.29 (ddd, *J* = 10.1, 5.0, 2.2 Hz, 1H), 4.18 (dd, *J* = 12.5, 5.0 Hz, 1H), 4.09 (dd, *J* = 12.5, 2.1 Hz, 1H), 2.02 (s, 3H), 2.01 (s, 3H), 1.96 (s, 3H), 1.59 (s, 3H). ^{13}C NMR (101 MHz, DMSO) δ 170.03, 169.58, 169.50, 169.37, 145.49, 131.99, 129.48, 127.17, 121.18, 120.65, 84.95, 73.38, 72.25, 68.02, 61.74, 52.35, 22.31, 20.52, 20.42, 20.28.

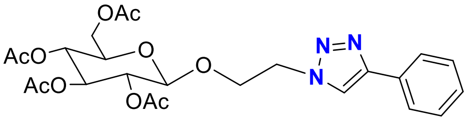

(2R,3R,4S,5R,6R)-2-(acetoxymethyl)-6-(2-(4-phenyl-1H-1,2,3-triazol-1-yl)ethoxy)tetrahydro-2H-pyran-3,4,5-triyl triacetate(E_1): Isolated as white solid (50.9 mg, 98%).^{1}H NMR (400 MHz, $CDCl_3$) δ 7.88 (s, 1H), 7.86 (s, 2H), 7.42 (t, *J* = 7.5 Hz, 2H), 7.32 (t, *J* = 7.3 Hz, 1H), 5.16 (t, *J* = 9.5 Hz, 1H), 5.12 – 4.97 (m, 2H), 4.70 (d, *J* = 14.7 Hz, 1H), 4.54 (dd, *J* = 12.1, 9.2 Hz, 1H), 4.46 (d, *J* = 7.9 Hz, 1H), 4.33 – 4.21 (m, 2H), 4.12 (d, *J* = 11.0

Hz, 1H), 3.91 (t, J = 8.6 Hz, 1H), 3.75 – 3.64 (m, 1H), 2.07 (s, 3H), 2.01 (s, 3H), 1.97 (s, 3H), 1.71 (s, 3H). ^{13}C NMR (101 MHz, $CDCl_3$) δ 170.76, 170.22, 169.65, 169.57, 130.54, 128.96, 128.30, 125.78, 121.67, 100.62, 72.49, 72.04, 70.96, 68.25, 68.00, 61.76, 50.19, 20.86, 20.71, 20.68, 20.49.

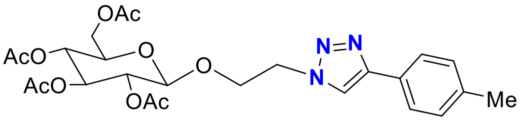

(2R,3R,4S,5R,6R)-2-(acetoxymethyl)-6-(2-(4-(p-tolyl)-1H-1,2,3-triazol-1-yl)ethoxy)tetrahydro-2H-pyran-3,4,5-triyl triacetate(E_2) : Isolated as off white solid (50.6 mg, 95%).^{1}H NMR (400 MHz, $CDCl_3$) δ 7.80 (s, 1H), 7.75 (d, J = 8.0 Hz, 2H), 7.23 (d, J = 7.9 Hz, 2H), 5.16 (t, J = 9.5 Hz, 1H), 5.11 – 4.99 (m, 2H), 4.68 (dd, J = 11.3, 3.5 Hz, 1H), 4.58 – 4.48 (m, 1H), 4.45 (d, J = 7.9 Hz, 1H), 4.31 – 4.23 (m, 2H), 4.12 (d, J = 12.4 Hz, 1H), 3.94 – 3.86 (m, 1H), 3.69 (dd, J = 9.9, 2.4 Hz, 1H), 2.37 (s, 3H), 2.08 (s, 3H), 2.02 (s, 3H), 1.98 (s, 3H), 1.71 (s, 3H). ^{13}C NMR (101 MHz, $CDCl_3$) δ 170.76, 170.23, 169.65, 169.58, 130.59, 128.96, 128.28, 125.77, 121.60, 100.63, 72.50, 72.04, 70.97, 68.25, 68.01, 61.76, 50.17, 20.86, 20.71, 20.68, 20.49.

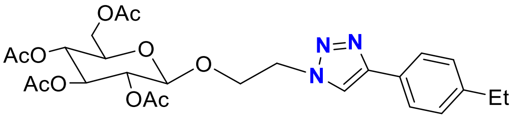

(2R,3R,4S,5R,6R)-2-(acetoxymethyl)-6-(2-(4-(4-ethylphenyl)-1H-1,2,3-triazol-1-yl)ethoxy)tetrahydro-2H-pyran-3,4,5-triyl triacetate(E_3) : Isolated as white solid (52.5 mg, 96%). ^{1}H NMR (400 MHz, $CDCl_3$) δ 7.80 (s, 1H), 7.76 (d, J = 8.0 Hz, 2H), 7.24 (d, J = 8.0 Hz, 2H), 5.15 (t, J = 9.5 Hz, 1H), 5.08 – 4.98 (m, 2H), 4.71 – 4.60 (m, 1H), 4.55 – 4.48 (m, 1H), 4.45 (d, J = 7.9 Hz, 1H), 4.29 – 4.20 (m, 2H), 4.11 (dd, J = 12.3, 2.0 Hz, 1H), 3.90 (td, J = 9.7, 2.7 Hz, 1H), 3.68 (ddd, J = 9.8, 4.6, 2.3 Hz, 1H), 2.65 (q, J = 7.6 Hz, 2H), 2.05 (s, 3H), 2.00 (s, 3H), 1.96 (s, 3H), 1.71 (s, 3H), 1.23 (t, J = 7.6 Hz, 3H). ^{13}C NMR (101 MHz, $CDCl_3$) δ 170.68, 170.14, 169.58, 169.51, 147.82, 144.43, 128.39, 128.01, 125.75, 121.16, 100.62, 72.54, 72.03, 70.99, 68.31, 68.00, 61.78, 50.11, 28.73, 20.78, 20.64, 20.62, 20.46, 15.56.

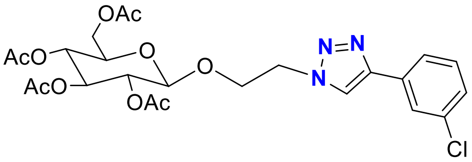

(2R,3R,4S,5R,6R)-2-(acetoxymethyl)-6-(2-(4-(3-chlorophenyl)-1H-1,2,3-triazol-1-yl)ethoxy)tetrahydro-2H-pyran-3,4,5-triyl triacetate (E_4)): Isolated as white solid (51.5 mg, 93%).^{1}H NMR (400 MHz, $CDCl_3$) δ 7.85 (s, 1H), 7.75 (d, J = 8.3 Hz, 2H), 7.54 (d, J = 8.3 Hz, 2H), 5.16 (t, J = 9.5 Hz, 1H), 5.11 – 4.96 (m, 2H), 4.68 (d, J = 14.5 Hz, 1H), 4.59 – 4.48 (m, 1H), 4.46 (d, J = 7.8 Hz, 1H), 4.25 (dd, J = 12.1, 4.6 Hz, 2H), 4.12 (d, J = 11.2 Hz, 1H), 3.90 (t, J = 8.4 Hz, 1H), 3.69 (d, J = 7.6 Hz, 1H), 2.06 (s, 3H), 2.01 (s, 3H), 1.98 (s, 3H), 1.73 (s, 3H). ^{13}C NMR (101 MHz, $CDCl_3$) δ 170.58, 170.07, 169.49, 169.43, 146.60, 131.99, 129.52, 127.22, 122.02, 121.64, 100.50, 72.37, 71.99, 70.94, 68.21, 67.76, 61.69, 50.13, 20.71, 20.56, 20.54, 20.43. HRMS (ESI-TOF) m/z: $[M + H]^+$ calcd for $C_{24}H_{29}ClN_3O_{10}$ 554.1389; Found 554.1542.

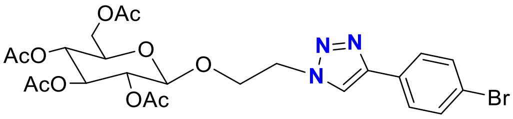

(2R,3R,4S,5R,6R)-2-(acetoxymethyl)-6-(2-(4-(4-bromophenyl)-1H-1,2,3-triazol-1-yl)ethoxy)tetrahydro-2H-pyran-3,4,5-triyl triacetate(E_5): Isolated as white solid (54.4 mg, 91%).^{1}H NMR (400 MHz, $CDCl_3$) δ 7.85 (s, 1H), 7.75 (d, J = 8.3 Hz, 2H), 7.54 (d, J = 8.3 Hz, 2H), 5.16 (t, J = 9.5 Hz, 1H), 5.11 – 4.96 (m, 2H), 4.68 (d, J = 14.5 Hz, 1H), 4.59 – 4.48 (m,

1H), 4.46 (d, *J* = 7.8 Hz, 1H), 4.25 (dd, *J* = 12.1, 4.6 Hz, 2H), 4.12 (d, *J* = 11.2 Hz, 1H), 3.90 (t, *J* = 8.4 Hz, 1H), 3.69 (d, *J* = 7.6 Hz, 1H), 2.06 (s, 3H), 2.01 (s, 3H), 1.98 (s, 3H), 1.73 (s, 3H). ^{13}C NMR (101 MHz, $CDCl_3$) δ 170.58, 170.07, 169.49, 169.43, 146.60, 131.99, 129.52, 127.22, 122.02, 121.64, 100.50, 72.37, 71.99, 70.94, 68.21, 67.76, 61.69, 50.13, 20.71, 20.56, 20.54, 20.43. HRMS (ESI-TOF) m/z: $[M + H]^+$ calcd for $C_{24}H_{28}BrN_3O_{10}$ 598.1036; Found 598.1036.

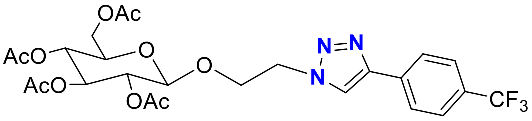

(2R,3R,4S,5R,6R)-2-(acetoxymethyl)-6-(2-(4-(4-(trifluoromethyl)phenyl)-1H-1,2,3-triazol-1-yl)ethoxy)tetrahydro-2H-pyran-3,4,5-triyl triacetate(E_6): Isolated as white solid (52.8 mg, 90%).^{1}H NMR (400 MHz, $CDCl_3$) δ 8.00 (d, *J* = 8.1 Hz, 2H), 7.95 (s, 1H), 7.68 (d, *J* = 8.2 Hz, 2H), 5.17 (t, *J* = 9.5 Hz, 1H), 5.10 – 4.98 (m, 2H), 4.75 – 4.64 (m, 1H), 4.60 – 4.51 (m, 1H), 4.47 (d, *J* = 7.9 Hz, 1H), 4.31 – 4.22 (m, 2H), 4.13 (dd, *J* = 12.3, 2.0 Hz, 1H), 3.92 (td, *J* = 10.0, 2.5 Hz, 1H), 3.70 (ddd, *J* = 9.7, 4.4, 2.1 Hz, 1H), 2.06 (s, 3H), 2.01 (s, 3H), 1.98 (s, 3H), 1.73 (s, 3H). ^{13}C NMR (101 MHz, $CDCl_3$) δ 170.69, 170.18, 169.59, 169.55, 146.38, 134.14, 130.25, 129.92, 125.97, 125.76, 122.51, 100.62, 72.47, 72.14, 71.09, 68.34, 67.79, 61.82, 50.33, 20.81, 20.67, 20.65, 20.54. HRMS (ESI-TOF) m/z: $[M + H]^+$ calcd for $C_{25}H_{29}F_3N_3O_{10}$ 588.1805; Found 588.2238.

NMR spectra of the products:

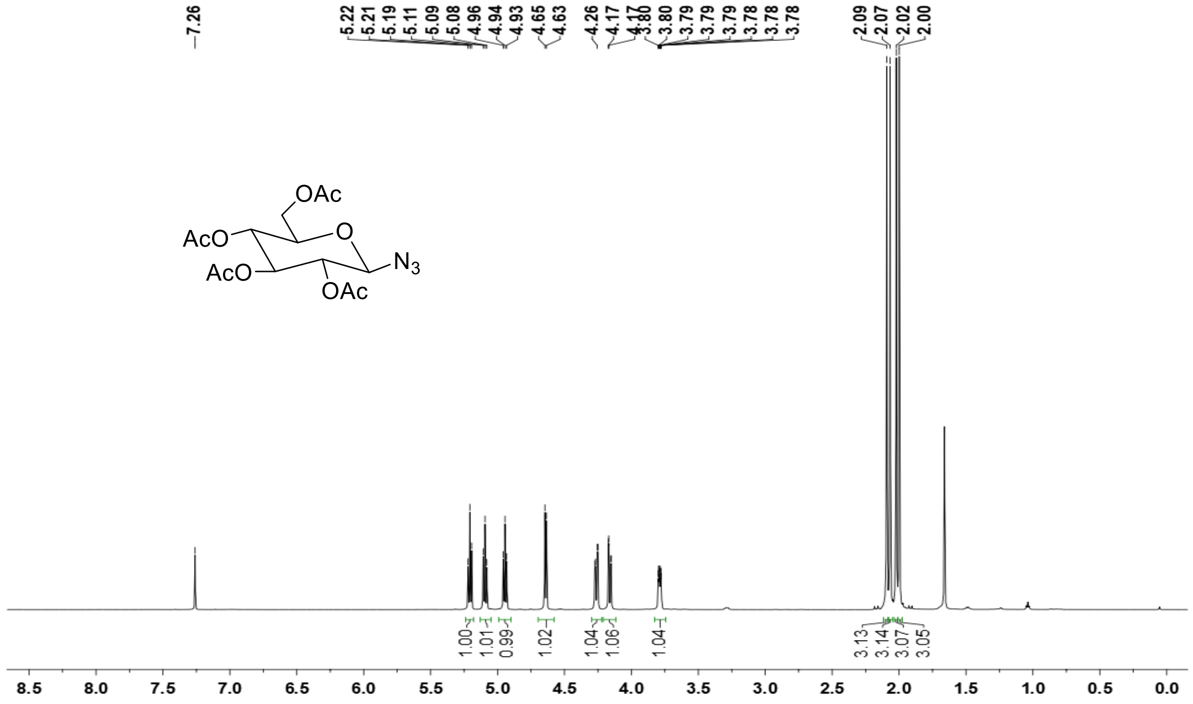

Figure S7. ^{1}H NMR of (2R,3R,4S,5R,6R)-2-(acetoxymethyl)-6-azidotetrahydro-2H-pyran-3,4,5-triyl triacetate in $CDCl_3$ at r.t.

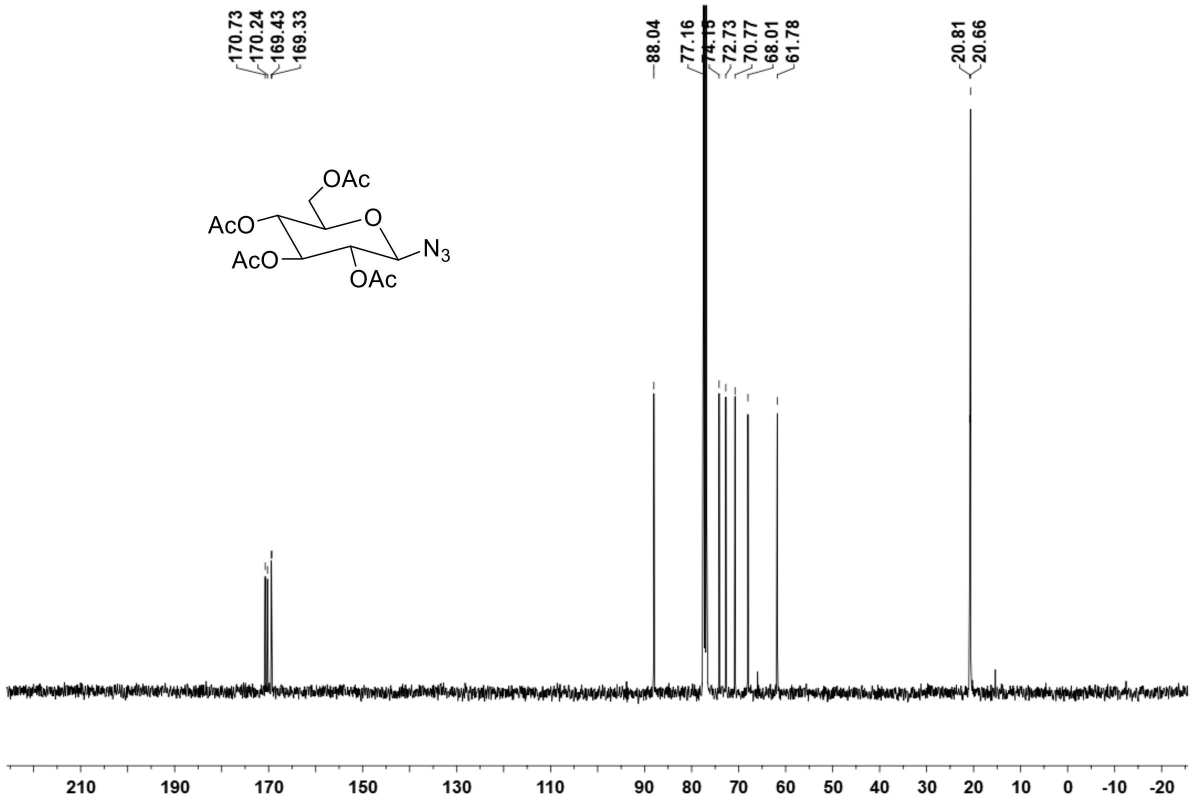

Figure S8. ^{13}C NMR of (2R,3R,4S,5R,6R)-2-(acetoxymethyl)-6-azidotetrahydro-2H-pyran-3,4,5-triyl triacetate in $CDCl_3$ at r.t.

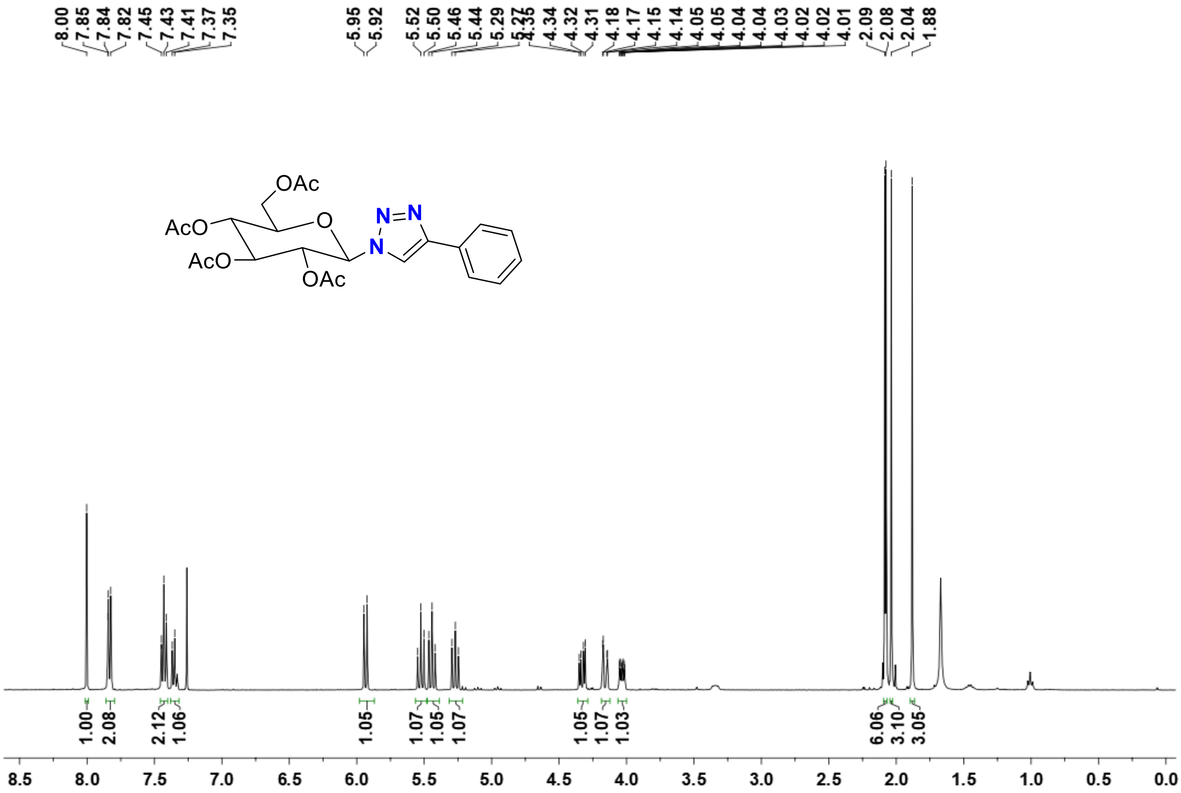

Figure S9. ^{1}H NMR (400 MHz) spectrum of (2R,3R,4S,5R,6R)-2-(acetoxymethyl)-6-(4-phenyl-1H-1,2,3-triazol-1-yl) tetrahydro-2H-pyran-3,4,5-triyl triacetate **(A)** $CDCl_3$ at r.t.

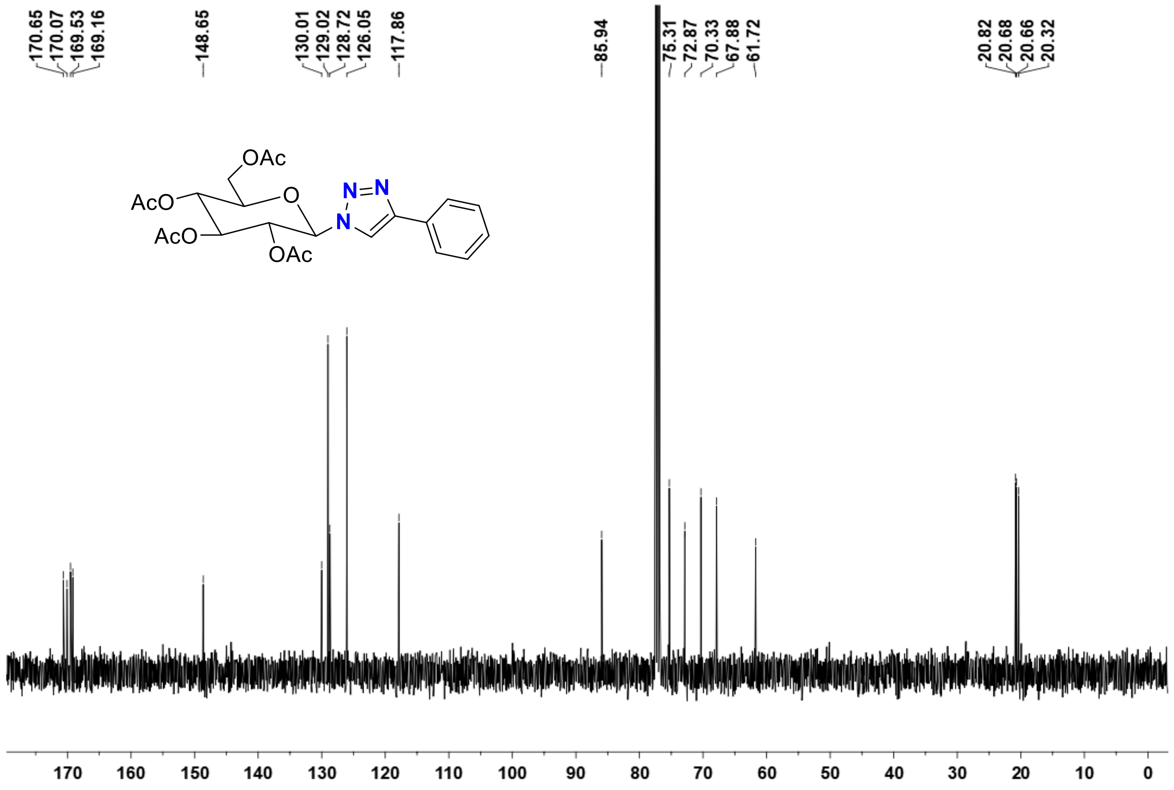

Figure S10. ^{13}C NMR (101 MHz) spectrum of (2R,3R,4S,5R,6R)-2-(acetoxymethyl)-6-(4-phenyl-1H-1,2,3-triazol-1-yl)tetrahydro-2H-pyran-3,4,5-triyl triacetate **(A)** in $CDCl_3$ at r.t.

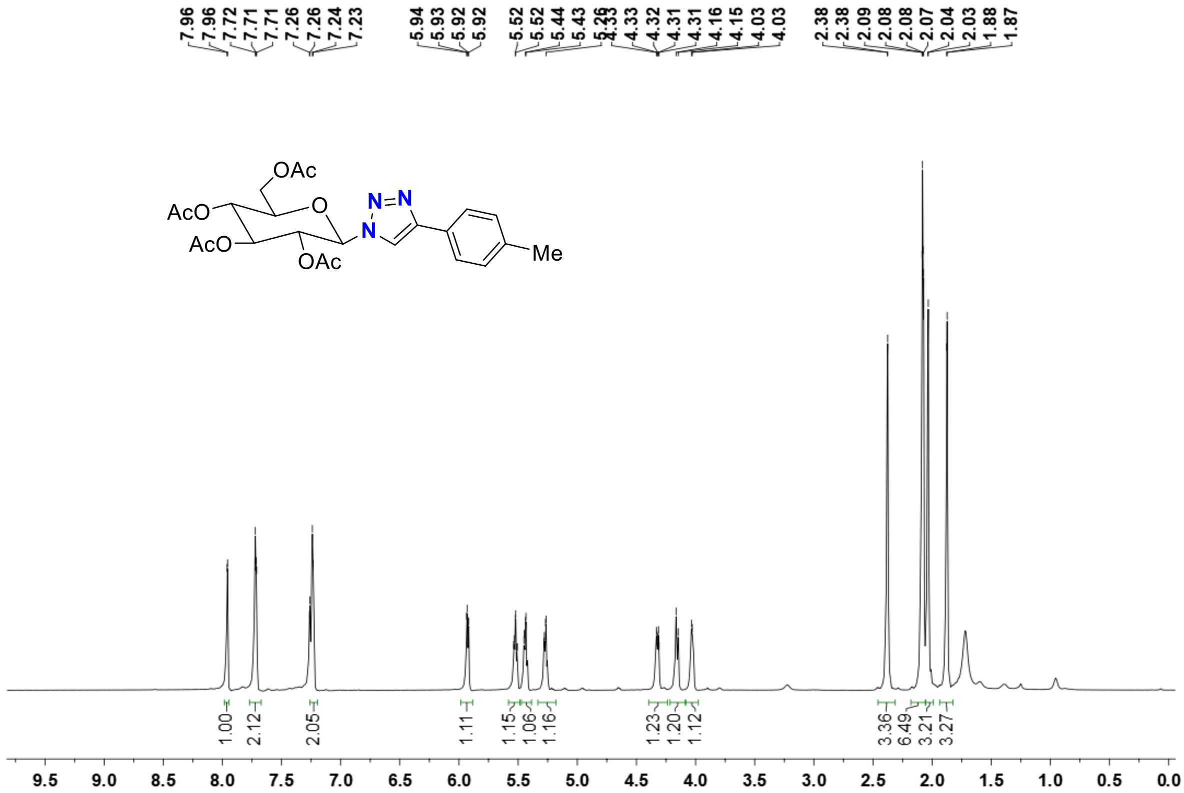

Figure S11. ^{1}H NMR (400 MHz) spectrum of (2R,3R,4S,5R,6R)-2-(acetoxymethyl)-6-(4-(p-tolyl)-1H-1,2,3-triazol-1-yl)tetrahydro-2H-pyran-3,4,5-triyl triacetate **(B_1)** $CDCl_3$ at r.t.

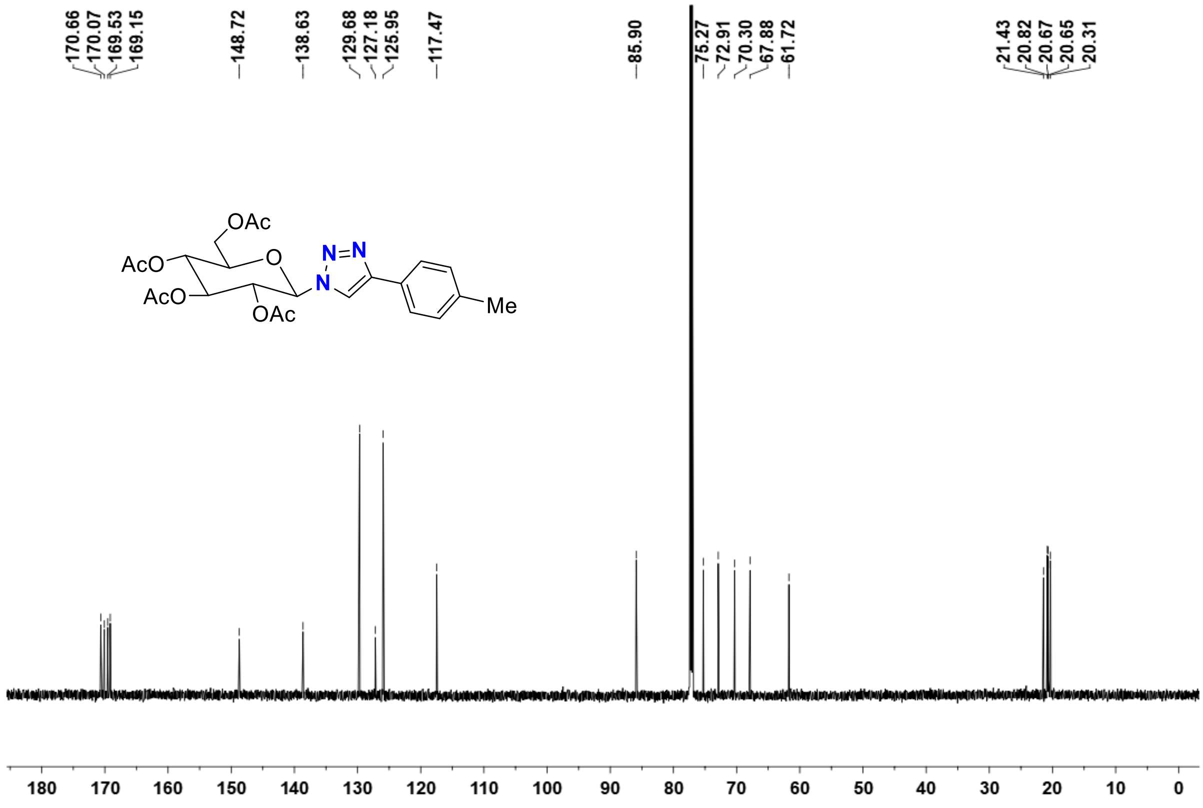

Figure S12. ^{13}C NMR (101 MHz) spectrum of (2R,3R,4S,5R,6R)-2-(acetoxymethyl)-6-(4-(p-tolyl)-1H-1,2,3-triazol-1-yl)tetrahydro-2H-pyran-3,4,5-triyl triacetate **(B_1)** in $CDCl_3$ at

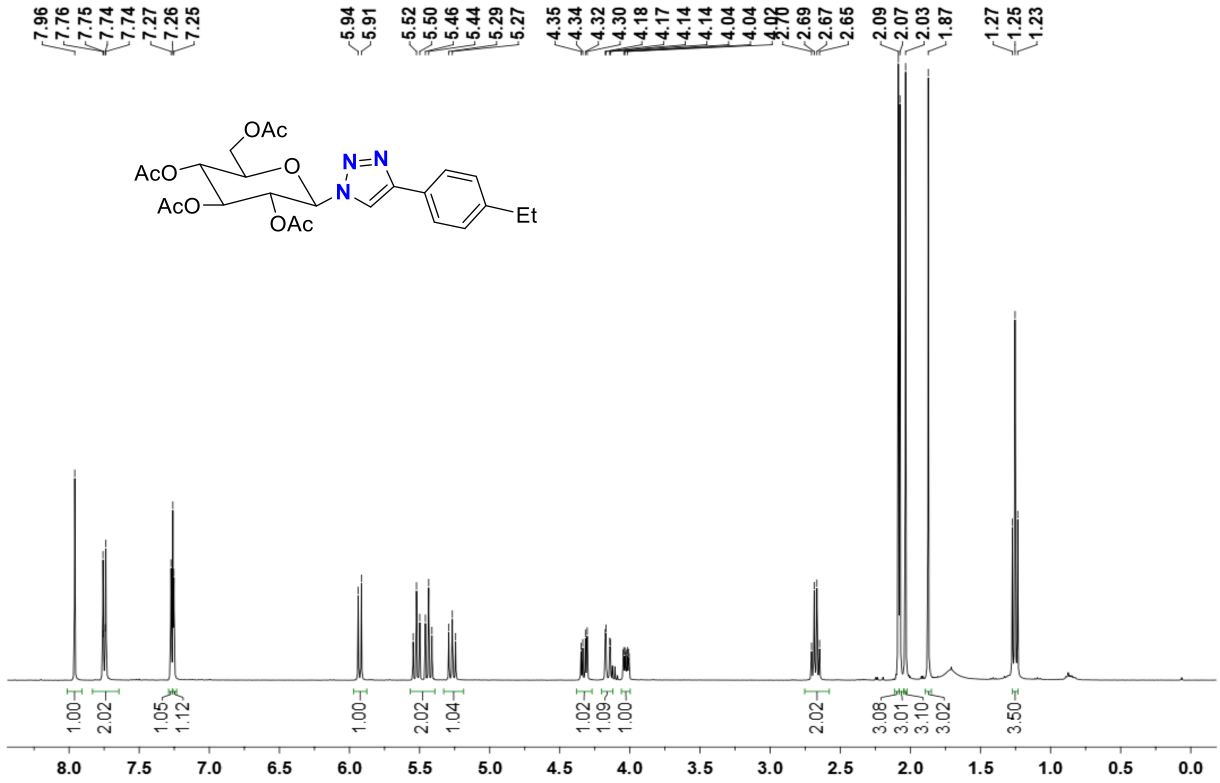

Figure S13. ^{1}H NMR (400 MHz) spectrum (2R,3R,4S,5R,6R)-2-(acetoxymethyl)-6-(4-(4-ethylphenyl)-1H-1,2,3-triazol-1-yl)tetrahydro-2H-pyran-3,4,5-triyl triacetate **(B_2)** $CDCl_3$ at r.t.

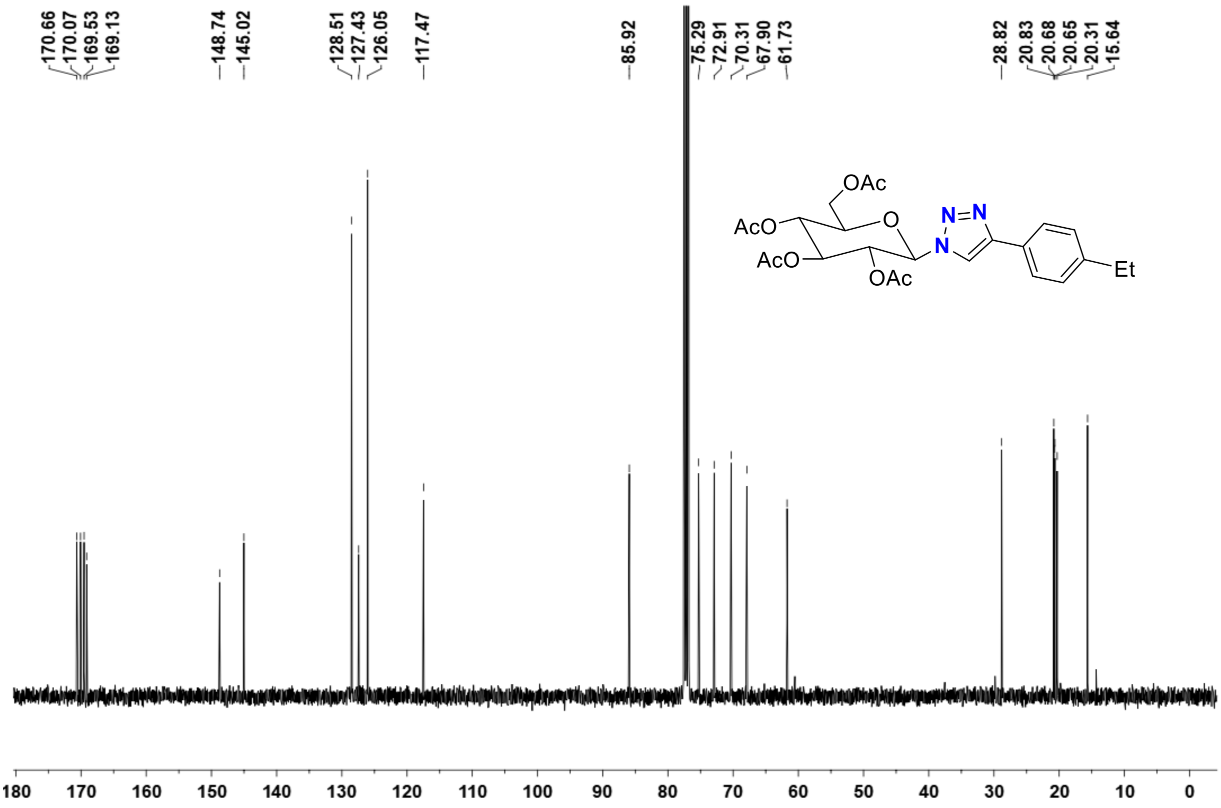

Figure S14. ^{13}C NMR (400 MHz) spectrum of (2R,3R,4S,5R,6R)-2-(acetoxymethyl)-6-(4-(4-ethylphenyl)-1H-1,2,3-triazol-1-yl)tetrahydro-2H-pyran-3,4,5-triyl triacetate **(B_2)** $CDCl_3$ at r.t.

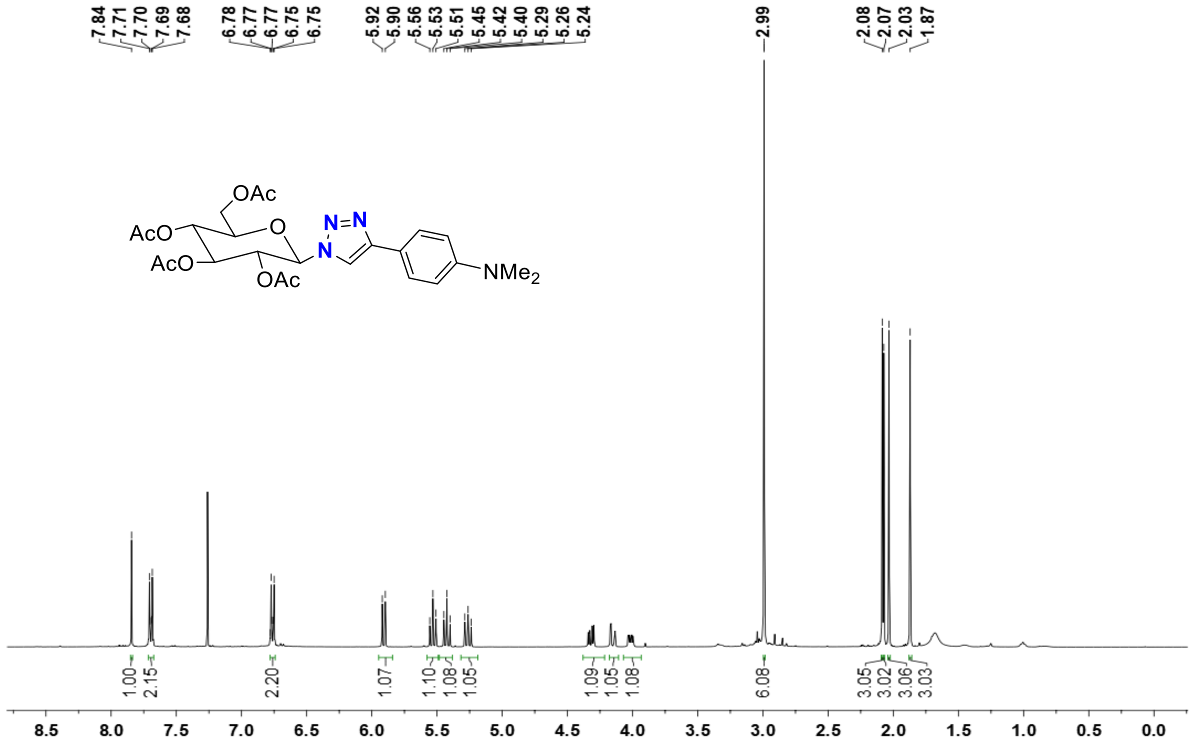

Figure S15. ^{1}H NMR (400 MHz) spectrum of(2R,3R,4S,5R,6R)-2-(acetoxymethyl)-6-(4-(4-(dimethylamino)phenyl)-1H-1,2,3-triazol-1-yl)tetrahydro-2H-pyran-3,4,5-triyl triacetate **(B_3)** $CDCl_3$ at r.t.

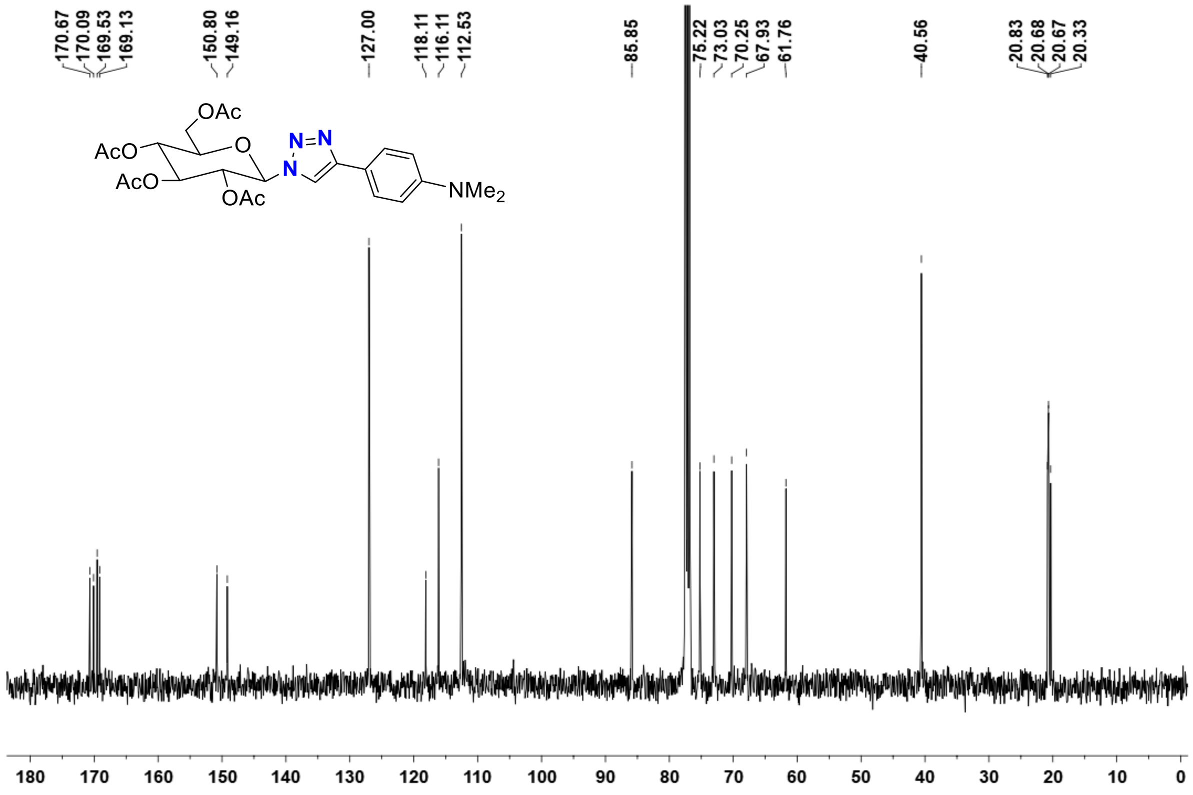

Figure S16. ^{13}C NMR (400 MHz) spectrum of (2R,3R,4S,5R,6R)-2-(acetoxymethyl)-6-(4-(4-(dimethylamino)phenyl)-1H-1,2,3-triazol-1-yl)tetrahydro-2H-pyran-3,4,5-triyl triacetate**(B_3)** $CDCl_3$ at r.t.

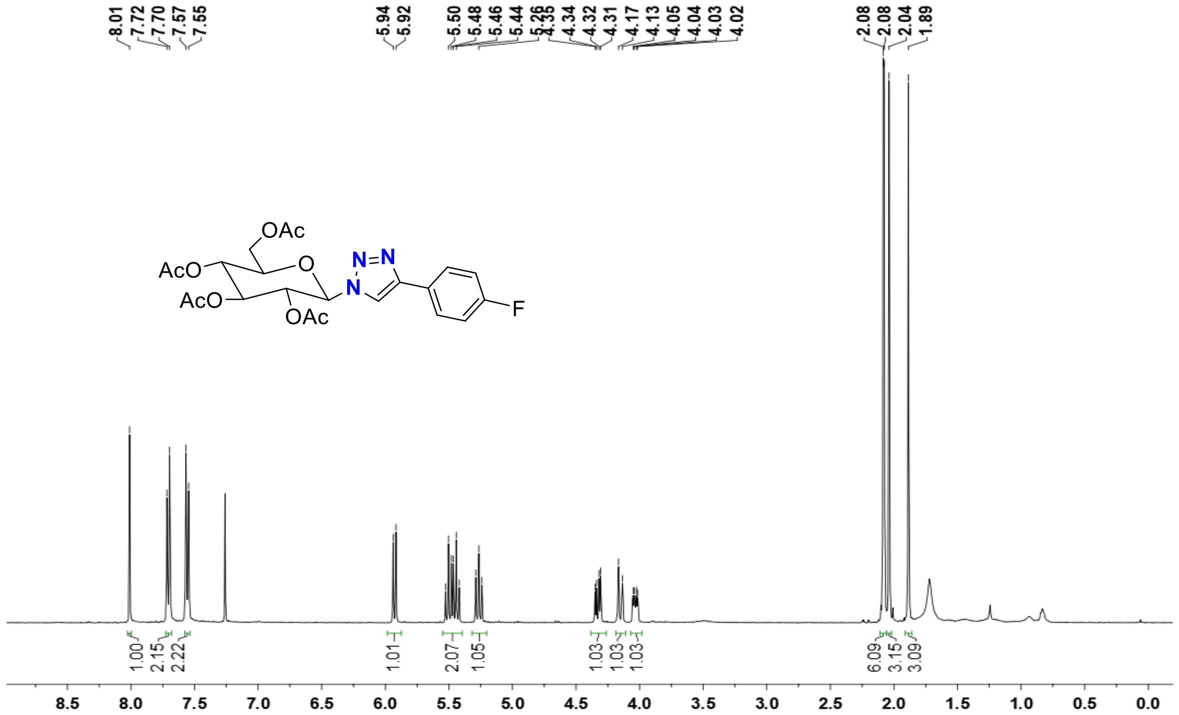

Figure S17. ^{1}H NMR (400 MHz) spectrum of (2R,3R,4S,5R,6R)-2-(acetoxymethyl)-6-(4-(4-fluorophenyl)-1H-1,2,3-triazol-1-yl)tetrahydro-2H-pyran-3,4,5-triyl triacetate **(B$_4$)** $CDCl_3$ at r.t.

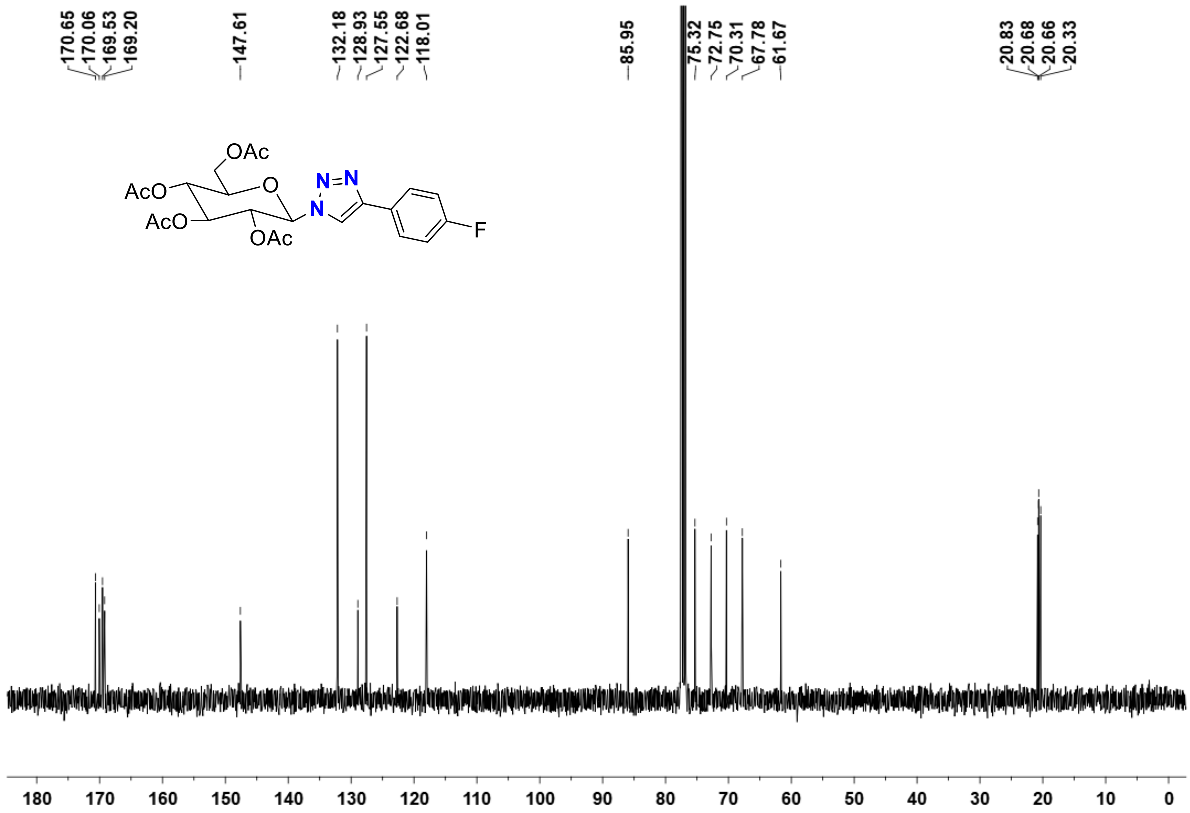

Figure S18. ^{13}C NMR (101 MHz) spectrum of (2R,3R,4S,5R,6R)-2-(acetoxymethyl)-6-(4-(4-fluorophenyl)-1H-1,2,3-triazol-1-yl)tetrahydro-2H-pyran-3,4,5-triyl triacetate **(B$_4$)** in $CDCl_3$ at r.t.

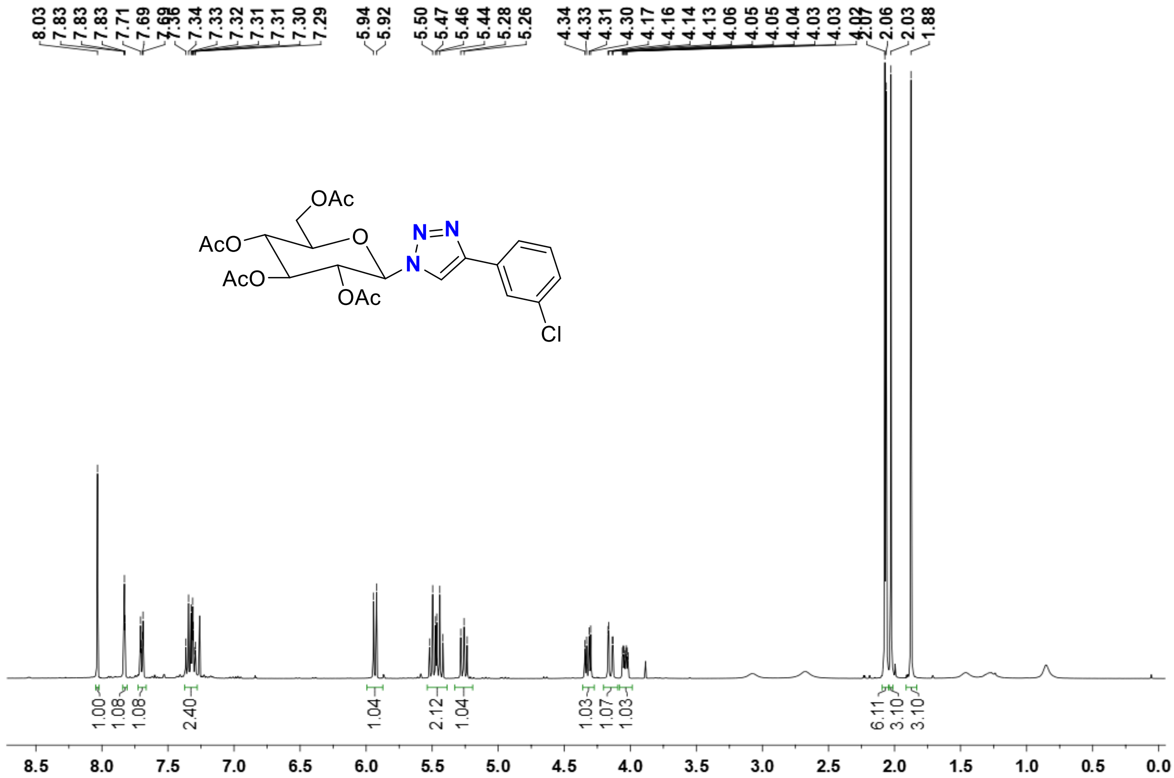

Figure S19. ^{1}H NMR (400 MHz) spectrum of (2R,3R,4S,5R,6R)-2-(acetoxymethyl)-6-(4-(3-chlorophenyl)-1H-1,2,3-triazol-1-yl)tetrahydro-2H-pyran-3,4,5-triyl triacetate **(B$_5$)** $CDCl_3$ at r.t.

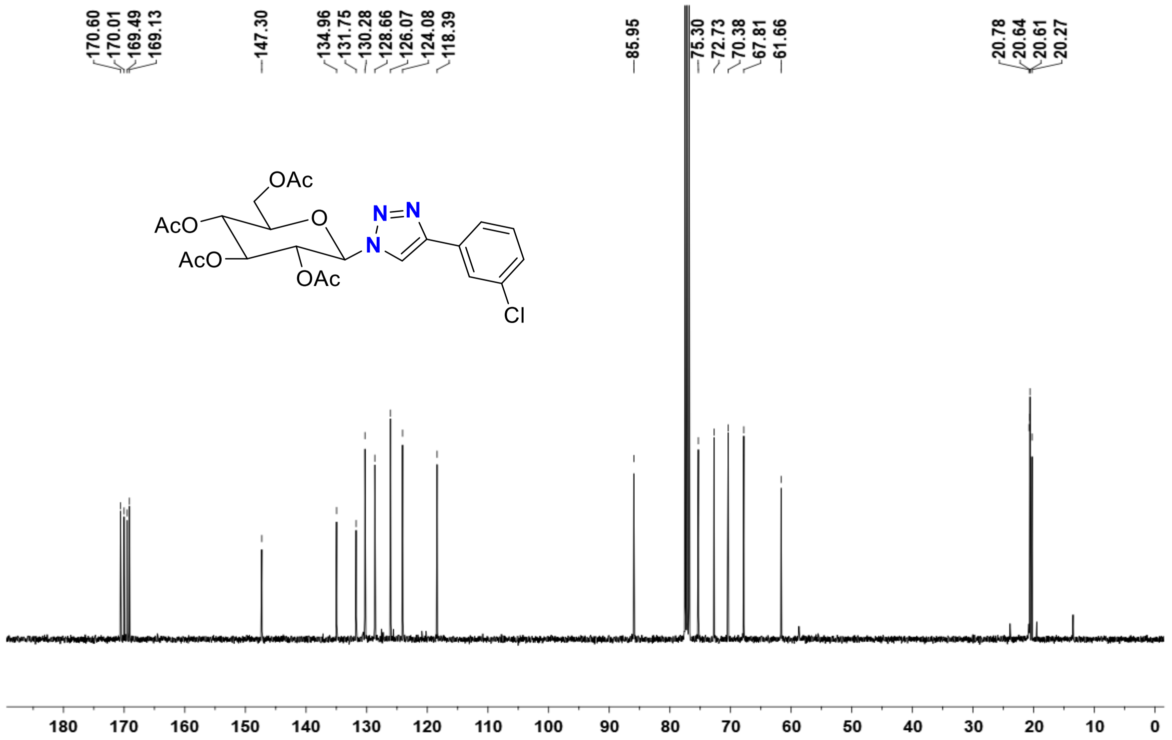

Figure S20. ^{13}C NMR (400 MHz) spectrum of(2R,3R,4S,5R,6R)-2-(acetoxymethyl)-6-(4-(3-chlorophenyl)-1H-1,2,3-triazol-1-yl)tetrahydro-2H-pyran-3,4,5-triyl triacetate **(B$_5$)** $CDCl_3$ at r.t.

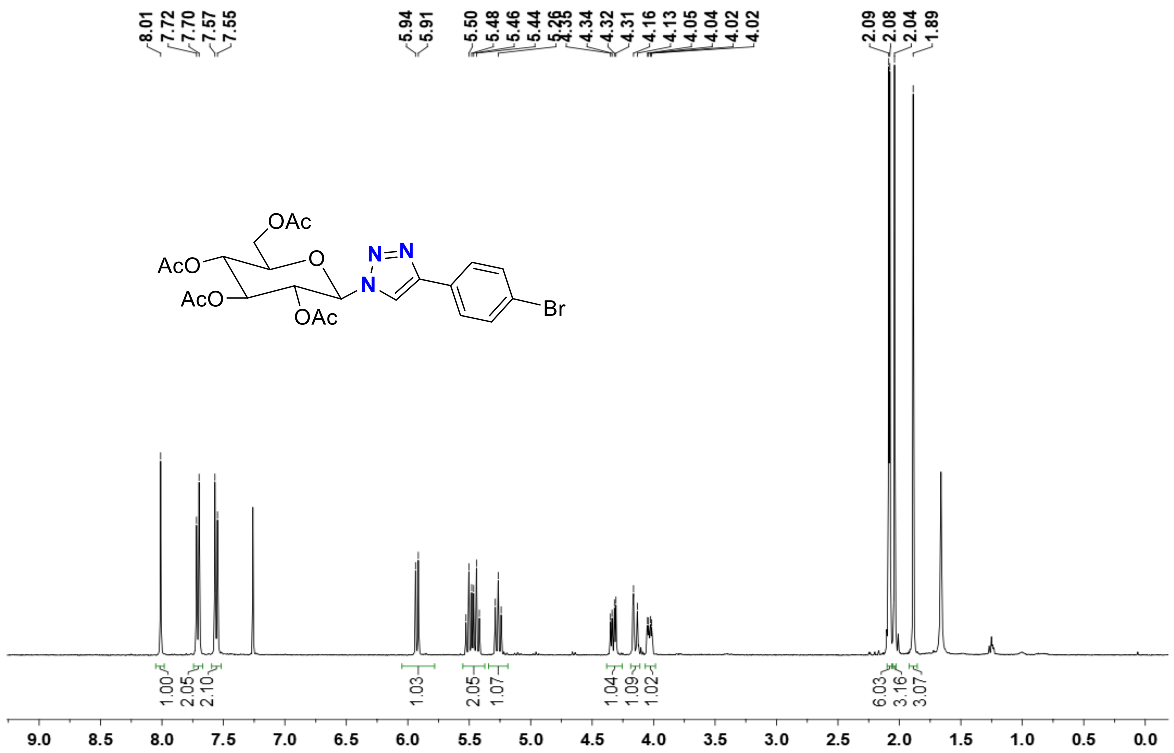

Figure S21. ^{1}H NMR (400 MHz) spectrum of (2R,3R,4S,5R,6R)-2-(acetoxymethyl)-6-(4-(4-bromophenyl)-1H-1,2,3-triazol-1-yl)tetrahydro-2H-pyran-3,4,5-triyl triacetate **(B_6)** $CDCl_3$ at r.t.

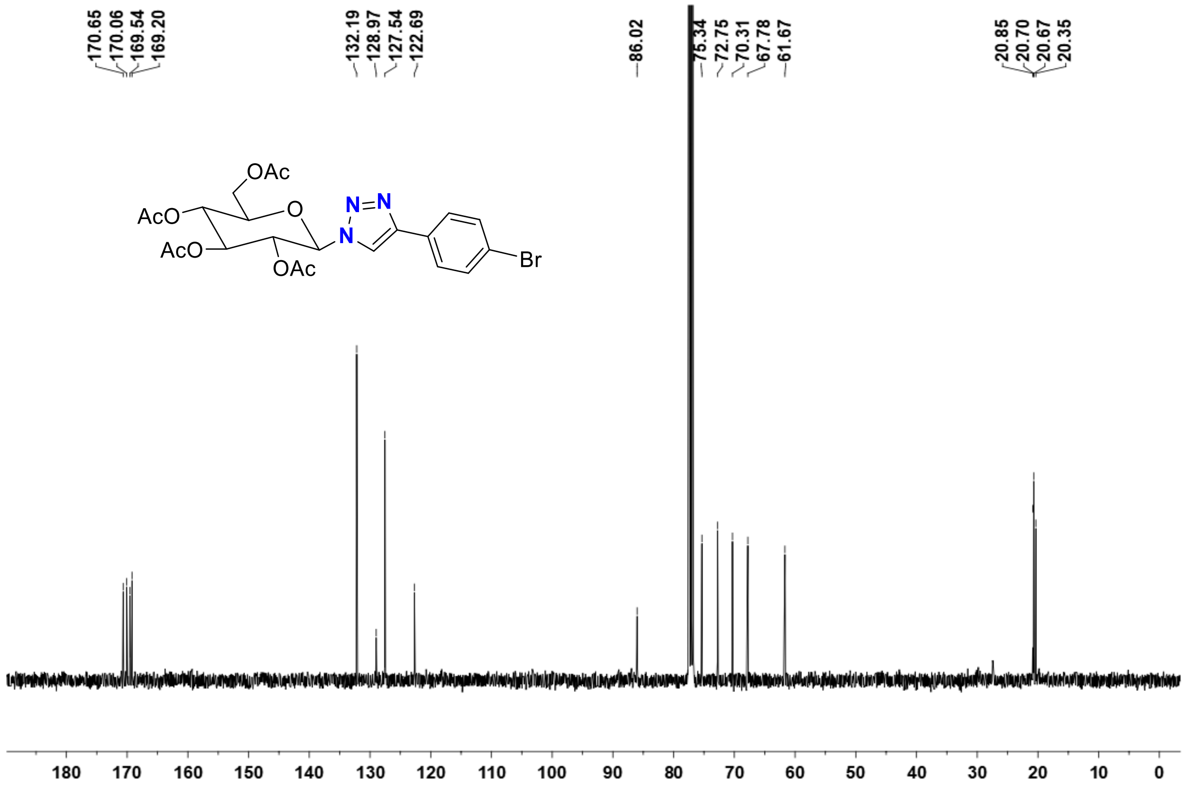

Figure S22. ^{13}C NMR (101 MHz) spectrum of (2R,3R,4S,5R,6R)-2-(acetoxymethyl)-6-(4-(4-bromophenyl)-1H-1,2,3-triazol-1-yl)tetrahydro-2H-pyran-3,4,5-triyl triacetate **(B_6)** in $CDCl_3$ at r.t.

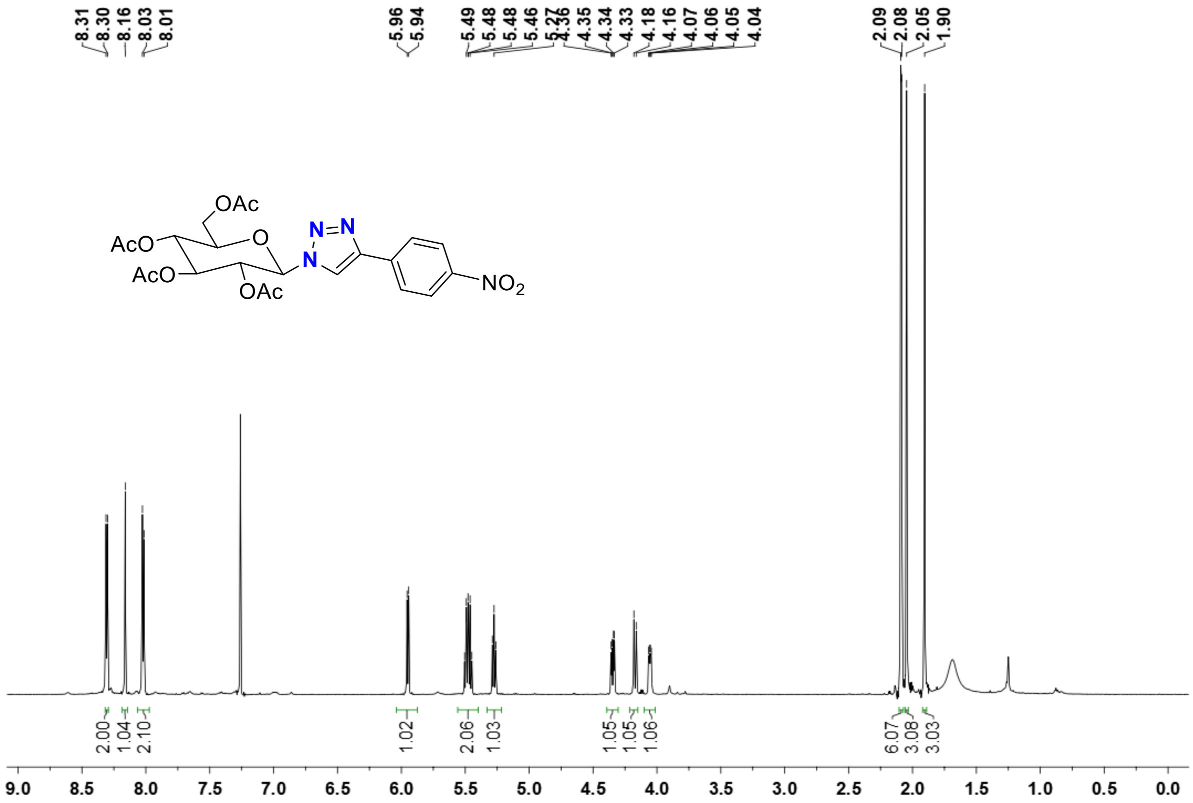

Figure S23. ^{1}H NMR (400 MHz) spectrum of (2R,3R,4S,5R,6R)-2-(acetoxymethyl)-6-(4-(4-nitrophenyl)-1H-1,2,3-triazol-1-yl)tetrahydro-2H-pyran-3,4,5-triyl triacetate **(B$_7$)** CDCl$_3$ at r.t.

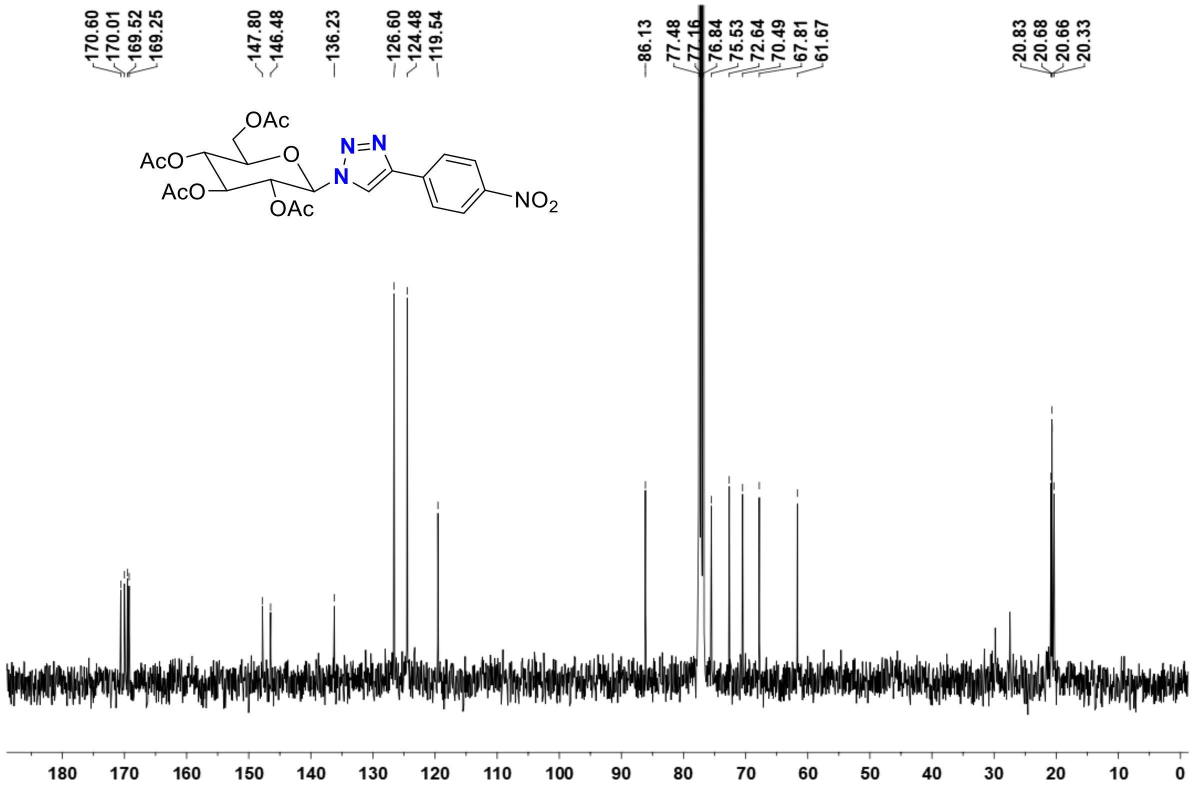

Figure S24. ^{13}C NMR (101 MHz) spectrum of (2R,3R,4S,5R,6R)-2-(acetoxymethyl)-6-(4-(4-nitrophenyl)-1H-1,2,3-triazol-1-yl)tetrahydro-2H-pyran-3,4,5-triyl triacetate **(B$_7$)** in CDCl$_3$ at r.t.

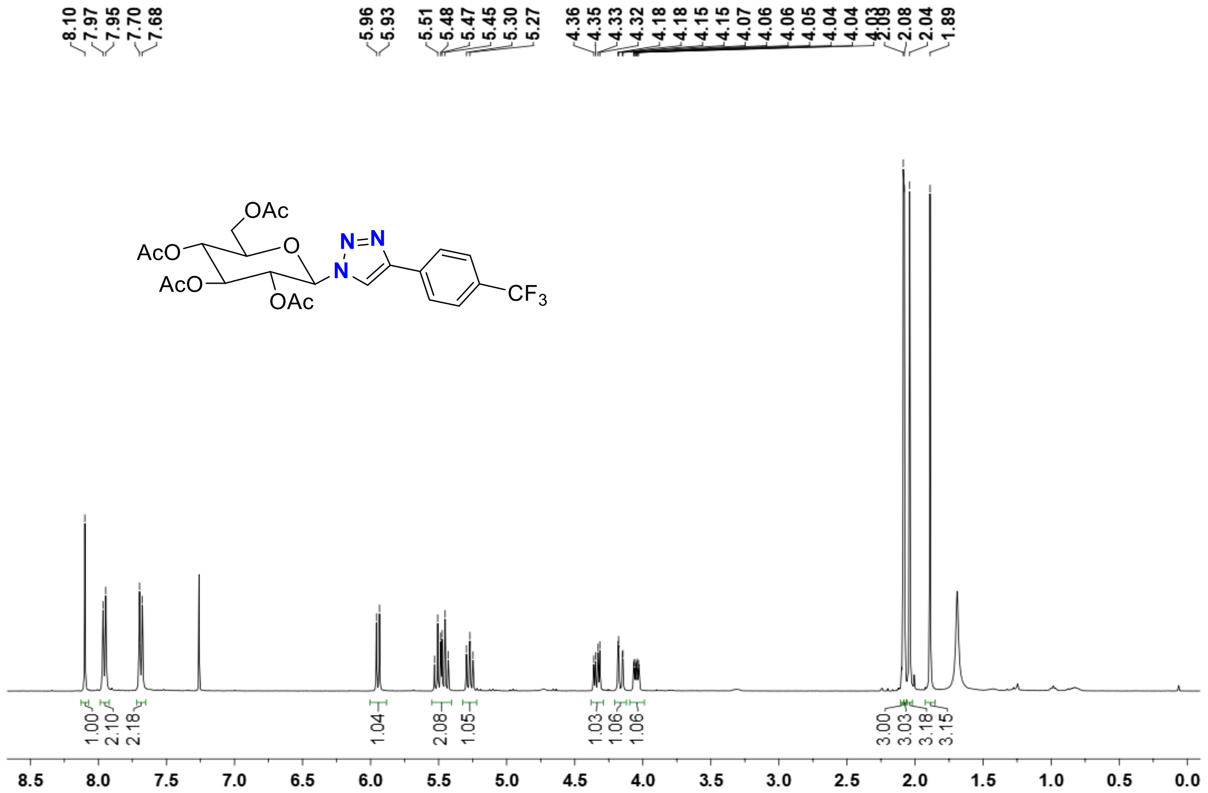

Figure S25. ^{1}H NMR (400 MHz) spectrum of (2R,3R,4S,5R,6R)-2-(acetoxymethyl)-6-(4-(4-(trifluoromethyl)phenyl)-1H-1,2,3-triazol-1-yl)tetrahydro-2H-pyran-3,4,5-triyl triacetate **(B_8)** $CDCl_3$ at r.t.

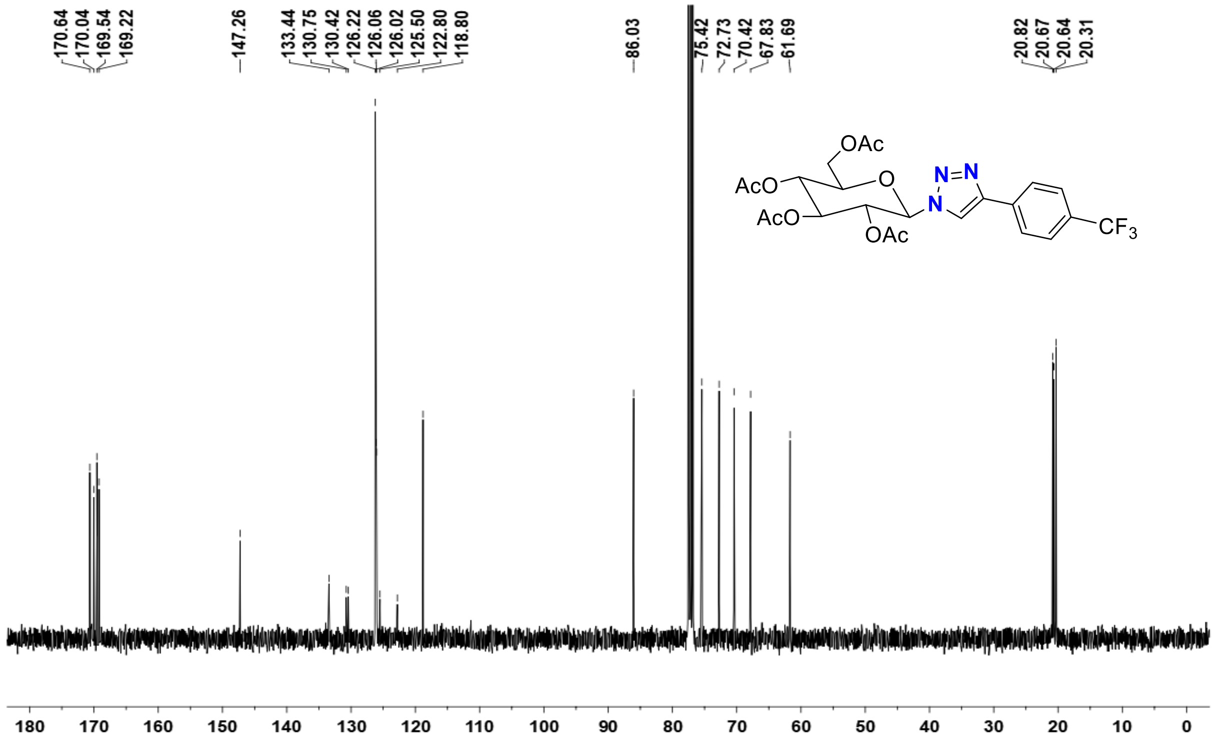

Figure S26. ^{13}C NMR (400 MHz) spectrum of (2R,3R,4S,5R,6R)-2-(acetoxymethyl)-6-(4-(4-(trifluoromethyl)phenyl)-1H-1,2,3-triazol-1-yl)tetrahydro-2H-pyran-3,4,5-triyl triacetate **(B_8)** $CDCl_3$ at r.t.

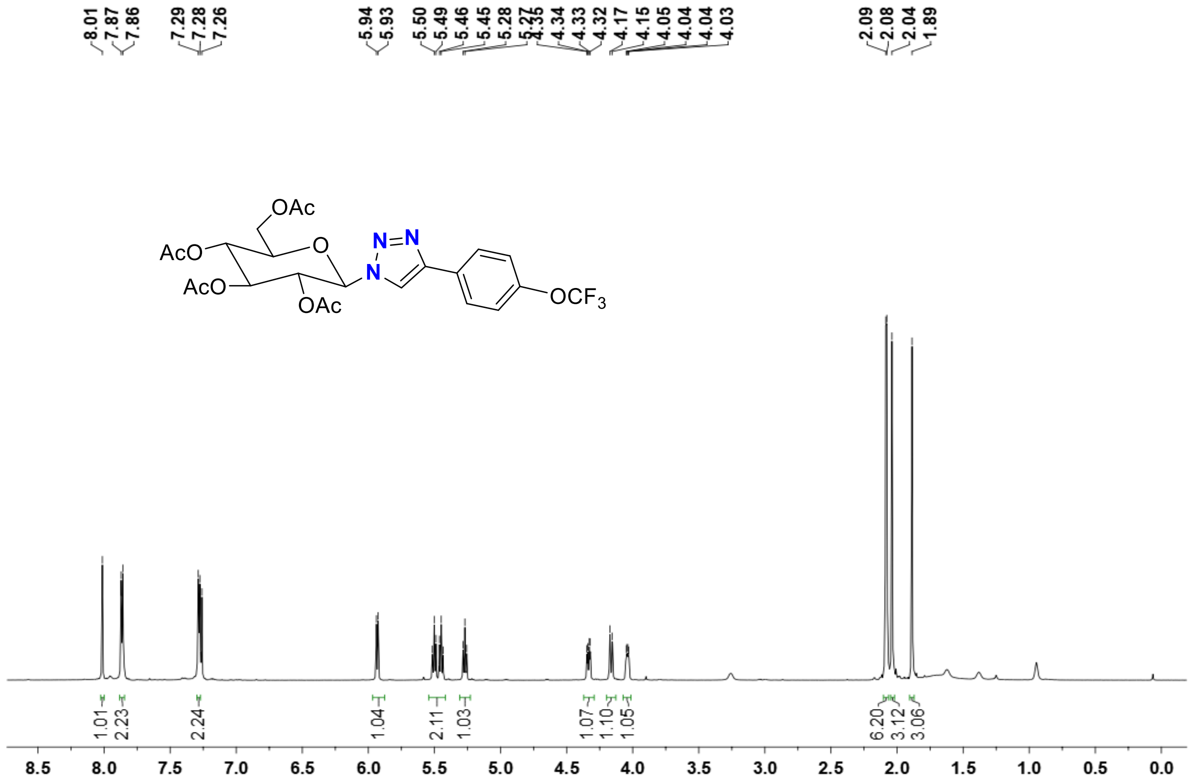

Figure S27. ^{1}H NMR (400 MHz) spectrum of(2R,3R,4S,5R,6R)-2-(acetoxymethyl)-6-(4-(4-(trifluoromethoxy)phenyl)-1H-1,2,3-triazol-1-yl)tetrahydro-2H-pyran-3,4,5-triyl triacetate **(B_9)** $CDCl_3$ at r.t.

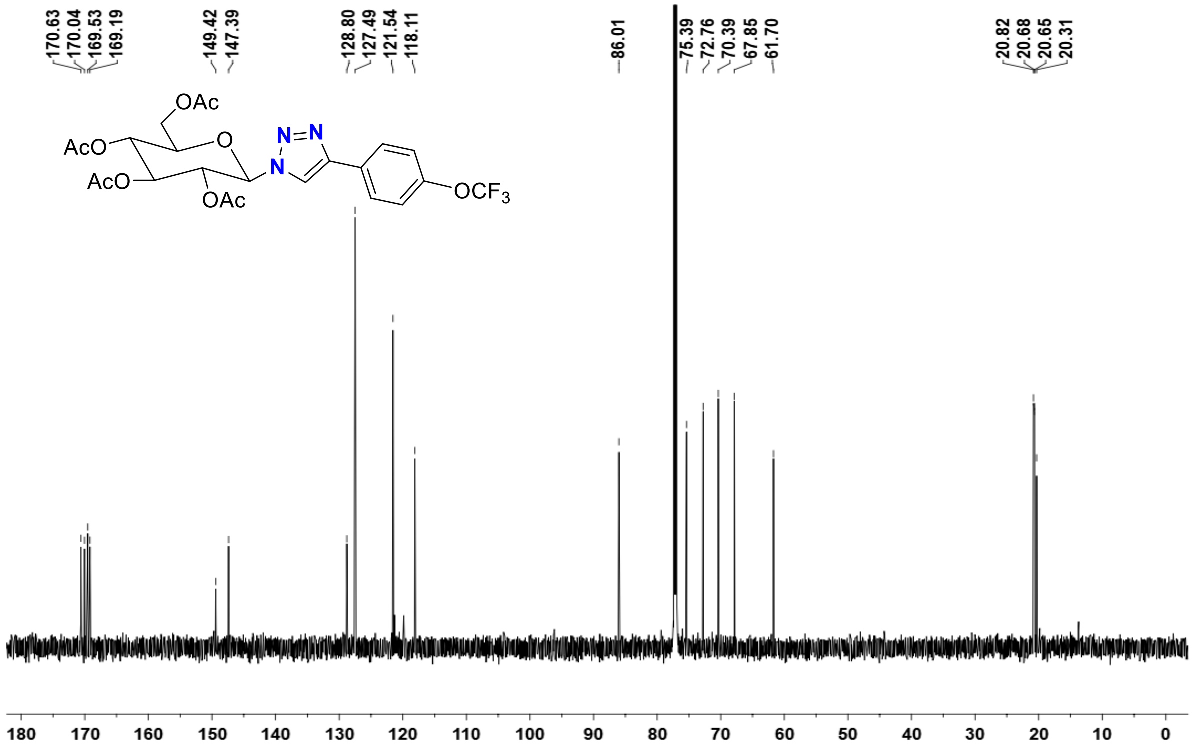

Figure S28. ^{13}C NMR (101 MHz) spectrum of (2R,3R,4S,5R,6R)-2-(acetoxymethyl)-6-(4-(4-(trifluoromethoxy)phenyl)-1H-1,2,3-triazol-1-yl)tetrahydro-2H-pyran-3,4,5-triyl triacetate **(B_9)** in $CDCl_3$ at r.t.

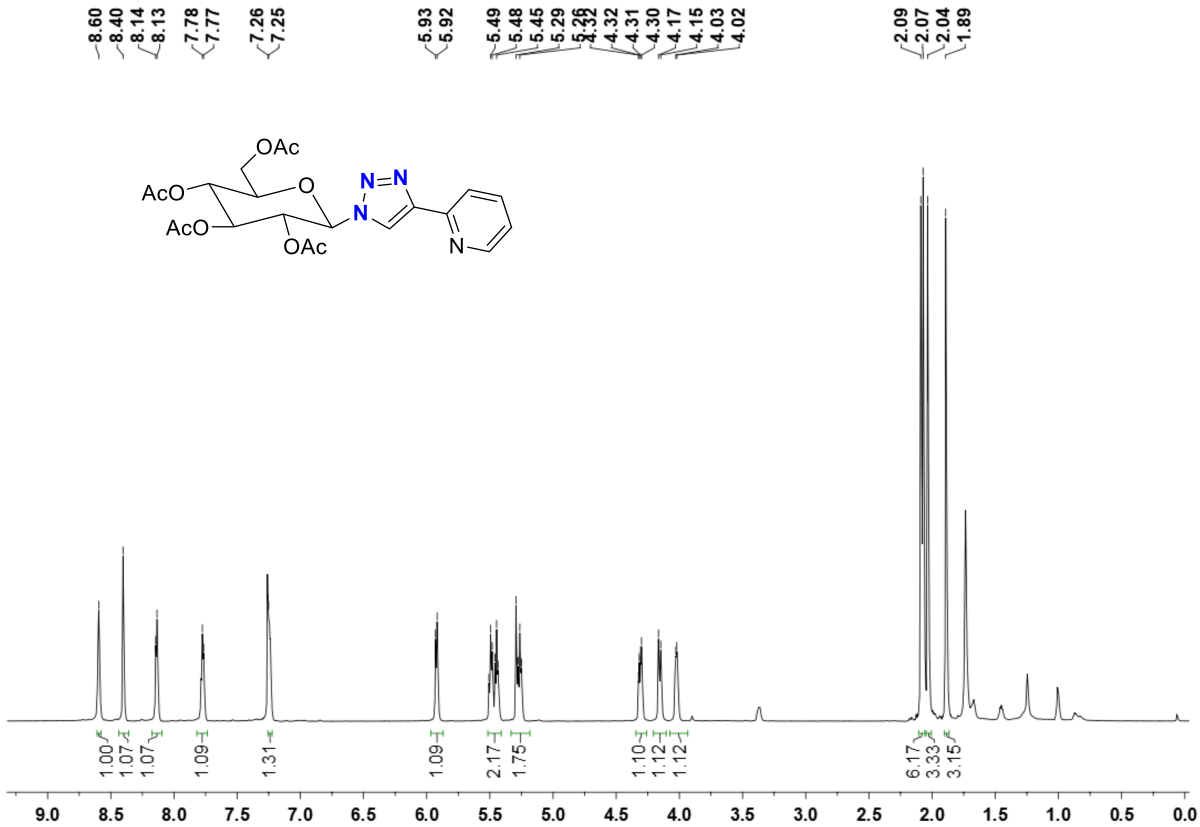

Figure S29. ^{1}H NMR (400 MHz) spectrum of (2R,3R,4S,5R,6R)-2-(acetoxymethyl)-6-(4-(pyridin-2-yl)-1H-1,2,3-triazol-1-yl)tetrahydro-2H-pyran-3,4,5-triyl triacetate **(B_{10})** $CDCl_3$ at r.t.

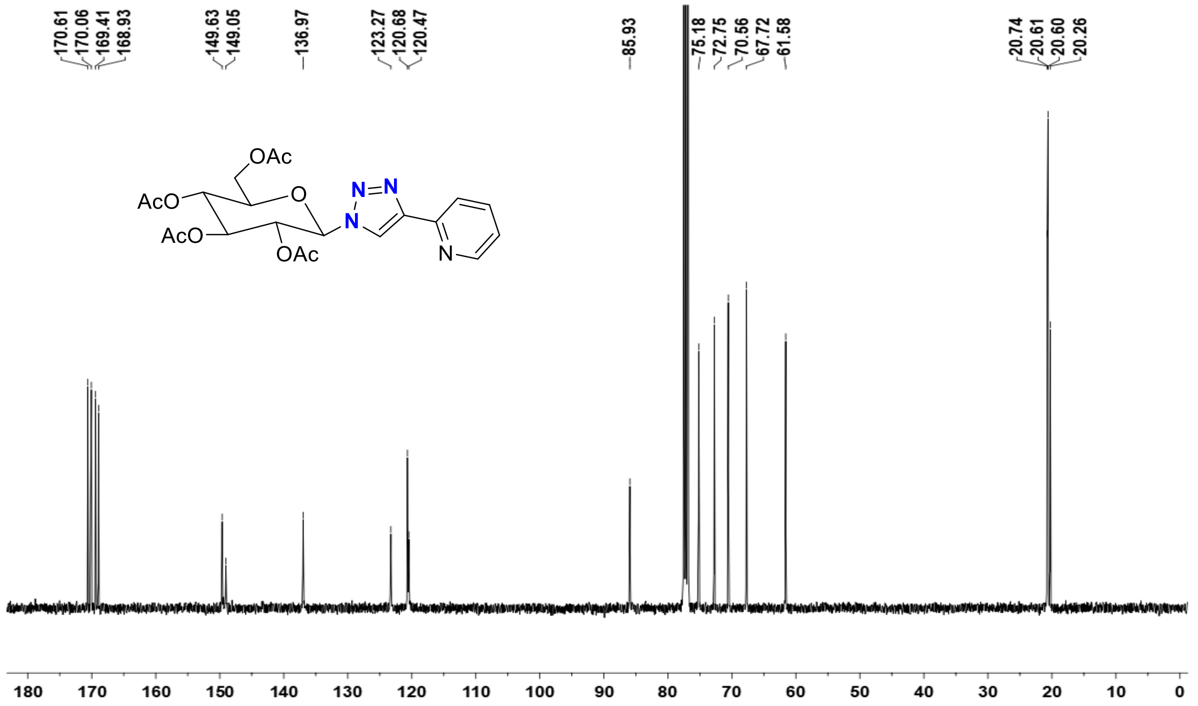

Figure S30. ^{13}C NMR (101 MHz) spectrum of (2R,3R,4S,5R,6R)-2-(acetoxymethyl)-6-(4-(pyridin-2-yl)-1H-1,2,3-triazol-1-yl)tetrahydro-2H-pyran-3,4,5-triyl triacetate **(B_{10})** in $CDCl_3$ at r.t.

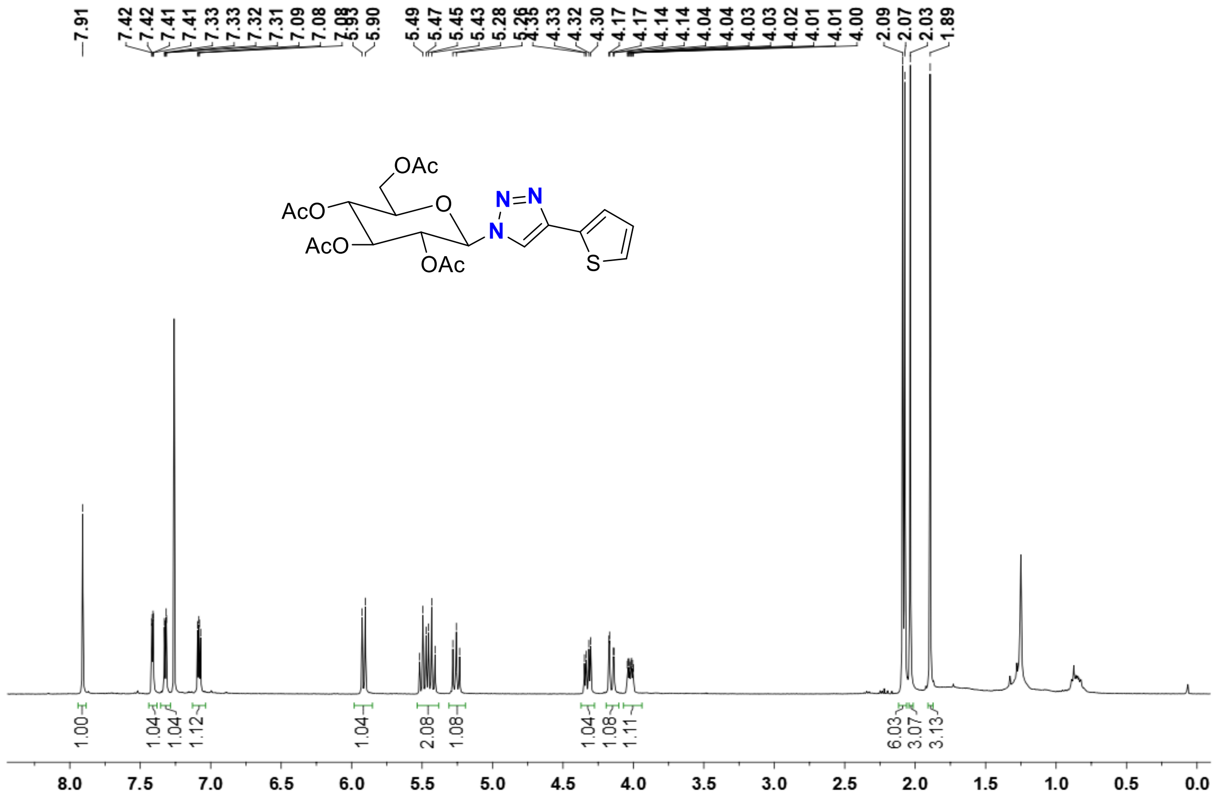

Figure S31. ^{1}H NMR (400 MHz) spectrum of (2R,3R,4S,5R,6R)-2-(acetoxymethyl)-6-(4-(thiophen-2-yl)-1H-1,2,3-triazol-1-yl)tetrahydro-2H-pyran-3,4,5-triyl triacetate **(B_{11})** $CDCl_3$ at r.t.

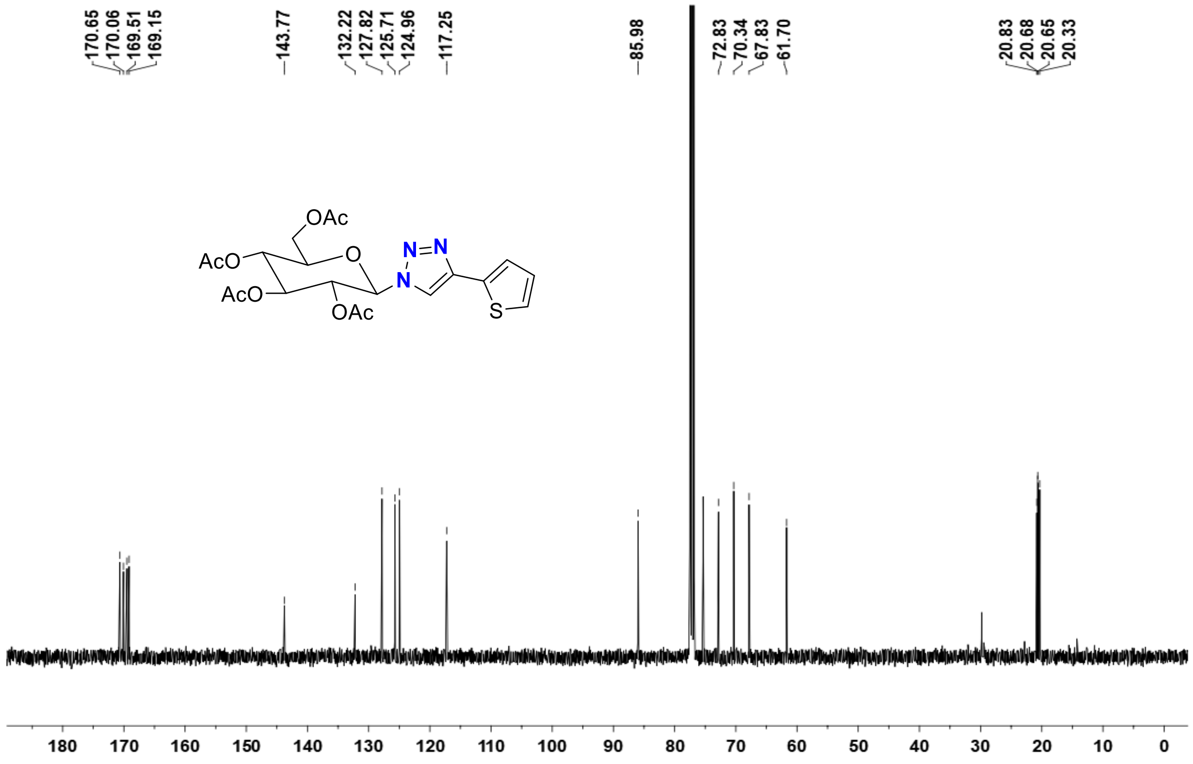

Figure S32. ^{13}C NMR (400 MHz) spectrum of (2R,3R,4S,5R,6R)-2-(acetoxymethyl)-6-(4-(thiophen-2-yl)-1H-1,2,3-triazol-1-yl)tetrahydro-2H-pyran-3,4,5-triyl triacetate **(B_{11})** $CDCl_3$ at r.t.

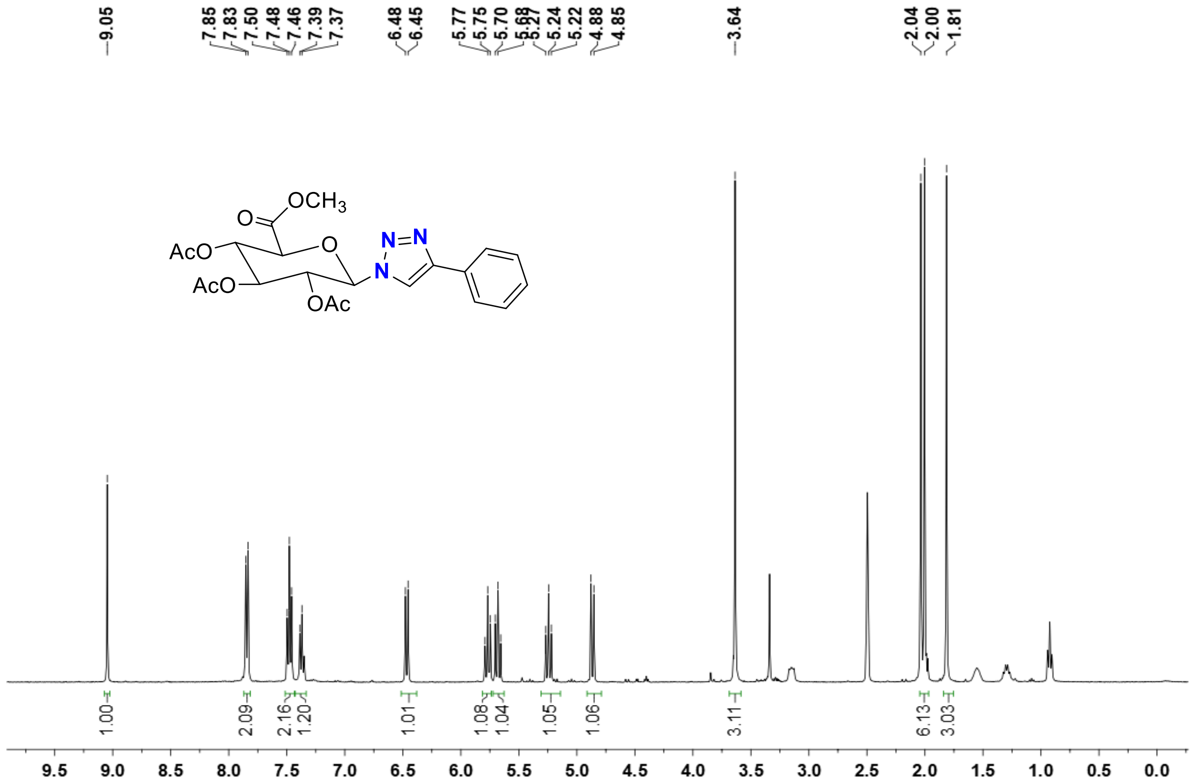

Figure S33. ^{1}H NMR (400 MHz) spectrum of (2S,3S,4S,5R,6R)-2-(methoxycarbonyl)-6-(4-phenyl-1H-1,2,3-triazol-1-yl)tetrahydro-2H-pyran-3,4,5-triyl triacetate **(C1)** DMSO at r.t.

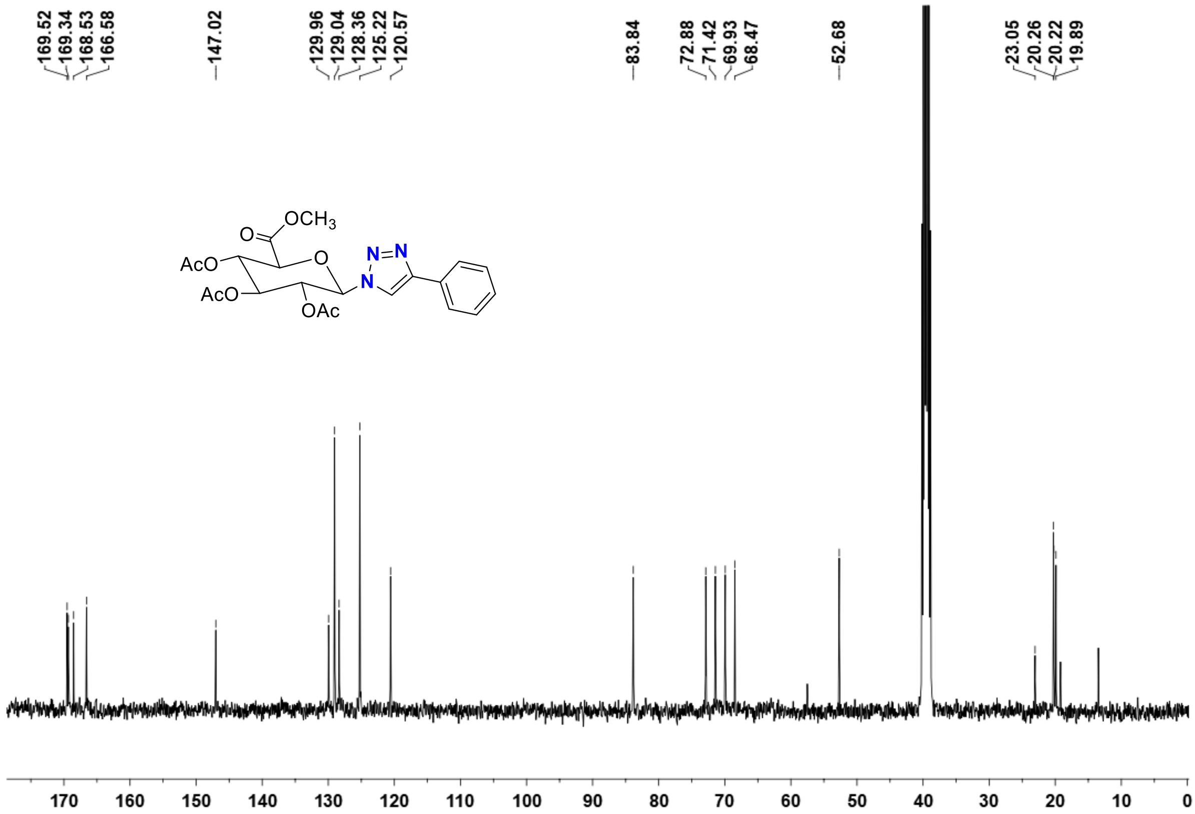

Figure S34. ^{13}C NMR (101 MHz) spectrum of (2S,3S,4S,5R,6R)-2-(methoxycarbonyl)-6-(4-phenyl-1H-1,2,3-triazol-1-yl)tetrahydro-2H-pyran-3,4,5-triyl triacetate **(C1)** in DMSO at r.t.

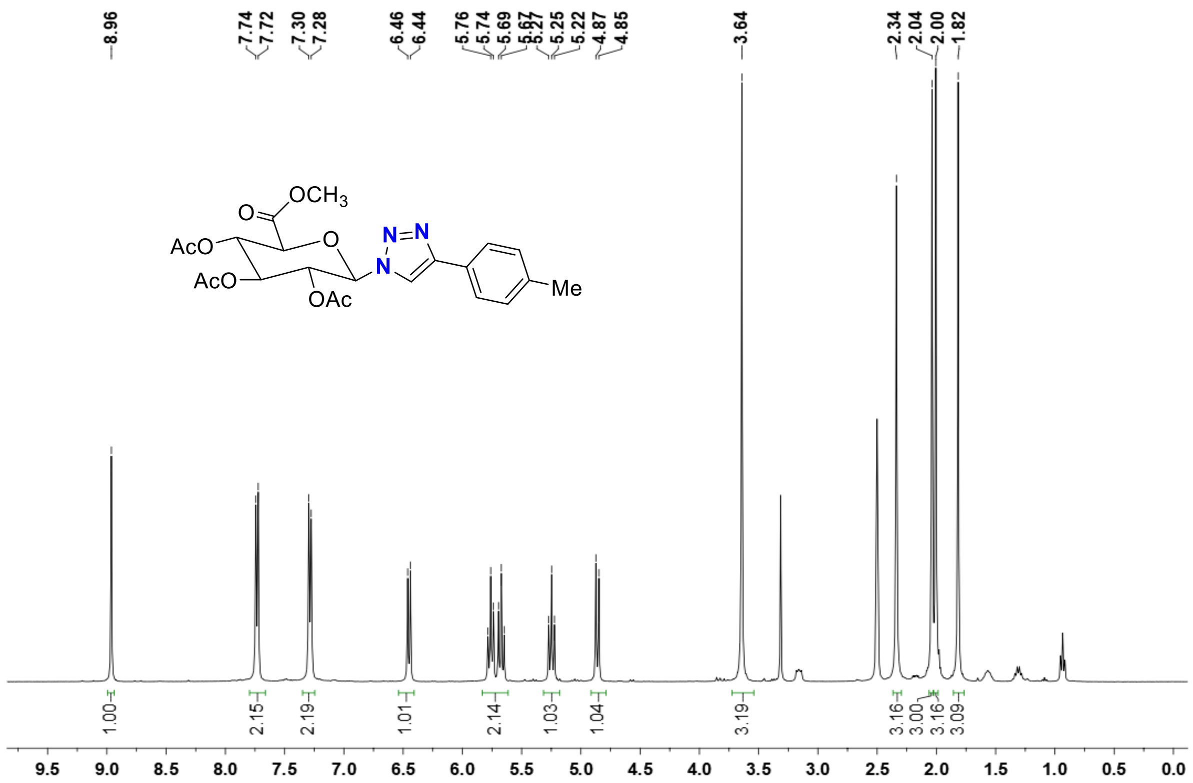

Figure S35. ^{1}H NMR (400 MHz) spectrum of (2S,3S,4S,5R,6R)-2-(methoxycarbonyl)-6-(4-(p-tolyl)-1H-1,2,3-triazol-1-yl)tetrahydro-2H-pyran-3,4,5-triyl triacetate **(C2)** DMSO at r.t.

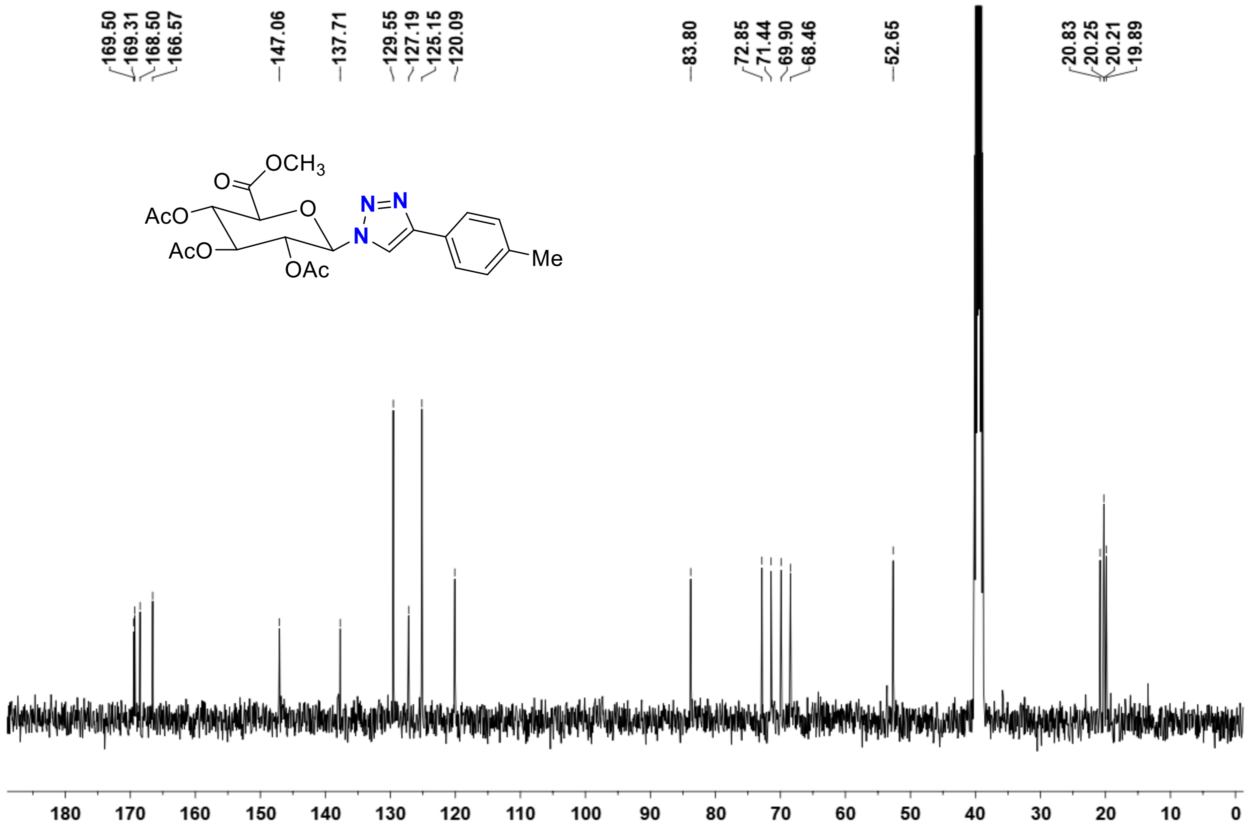

Figure S36. ^{13}C NMR (101 MHz) spectrum of (2S,3S,4S,5R,6R)-2-(methoxycarbonyl)-6-(4-(p-tolyl)-1H-1,2,3-triazol-1-yl)tetrahydro-2H-pyran-3,4,5-triyl triacetate **(C2)** in DMSO at r.t.

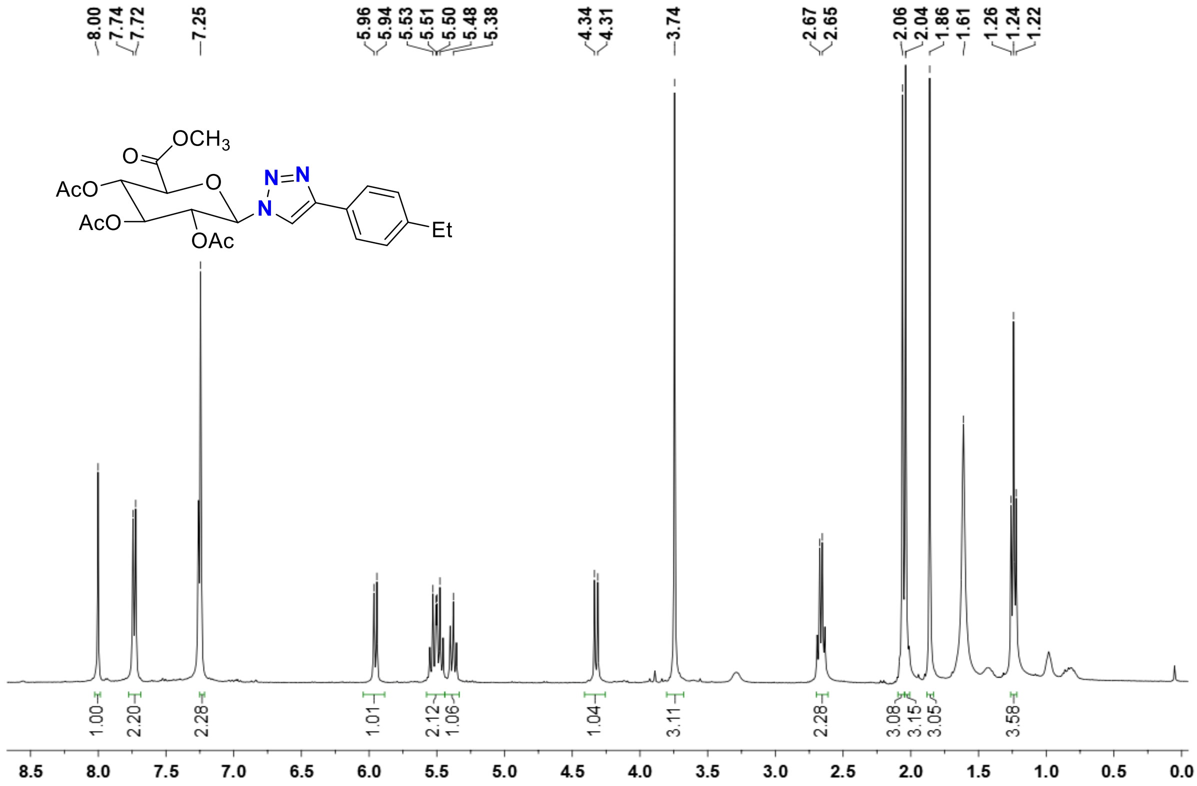

Figure S37. ^{1}H NMR (400 MHz) spectrum of(2R,3R,4S,5S,6S)-2-(4-(4-ethylphenyl)-1H-1,2,3-triazol-1-yl)-6-(methoxycarbonyl)tetrahydro-2H-pyran-3,4,5-triyl triacetate **(C3)** $CDCl_3$ at r.t.

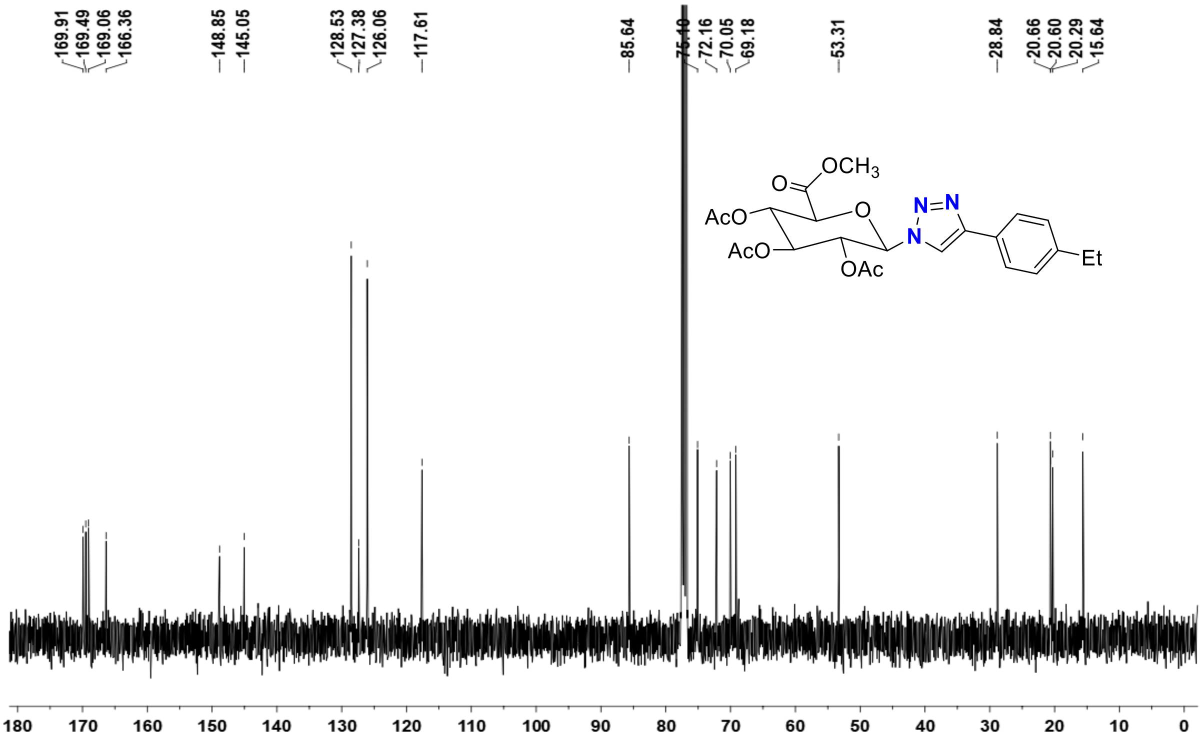

Figure S38. ^{13}C NMR (400 MHz) spectrum of (2R,3R,4S,5S,6S)-2-(4-(4-ethylphenyl)-1H-1,2,3-triazol-1-yl)-6-(methoxycarbonyl)tetrahydro-2H-pyran-3,4,5-triyl triacetate **(C3)** $CDCl_3$ at r.t.

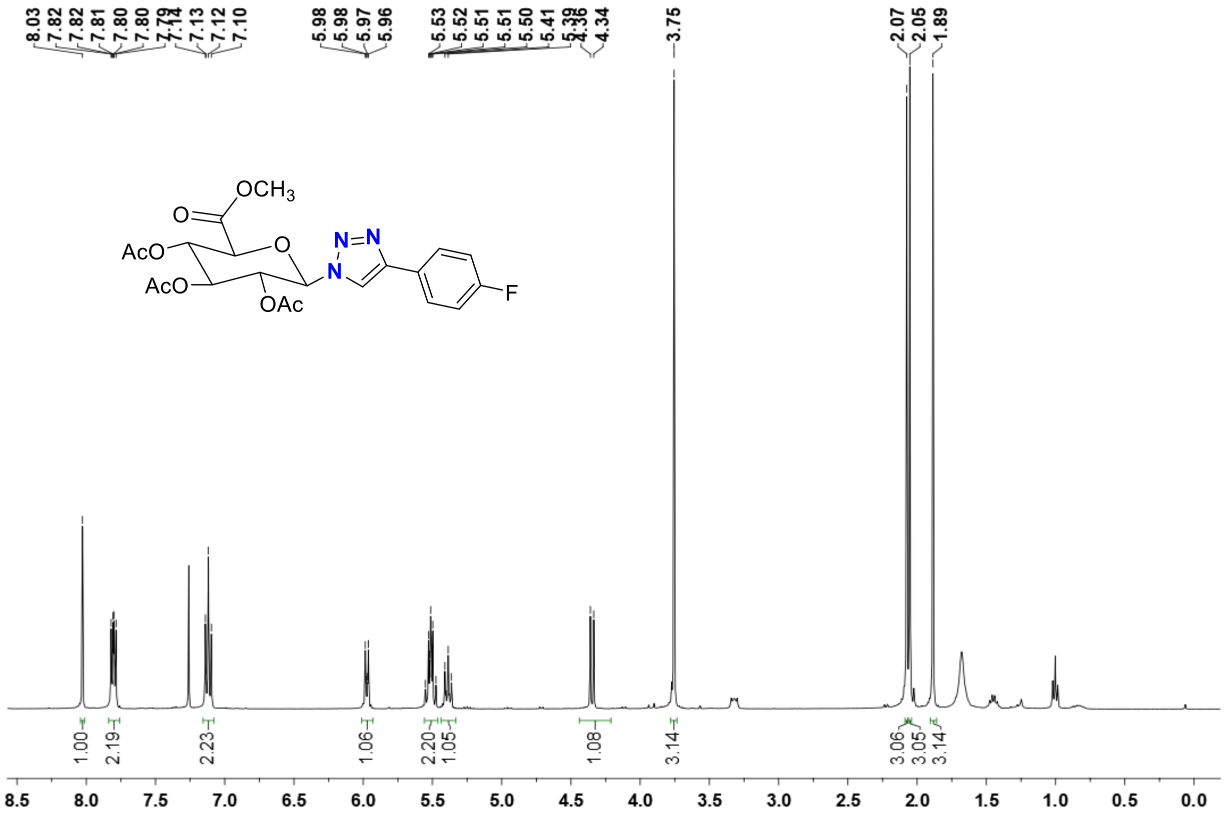

Figure S39. ^{1}H NMR (400 MHz) spectrum of (2R,3R,4S,5S,6S)-2-(4-(4-fluorophenyl)-1H-1,2,3-triazol-1-yl)-6-(methoxycarbonyl)tetrahydro-2H-pyran-3,4,5-triyl triacetate **(C4)** $CDCl_3$ at r.t.

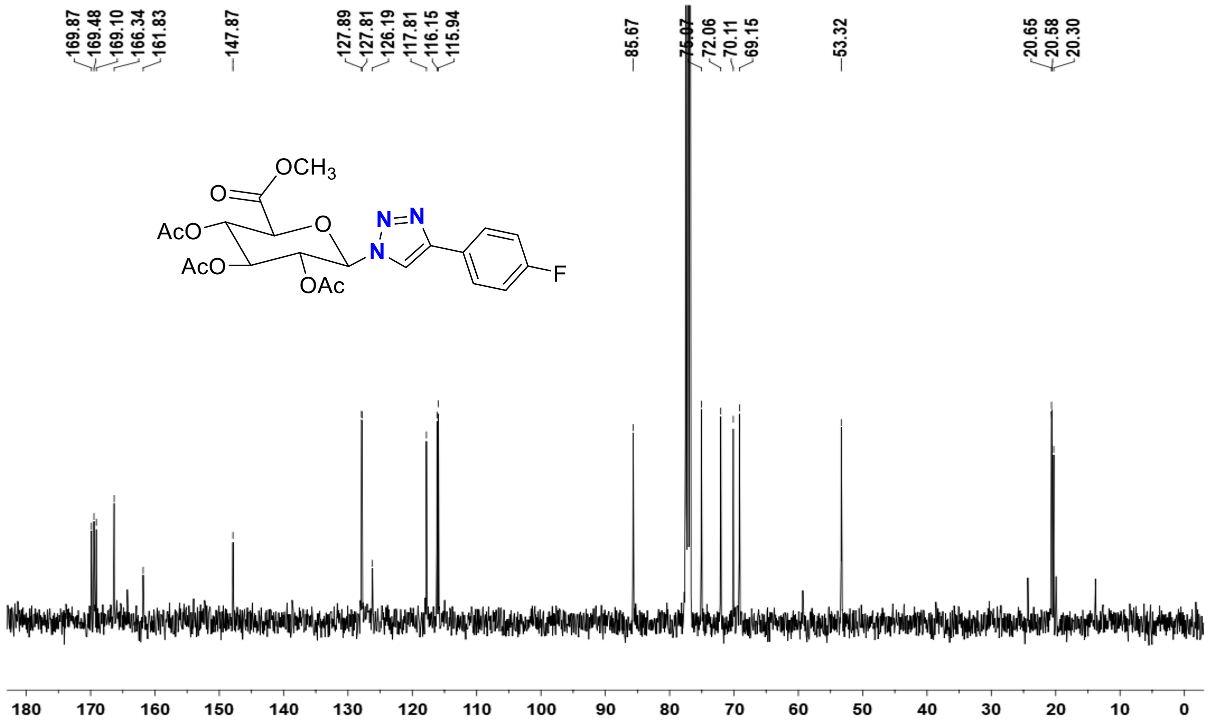

Figure S40. ^{13}C NMR (400 MHz) spectrum of (2R,3R,4S,5S,6S)-2-(4-(4-fluorophenyl)-1H-1,2,3-triazol-1-yl)-6-(methoxycarbonyl)tetrahydro-2H-pyran-3,4,5-triyl triacetate **(C4)** $CDCl_3$ at r.t.

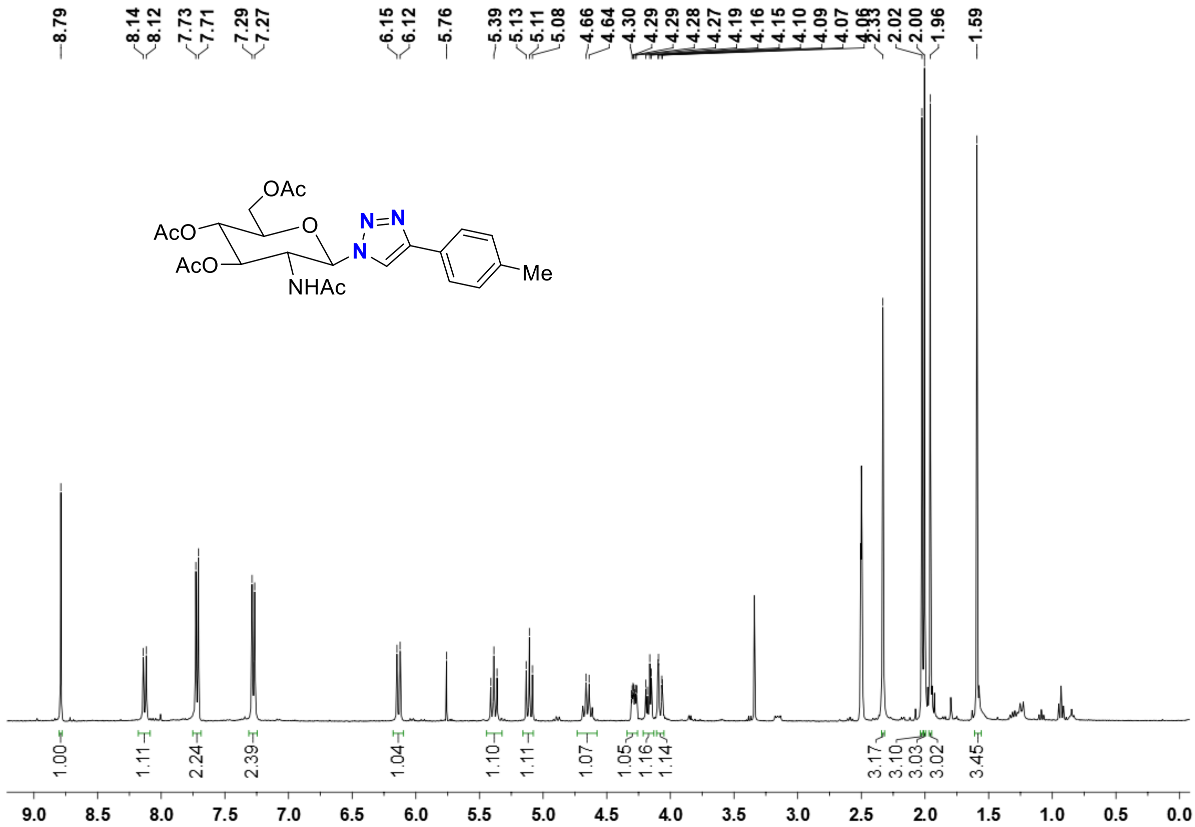

Figure S41. ^{1}H NMR (400 MHz) spectrum of (2R,3S,6S)-5-acetamido-2-(acetoxymethyl)-6-(4-(p-tolyl)-1H-1,2,3-triazol-1-yl)tetrahydro-2H-pyran-3,4-diyl diacetate **(D$_1$)** DMSO at r.t.

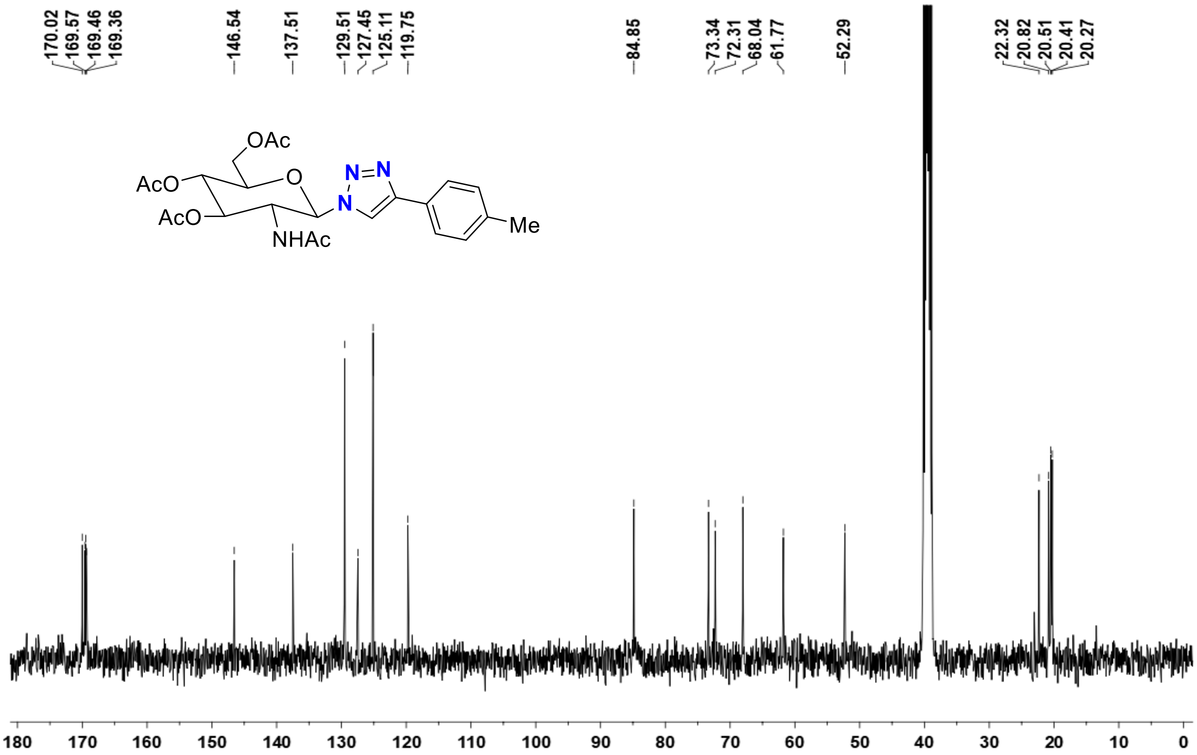

Figure S42. ^{13}C NMR (101 MHz) spectrum of (2R,3S,6S)-5-acetamido-2-(acetoxymethyl)-6-(4-(p-tolyl)-1H-1,2,3-triazol-1-yl)tetrahydro-2H-pyran-3,4-diyl diacetate **(D$_1$)** in DMSO at r.t.

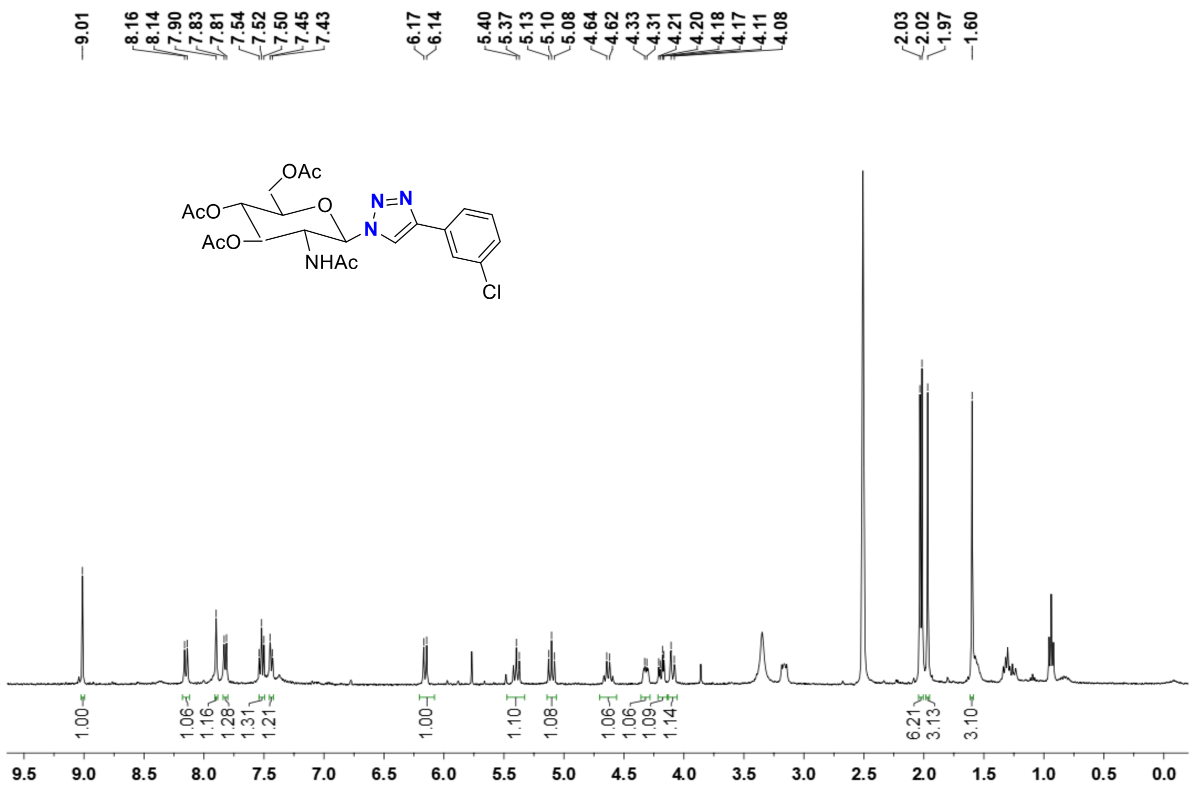

Figure S43. ^{1}H NMR (400 MHz) spectrum of (2R,3S,6S)-5-acetamido-2-(acetoxymethyl)-6-(4-(3-chlorophenyl)-1H-1,2,3-triazol-1-yl)tetrahydro-2H-pyran-3,4-diyl diacetate **(D_2)** DMSO at r.t.

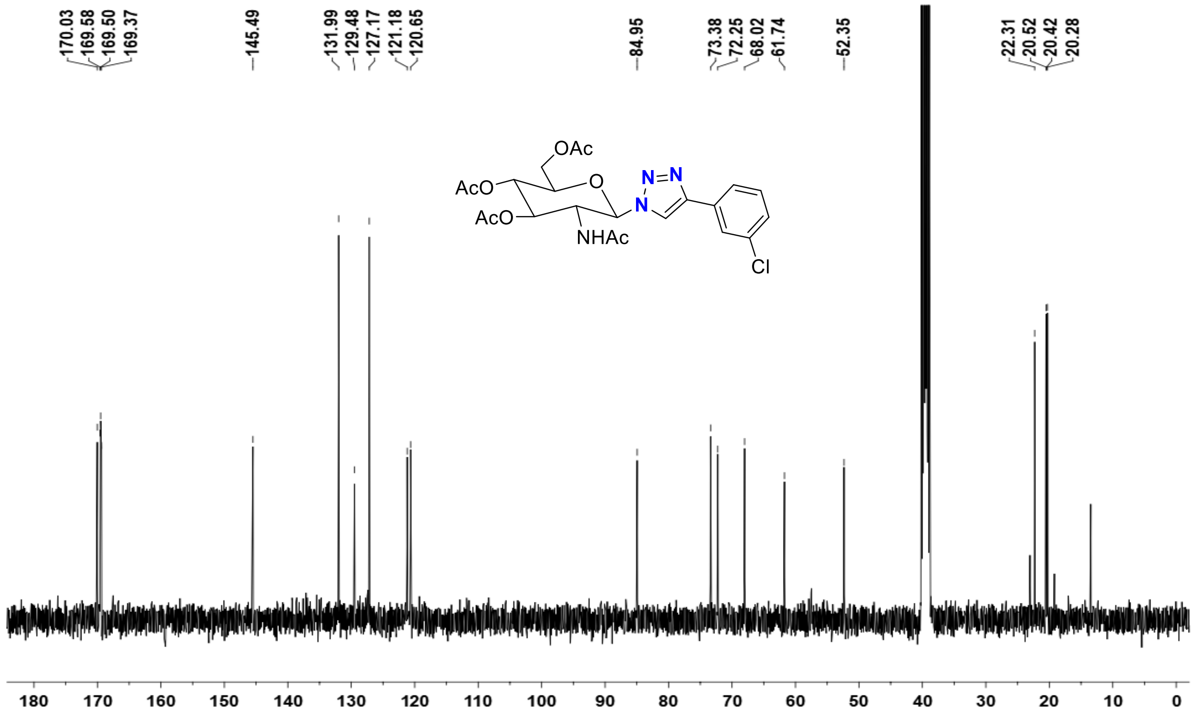

Figure S44. ^{13}C NMR (101 MHz) spectrum of(2R,3S,6S)-5-acetamido-2-(acetoxymethyl)-6-(4-(3-chlorophenyl)-1H-1,2,3-triazol-1-yl)tetrahydro-2H-pyran-3,4-diyl diacetate **(D_2)** in DMSO at r.t.

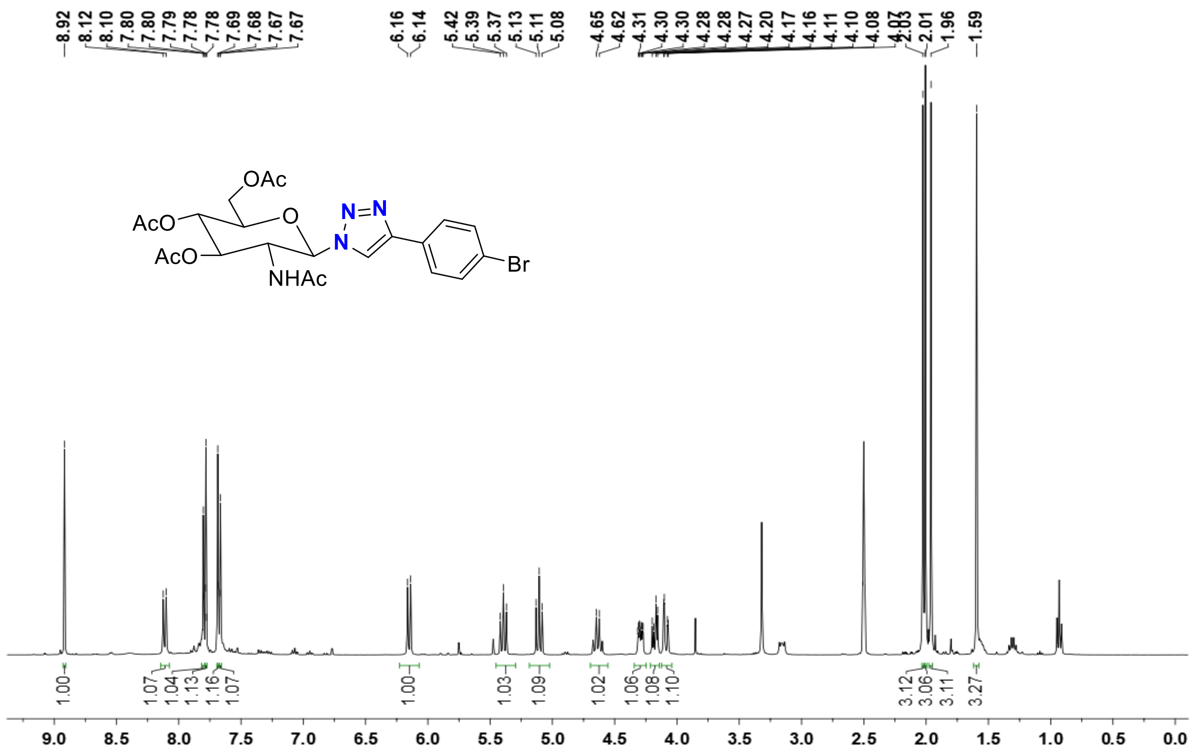

Figure S45. ^{1}H NMR (400 MHz) spectrum of (2R,3S,6S)-5-acetamido-2-(acetoxymethyl)-6-(4-(3-bromophenyl)-1H-1,2,3-triazol-1-yl)tetrahydro-2H-pyran-3,4-diyl diacetate **(D$_3$)** in DMSO at r.t.

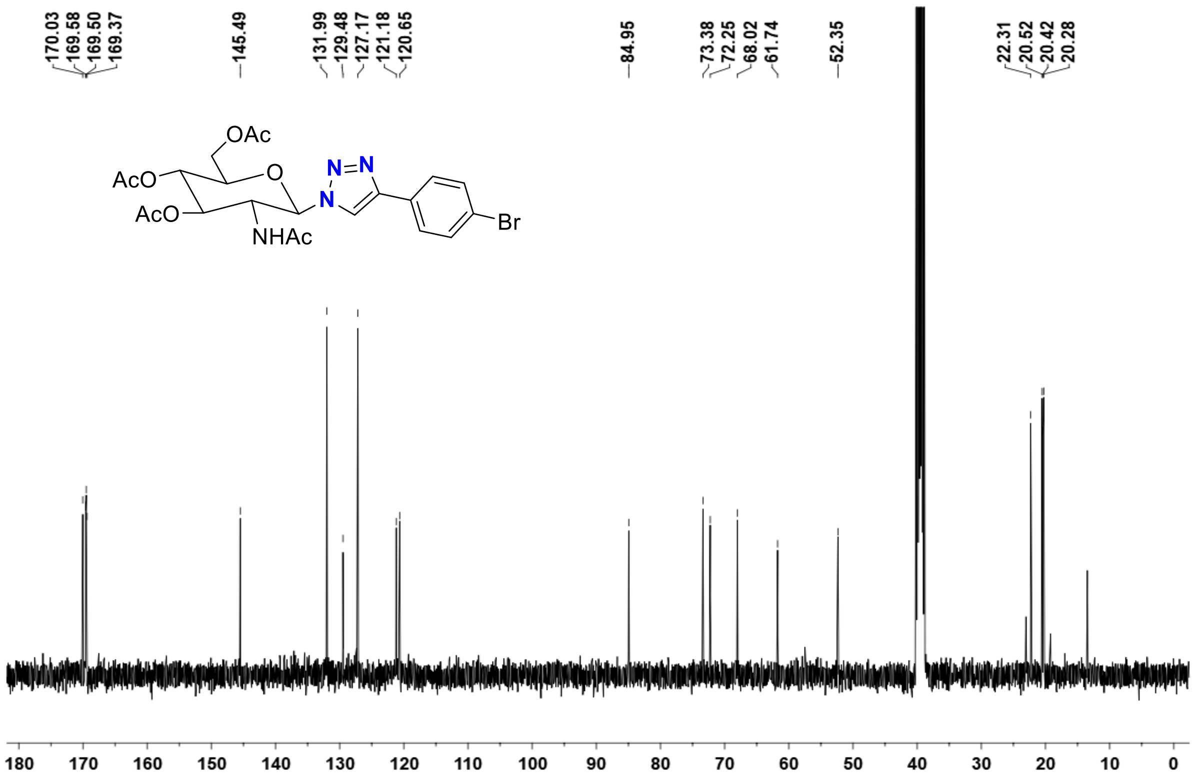

Figure S46. ^{13}C NMR (400 MHz) spectrum of (2R,3S,6S)-5-acetamido-2-(acetoxymethyl)-6-(4-(3-bromophenyl)-1H-1,2,3-triazol-1-yl)tetrahydro-2H-pyran-3,4-diyl diacetate **(D$_3$)** DMSO at r.t.

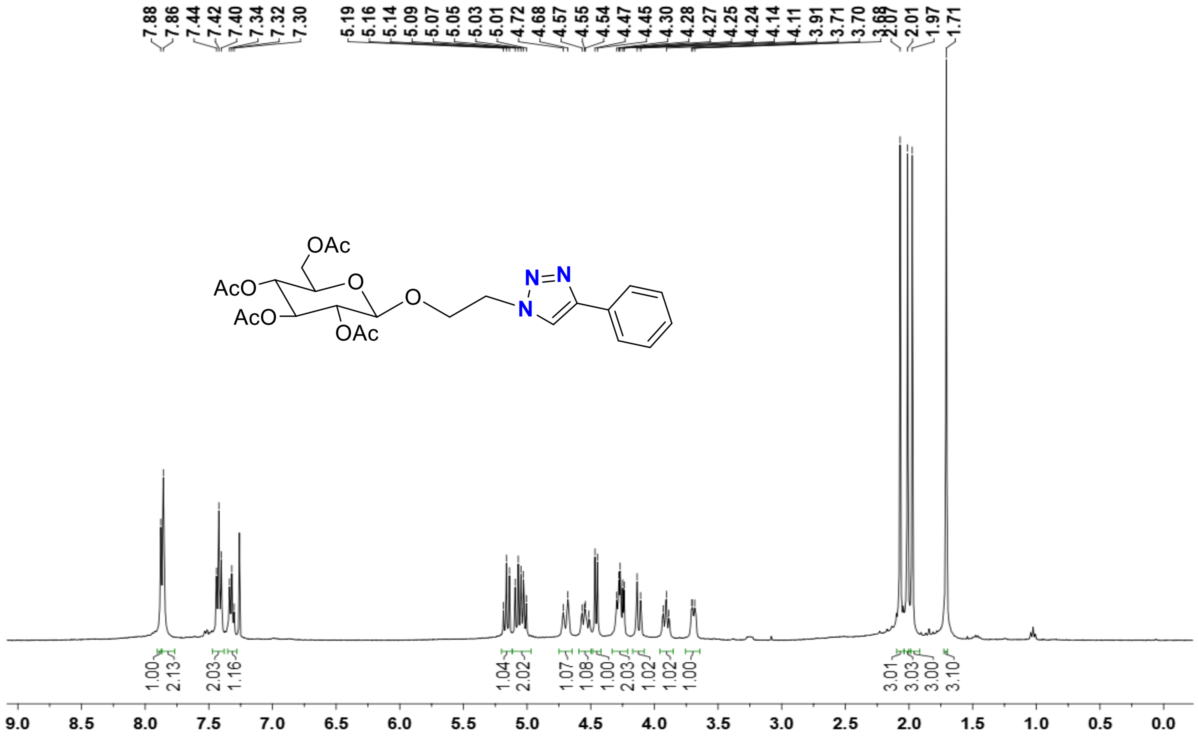

Figure S47. ^{1}H NMR (400 MHz) spectrum of (2R,3R,4S,5R,6R)-2-(acetoxymethyl)-6-(2-(4-phenyl-1H-1,2,3-triazol-1-yl)ethoxy)tetrahydro-2H-pyran-3,4,5-triyl triacetate **(E$_1$)** CDCl$_3$ at r.t.

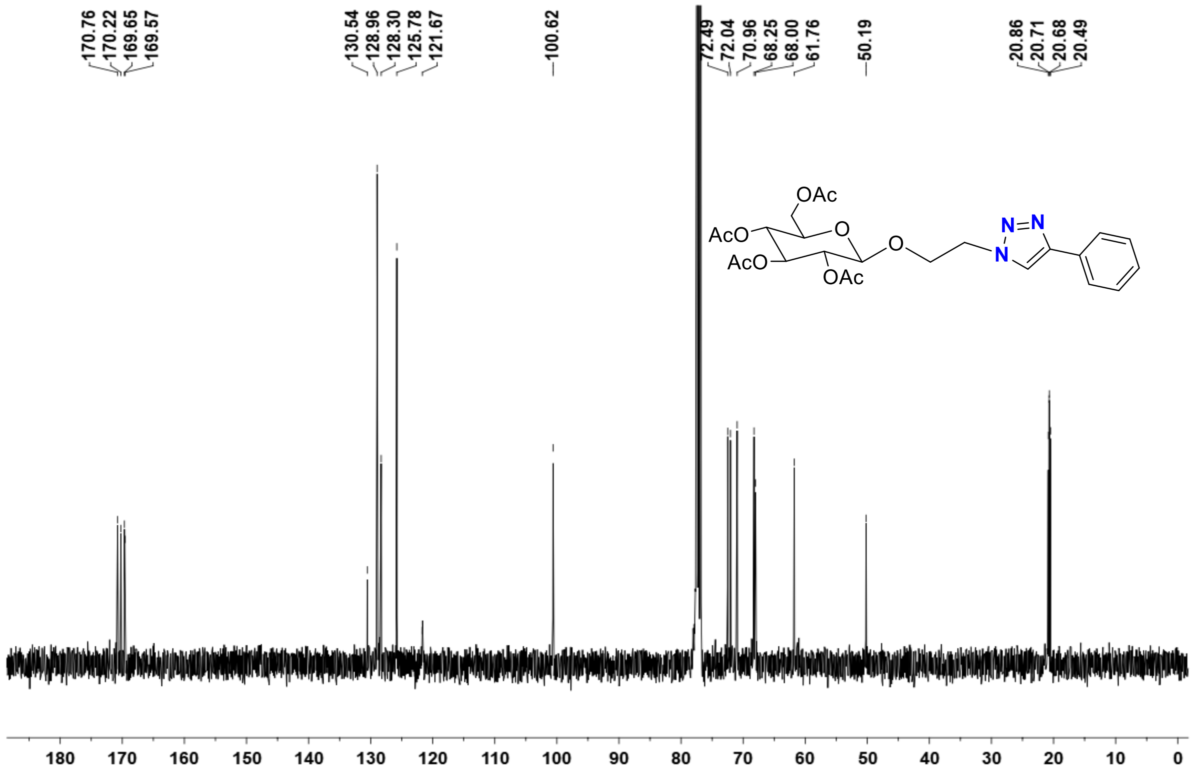

Figure S48. ^{13}C NMR (101 MHz) spectrum of (2R,3R,4S,5R,6R)-2-(acetoxymethyl)-6-(2-(4-phenyl-1H-1,2,3-triazol-1-yl)ethoxy)tetrahydro-2H-pyran-3,4,5-triyl triacetate **(E$_1$)** in CDCl$_3$ at r.t.

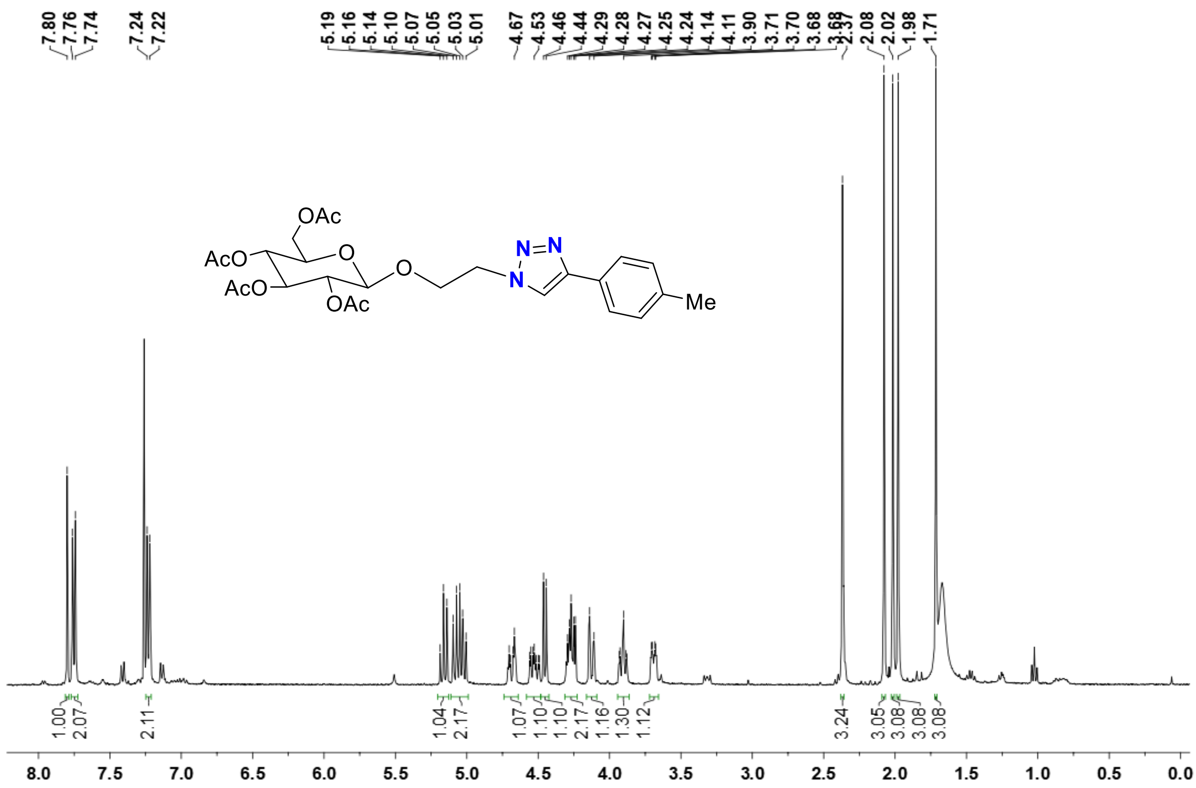

Figure S49. ^{1}H NMR (400 MHz) spectrum of (2R,3R,4S,5R,6R)-2-(acetoxymethyl)-6-(2-(4-(p-tolyl)-1H-1,2,3-triazol-1-yl)ethoxy)tetrahydro-2H-pyran-3,4,5-triyl triacetate **(E$_2$)** $CDCl_3$ at r.t.

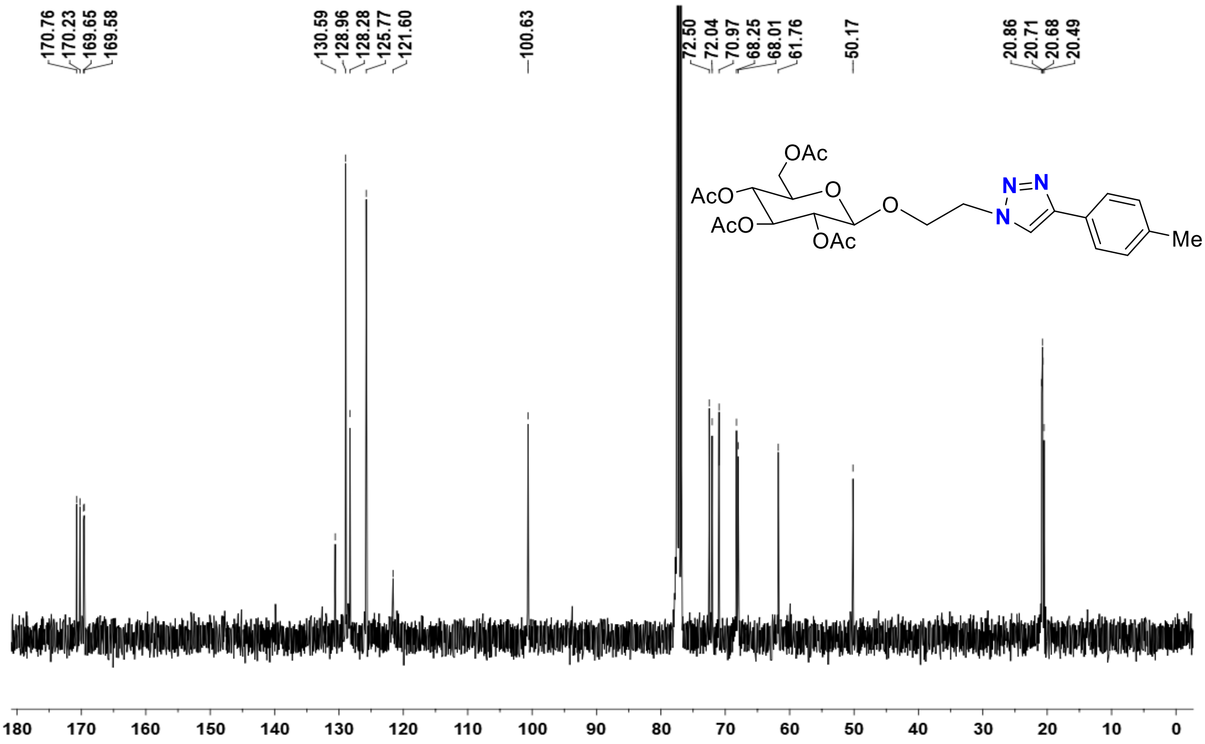

Figure S50. ^{13}C NMR (101 MHz) spectrum of (2R,3R,4S,5R,6R)-2-(acetoxymethyl)-6-(2-(4-(p-tolyl)-1H-1,2,3-triazol-1-yl)ethoxy)tetrahydro-2H-pyran-3,4,5-triyl triacetate **(E$_2$)** in $CDCl_3$ at r.t**.**

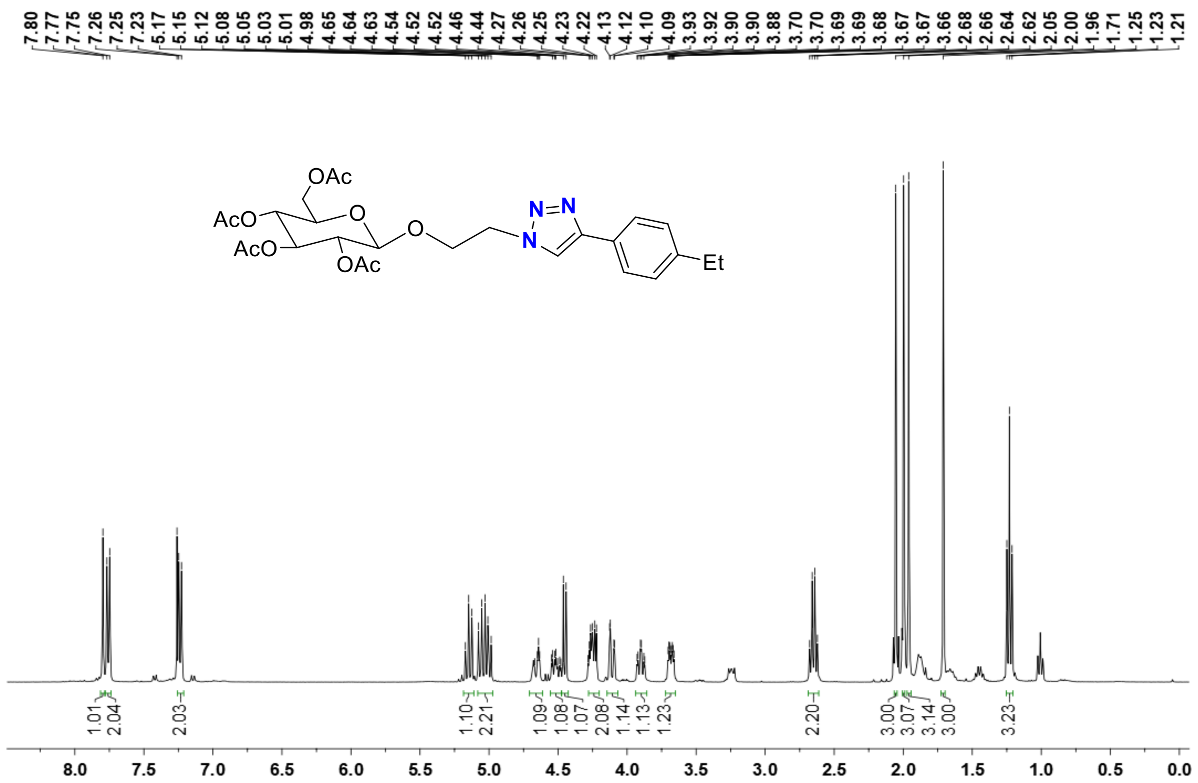

Figure S51. ^{1}H NMR (400 MHz) spectrum of (2R,3R,4S,5R,6R)-2-(acetoxymethyl)-6-(2-(4-(4-ethylphenyl)-1H-1,2,3-triazol-1-yl)ethoxy)tetrahydro-2H-pyran-3,4,5-triyl triacetate **(E3)** $CDCl_3$ at r.t.

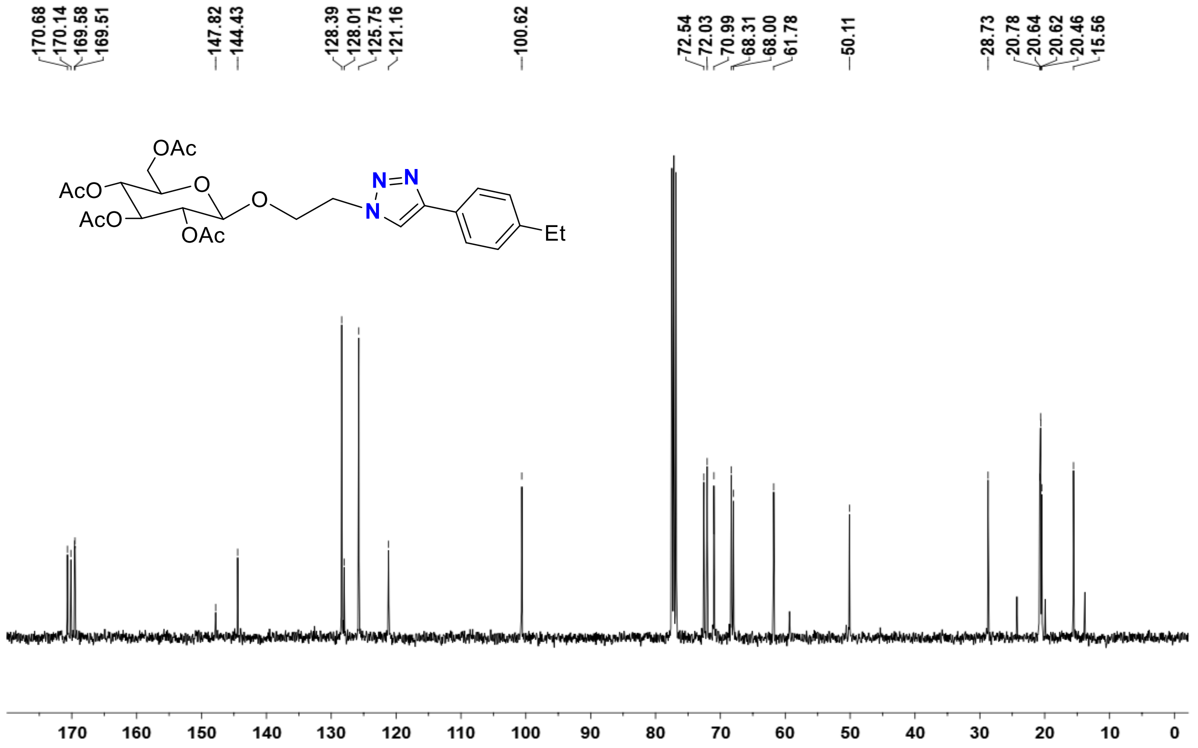

Figure S52. ^{13}C NMR (101 MHz) spectrum of (2R,3R,4S,5R,6R)-2-(acetoxymethyl)-6-(2-(4-(4-ethylphenyl)-1H-1,2,3-triazol-1-yl)ethoxy)tetrahydro-2H-pyran-3,4,5-triyl triacetate **(E3)** in $CDCl_3$ at r.t.

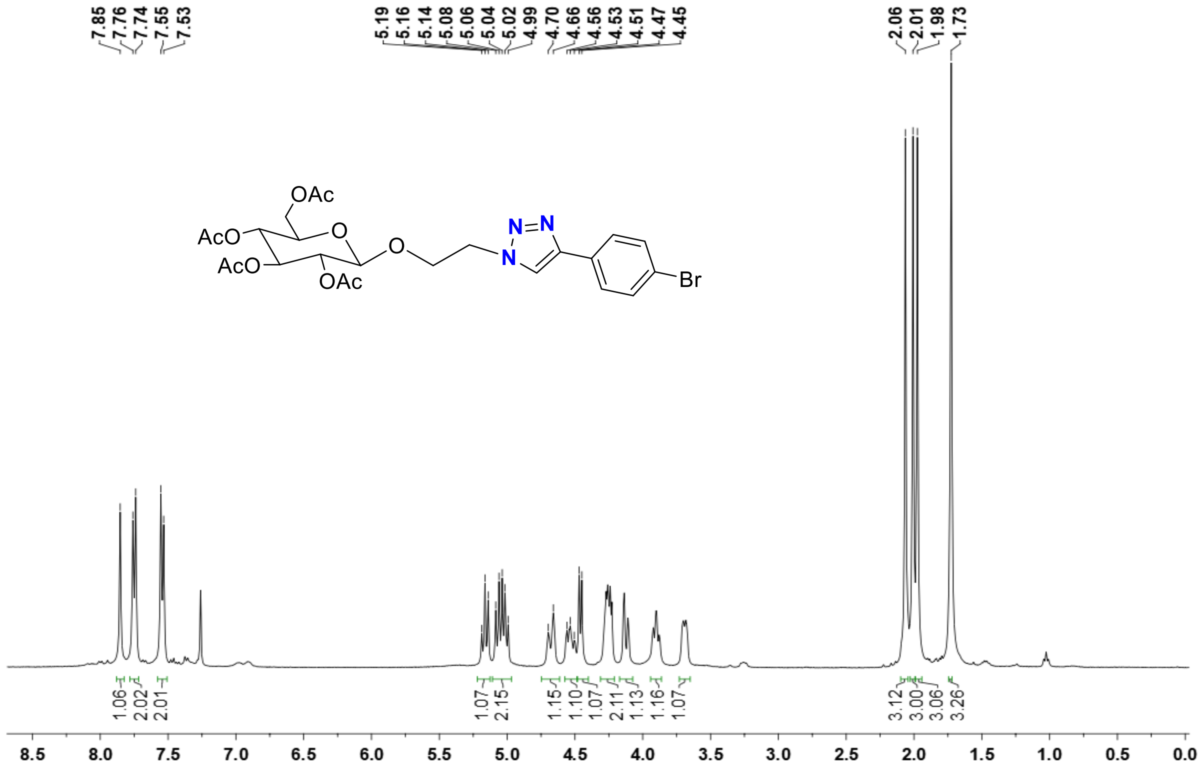

Figure S53. ^{1}H NMR (400 MHz) spectrum of (2R,3R,4S,5R,6R)-2-(acetoxymethyl)-6-(2-(4-(4-bromophenyl)-1H-1,2,3-triazol-1-yl)ethoxy)tetrahydro-2H-pyran-3,4,5-triyl triacetate **(E$_4$)** $CDCl_3$ at r.t.

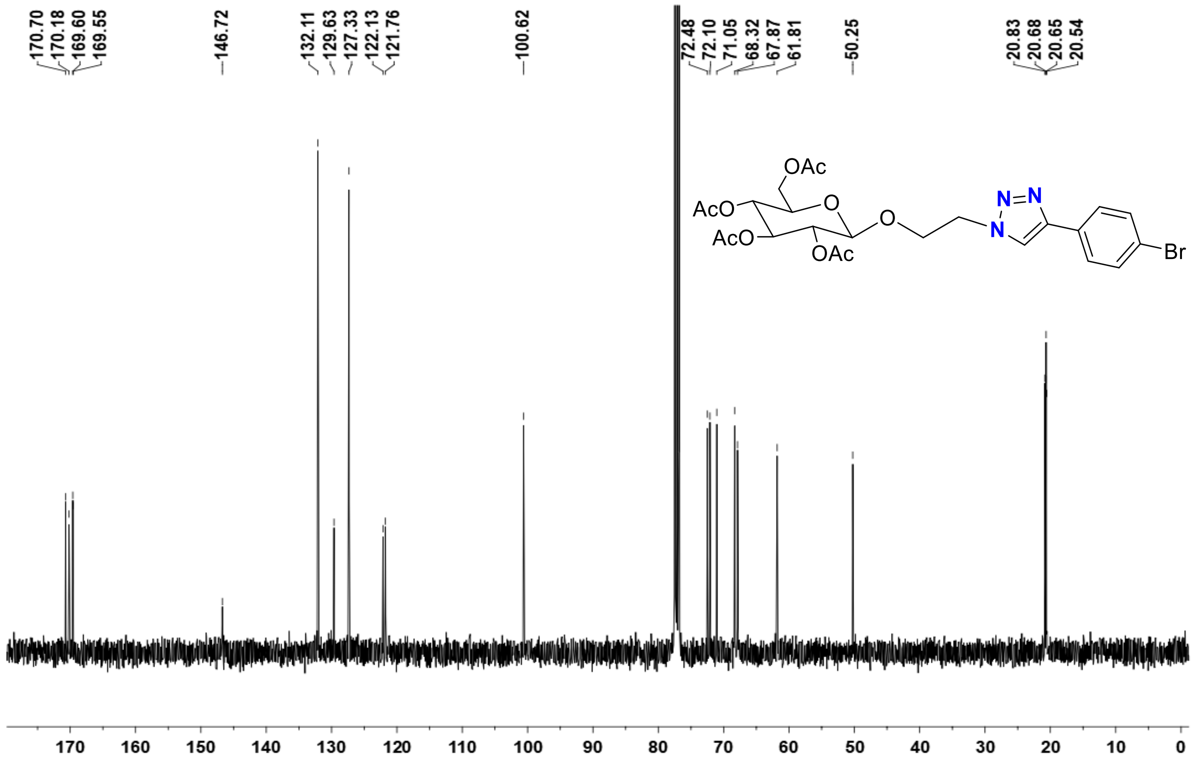

Figure S54. ^{13}C NMR (101 MHz) spectrum of (2R,3R,4S,5R,6R)-2-(acetoxymethyl)-6-(2-(4-(4-bromophenyl)-1H-1,2,3-triazol-1-yl)ethoxy)tetrahydro-2H-pyran-3,4,5-triyl triacetate **(E$_4$)** in $CDCl_3$ at r.t**.**

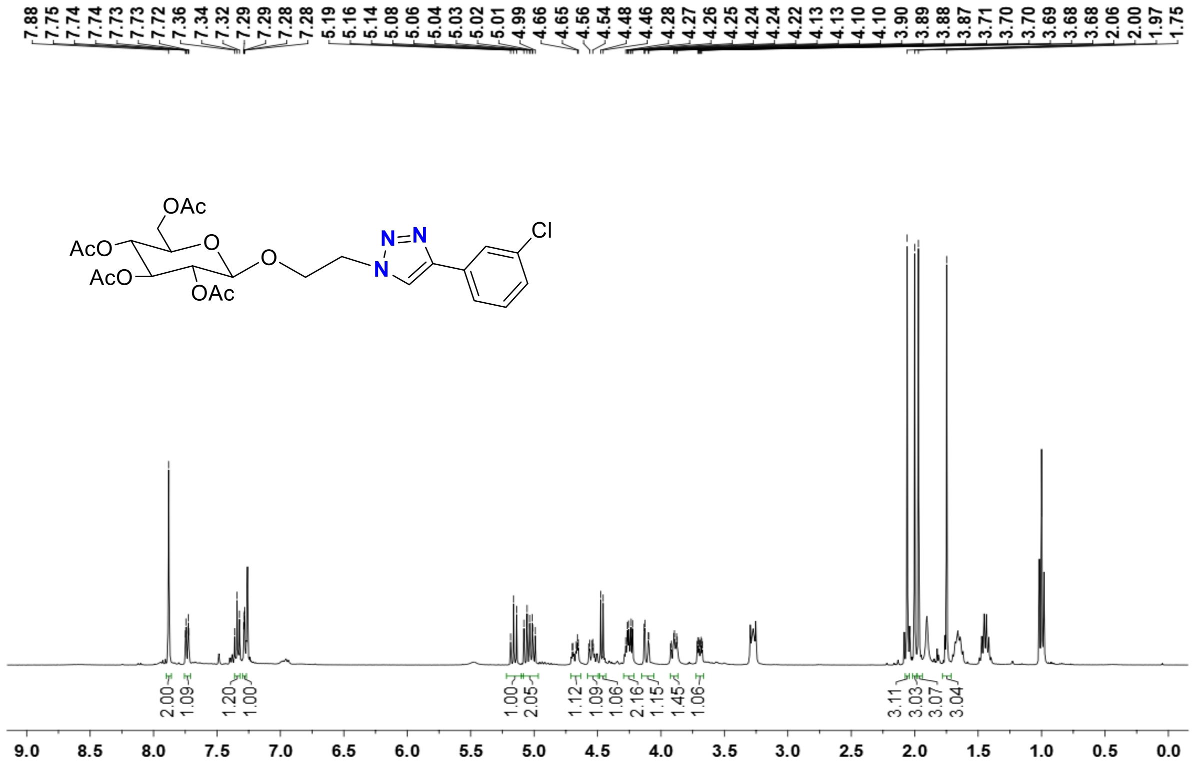

Figure S55. ^{1}H NMR (400 MHz) spectrum of (2R,3R,4S,5R,6R)-2-(acetoxymethyl)-6-(2-(4-(3-chlorophenyl)-1H-1,2,3-triazol-1-yl)ethoxy)tetrahydro-2H-pyran-3,4,5-triyl triacetate **(E$_5$)** $CDCl_3$ at r.t.

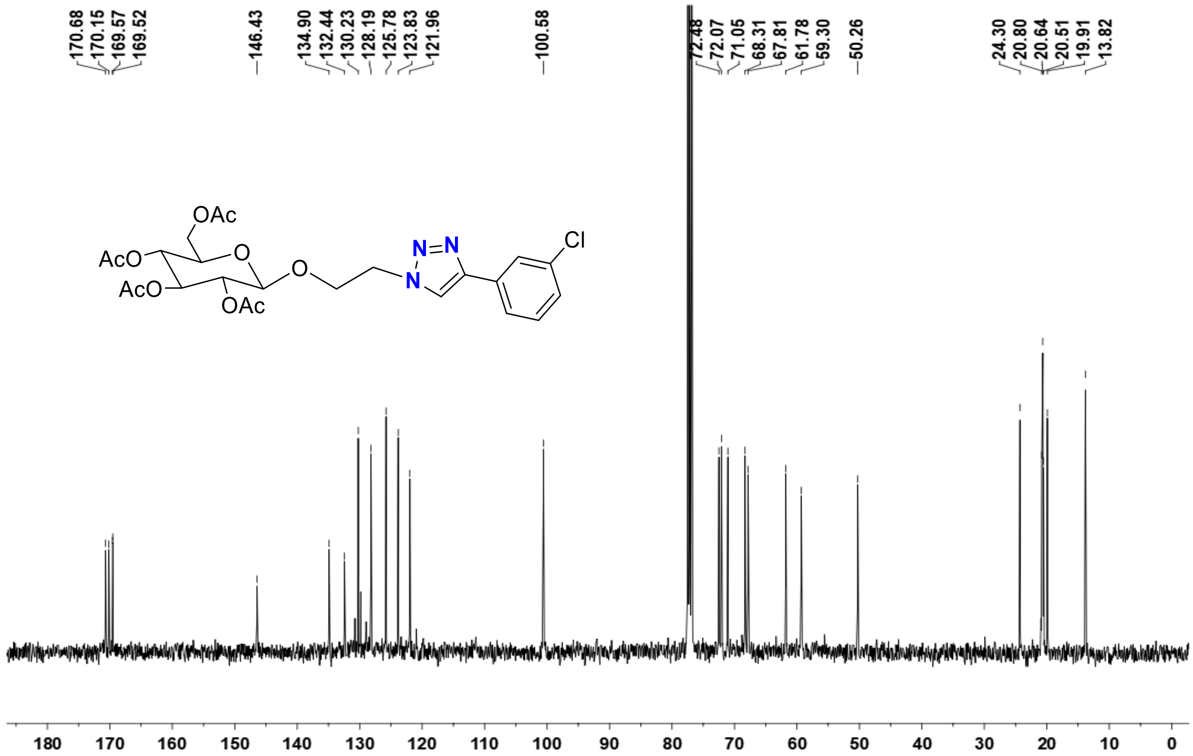

Figure S56. ^{13}C NMR (101 MHz) spectrum (2R,3R,4S,5R,6R)-2-(acetoxymethyl)-6-(2-(4-(3-chlorophenyl)-1H-1,2,3-triazol-1-yl)ethoxy)tetrahydro-2H-pyran-3,4,5-triyl triacetate **(E$_5$)** $CDCl_3$ at r.t.

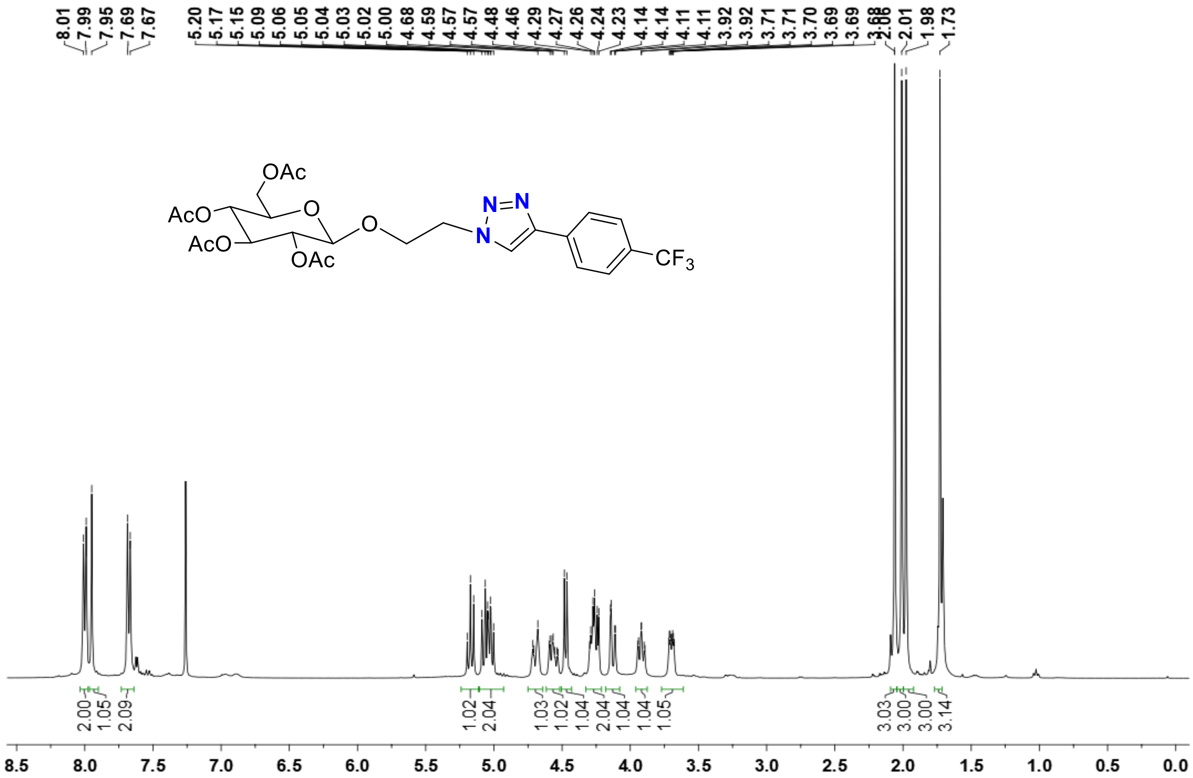

Figure S57. ^{1}H NMR (400 MHz) spectrum of (2R,3R,4S,5R,6R)-2-(acetoxymethyl)-6-(2-(4-(4-(trifluoromethyl)phenyl)-1H-1,2,3-triazol-1-yl)ethoxy)tetrahydro-2H-pyran-3,4,5-triyl triacetate **(E$_6$)** $CDCl_3$ at r.t.

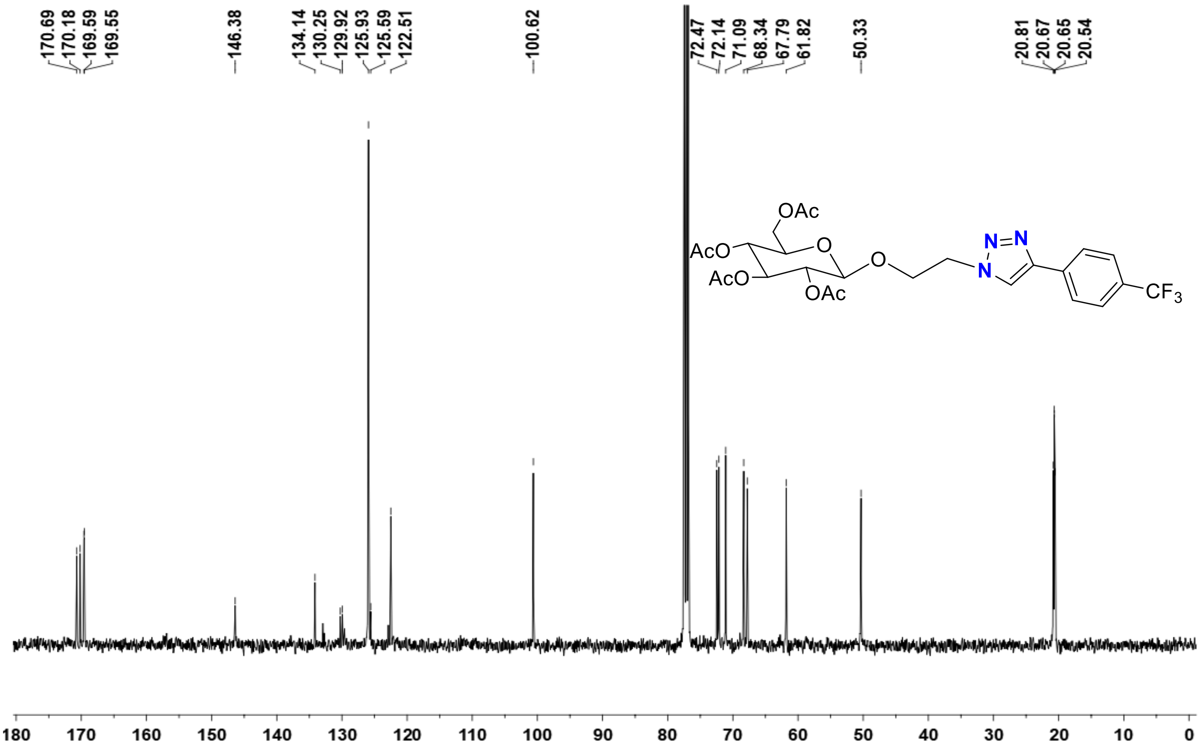

Figure S58. ^{13}C NMR (400 MHz) spectrum of (2R,3R,4S,5R,6R)-2-(acetoxymethyl)-6-(2-(4-(4-(trifluoromethyl)phenyl)-1H-1,2,3-triazol-1-yl)ethoxy)tetrahydro-2H-pyran-3,4,5-triyl triacetate **(E$_6$)** $CDCl_3$ at r.t.

Molecular structure determination by single crystal X-ray crystallography

A crystal of complex **1** with accession code CCDC 2240116 was mounted under crystal oil coated at ambient conditions. All measurements were made on an *Oxford Diffraction SuperNova* area-detector diffractometer using an INCOATEC micro source (Mo-Kα radiation, λ = 0.71073 Å, multilayer optics) and Al filtered.

Data reduction was performed using the *CrysAlisPro*[S4] program. The intensities were corrected for Lorentz and polarization effects, and an absorption correction based on the multi-scan method using SCALE3 ABSPACK in *CrysAlisPro*[S4] was applied. Data collection and refinement parameters are given in Table S1.

OleX[S5] and refinement was carried out using least-square minimization implemented in ShelXL.[S6, S7] All nonhydrogen atoms were refined with anisotropic displacement parameters. Hydrogen atom positions were fixed geometrically in idealized positions and were refined using a riding model.

Table S1. Crystallographic Data and Refinement Parameters for **1**.

Complex	**1**
Empirical formula	$C_{16}H_{15}CuIN_3O$
Formula weight (g mol^{-1})	455.75
Temperature	100.00(10)
Radiation	MoKα (λ = 0.71073)
Crystal system	Monoclinic
Space group	$P2_1/c$
a (Å)	12.2982(5)
b (Å)	8.7867(3)
c (Å)	15.5899(6)
α (deg)	90
β (deg)	109.228(4)
γ (deg)	90
volume (Å^3)	1590.68(11)
Z	4
D_{calc} (g cm^{-3})	1.381
μ (mm^{-1})	1.903
F(000)	888.0
Crystal Size (mm^3)	0.2 × 0.1 × 0.1
2θ Range (deg)	6.92 to 60.878
Index Ranges	$-15 \leq h \leq 16$, $-11 \leq k \leq 11$, $-21 \leq l \leq 19$
Reflections collected	15069
Independent reflections	3809 [R_{int} = 0.0476, R_{sigma} = 0.0397]
Completeness to theta	99.96
Refinement method	Full-matrix least-squares on F^2
Data/Restraints/parameters	3809/0/200
Goodness-of-fit on *F*2	1.038
Final *R* indices [I>2σ(I)]	R_1 = 0.0271, wR_2= 0.0623
R indices (all data)	R_1 = 0.0322, wR_2= 0.0644
Largest diff. peak/hole (e Å^{-3})	0.69/-0.69

Table S2. Selected bond lengths (Å) around the metal centre in copper complexes **1** and **2**.

	Bond lengths
Complexes/Bonds	**1**
Cu−N(pyridine)	2.091(2)
Cu−N(pyrazole)	2.118(2)
Cu−I<br>Cu^1−I	2.6250(4)<br>2.6271(3)
Cu−Cu^a	2.6101(6)

Symmetry code: ^{a}1-X,1-Y,1-Z

Table S3. Selected bond angles (°) around the metal centre in copper complexes **1** and **2**.

Bond angles	
Cu1–I1–$Cu1^a$	59.599(11)
I1–Cu1–$I1^a$	120.401(11)
$Cu1^a$–Cu1–I1	60.240(12)
$Cu1^a$–Cu1–$I1^a$	60.161(12)
N3–Cu1–$I1^a$	104.02(6)
N3–Cu1–I1	119.32(6)
N3–Cu1–$Cu1^a$	137.42(6)
N1–Cu1–I1	103.51(6)
N1–Cu1–$I1^a$	112.20(6)
N1–Cu1–$Cu1^a$	127.96(6)
N1–Cu1–N3	94.44(8)

Symmetry code: ^{a} 1-X,1-Y,1-Z

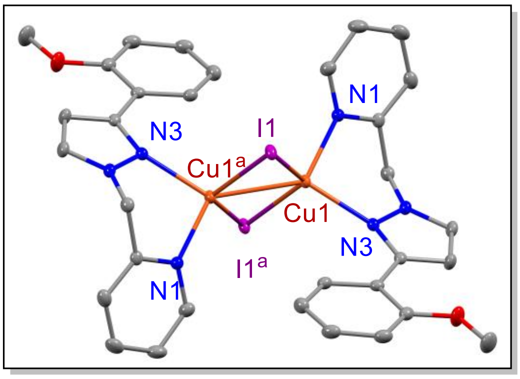

Figure S59. Molecular structure of complex **1**.

Table S4. Optimised Reaction (Method A: DES TBAB/glycerol, [Cu] 3 mol%, 70 °C, 12 h): Zero Pass CHEM21 green metrics toolkit

Supp+J5+B1:S29+B1:S32+B1:S31+B1:S30+B1:S29+B+B1: Summary of Zero Pass Metrics Toolkit

Yield, conversion, selectivity, AE, RME

Reactant (Limiting Reactant First)	Mass (g)	MW	Mol	Catalyst	Mass (g)	Reagent	Mass (g)	Reaction solvent	Volume (cm^3)	Density (g ml^{-1})	Mass (g)	Work up chemical	Mass (g)	Workup solvent	Volume (cm3)	Density (g ml^{-1})	Mass (g)
Glycosides	4.11	411.20	0.01	[Cu]	0.14			TBAB/glycerol	40.00	1.18	47.27			25 % ammonia solut	20.00	0.90	18.00
Phenylacetylene	1.02	102.13	0.01														
Sodium azide	1.63	65.01	0.02														
Total	6.76	578.34			0.14		0.00				47.27		0.00				18.00

$$AE = \frac{molecular\ weight\ of\ product}{total\ molecualr\ weight\ of\ reactants} \times 100$$

$$RME = \frac{mass\ of\ isolated\ product}{total\ mass\ of\ reactants} \times 100$$

		Flag	
Yield	98.0	●	98.0
Conversion	100.0	●	100.0
Selectivity	98.0	●	98.0
AE	82.2		
RME	69.0		

	mass	mw	mol
Product	4.659	475.454	0.0097991

	mass
Unreacted limiting reactant	0.000

Solvents (Zero Pass)

Highly hazardous solvents (Red flag for any of the following)	List Highly Hazardous Solvents Below
Et_2O, Benzene, CCl_4, chloroform, DCE, nitromethane, CS_2, HMPA	None

Health and Safety (Zero Pass)

Health & safety (Red flag for any of the following)		List substances plus the red flagged H-codes below
Highly explosive	H200, H201, H202, H203	None
Explosive thermal runaway	H240	None
Fatally toxic	H300, H310, H330	None
Mutagenic	H350	None
Repro-toxic	H360	None
Serious environmental implications	H420	None

Table S5. Optimised Reaction (Method A: DES TBAB/glycerol, [Cu] 3 mol%, 70 °C, 12 h): First Pass CHEM21 green metrics toolkit

Supplementary Information: Appendix 2 — **Summary of First Pass Metrics Toolkit**

Yield, AE, RME, MI/PMI and OE

Reactant (Limiting Reactant First)	Mass (g)	MW	Mol	Catalyst	Mass (g)	Reagent	Mass (g)	Reaction solvent	Volume (cm^3)	Density (g ml^{-1})	Mass (g)	Work up chemical	Mass (g)	Workup solvent	Volume (cm3)	Density (g ml^{-1})	Mass (g)
Glycosides	4.11	411.20	0.01	[Cu]	0.14			TBAB/glycerol	40.00	1.18	47.27			25 % ammonia solu	20.00	0.90	18.00
Phenylacetylene	1.02	102.13	0.01														
Sodium azide	1.63	65.01	0.02														
Total	6.76	578.34			0.14		0.00				47.27		0.00				18.00

Metric	Value	Flag	
Yield	98.0	●	98.0
Conversion	100.0	●	100.0
Selectivity	98.0	●	98.0
AE	82.2		
RME	69.0	OE	83.9
PMI total	15.5		
PMI Reaction	11.6		
reagents, catlyst	1.5		
PMI reaction solvents	10.1		
PMI Workup	3.9		
PMI Workup chemical	0.0		
PMI workup solvents	3.9		

	Mass	MW	Mol
Product	4.659	475.454	0.01
	mass		
Unreacted limiting reactant	0.00		

$$RME = \frac{\textit{mass of isolated product}}{\textit{total mass of reactants}} \times 100$$

$$AE = \frac{\textit{molecular weight of product}}{\textit{total molecualr weight of reactants}} \times 100$$

$$\textit{mass intensity} = \frac{\textit{total mass in a process or process step}}{\textit{mass of product}}$$

$$OE = \frac{RME}{AE} \times 100$$

Solvents (First Pass)

		List solvents below
**Preferred solvents**	**water,** EtOH, nBuOH, AcOipr, AcOnBu, PhOMe, MeOH, tBuOH, BnOH, ethylene glycol, acetone, MEK, MIBK, **AcOEt,** sulfolane	TBAB: Glycerol (1: 4)
**Problematic solvents:** (acceptable only if substitution does not offer advantages)	DMSO, cyclohexanone, DMPU, AcOH, Ac2O, Acetonitrile, AcOMe, THF, heptane, Me-cyclohexane, toluene, xylene, MTBE, **cyclohexane**, chlorobenzene, formic acid, pyridine, Me-THF	none
**Hazardous solvents**: These solvents have significant health and/or safety concerns.	dioxane, pentane, TEA, diisopropyl ether, DME, DCM, DMF, DMA, NMP, methoxyethanol, hexane	none
**Highly hazardous solvents:** The solvents which are agreed not to be used, even in screening	Et_2O, Benzene, CCl_4, chloroform, DCE, nitromethane, CS_2, HMPA	none

Catalyst/enzyme (First Pass)

		Tick
Catalyst or enzyme used, **or** reaction takes place without **any** catalyst/reagents.	Green Flag	X
Use of stoichiometric quantities of reagents	Amber Flag	
Use of reagents in excess	Red Flag	

		Tick
Facile recovery of catalyst/enzyme	Green Flag	
catalyst/enzyme not recovered	Amber Flag	X

Critical elements

Supply remaining	Flag colour	Note element
5-50 years	Red Flag	
50-500 years	Amber Flag	Cu
+500 years	Green Flag	

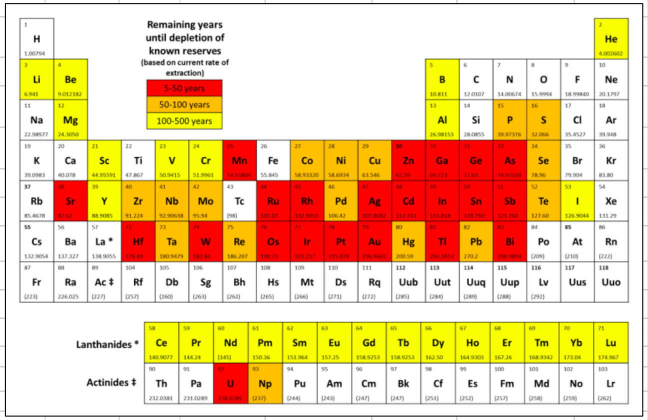

Energy (First Pass)

		Tick
Reaction run between 0 to 70°C	Green Flag	X
Reaction run between -20 to 0 or 70 to 140°C	Amber Flag	
Reaction run below -20 or above 140°C	Red Flag	

		Tick
Reaction run at reflux	Red Flag	
Reaction run 5°C or more below the solvent boiling point	Green Flag	X

Batch/flow

		Tick
Flow	Green Flag	
Batch	Amber Flag	X

Work Up

		List
quenching<br>filtration<br>centrifugation<br>crystallisation<br>Low temperature distillation/evaporation/ sublimation	Green Flag	Filtration
solvent exchange, quenching into aqueous solvent	Amber Flag	
chromatography/ion exchange<br>high temperature<br>multiple recrystallisation	Red Flag	

Health & safety

	Red Flag	Amber Flag	Green Flag
Highly explosive	H200, H201, H202, H203	H205, H220, H224	If no red or amber flagged H codes present then green flag
Explosive thermal runaway	H230, H240, H250	H241	
Toxic	H300, H310, H330	H301, H311, H331,	
Long Term toxicity	H340, H350, H360, H370, H372	H341, H351, H361, H371, H373	
Environmental implications	H400, H410, H411, H420	H401, H412	

List substances and H-codes (Red)	List substances and H-codes (Amber)	List substances and H-codes (Green)
**Phenylacetylene:** H350; **Sodium azide**: H300, H310, H330, H410. **Ammonia solution:** H370.	**Sodium azide:** H373; **Ammonia solution**: H301, H311, H331.	**Acetobromo-α-D-glucose:** H315, H319, H335, **Phenylacetylene**: H226, H315, H319, H335. H350. **Sodium azide:** H300, H310, H330, H373, H410; **TBAB:** H302, H315, H319, H335. **Glycerol**: H319. **Ammonia solution:** H221, H225, H314

Use of chemicals of environmental concern

		List substances of very high concern
Chemical identified as Substances of Very High Concern by ChemSec which are utilised	Red Flag	None

Table S6. Optimised Reaction (Method B: DES ChCl/glycerol, [Cu] 3 mol%, 70 °C, 12 h): Zero Pass CHEM21 green metrics toolkit

Supp+J5+B1:S29+B1:S32+B1:S31+B1:S30+B1:S29+B+B1: Summary of Zero Pass Metrics Toolkit

Yield, conversion, selectivity, AE, RME

Reactant (Limiting Reactant First)	Mass (g)	MW	Mol	Catalyst	Mass (g)	Reagent	Mass (g)	Reaction solvent	Volume (cm^3)	Density (g ml^{-1})	Mass (g)	Work up chemical	Mass (g)	Workup solvent	Volume (cm3)	Density (g ml^{-1})	Mass (g)
Glycosides	4.11	411.20	0.01	[Cu]	0.14			ChCl/glycerol	40.00	1.19	47.76			25 % ammonia solut	20.00	0.90	18.00
Phenylacetylene	1.02	102.13	0.01														
Sodium azide	1.63	65.01	0.02														
Total	6.76	578.34			0.14		0.00				47.76		0.00				18.00

$$AE = \frac{molecular\ weight\ of\ product}{total\ molecualr\ weight\ of\ reactants} \times 100$$

$$RME = \frac{mass\ of\ isolated\ product}{total\ mass\ of\ reactants} \times 100$$

Metric	Value	Flag
Yield	94.0	94.0
Conversion	100.0	100.0
Selectivity	94.0	94.0
AE	82.2	
RME	66.1	

	mass	mw	mol
Product	4.469	475.454	0.0093994
	mass		
Unreacted limiting reactant	0.000		

Solvents (Zero Pass)

Highly hazardous solvents (Red flag for any of the following)	List Highly Hazardous Solvents Below
Et_2O, Benzene, CCl_4, chloroform, DCE, nitromethane, CS_2, HMPA	None

Health and Safety (Zero Pass)

Health & safety (Red flag for any of the following)		List substances plus the red flagged H-codes below
Highly explosive	H200, H201, H202, H203	None
Explosive thermal runaway	H240	None
Fatally toxic	H300, H310, H330	None
Mutagenic	H350	None
Repro-toxic	H360	None
Serious environmental implications	H420	None

Table S7. Optimised Reaction (Method B: DES ChCl/glycerol, [Cu] 3 mol%, 70 °C, 12 h): First Pass CHEM21 green metrics toolkit

Supplementary Information: Appendix 2 | **Summary of First Pass Metrics Toolkit**

Yield, AE, RME, MI/PMI and OE

Reactant (Limiting Reactant First)	Mass (g)	MW	Mol	Catalyst	Mass (g)	Reagent	Mass (g)	Reaction solvent	Volume (cm^3)	Density (g ml^{-1})	Mass (g)	Work up chemical	Mass (g)	Workup solvent	Volume (cm3)	Density (g ml^{-1})	Mass (g)
Glycosides	4.11	411.20	0.01	[Cu]	0.14			ChCl/glycerol	40.00	1.19	47.76			25 % ammonia solu	20.00	0.90	18.00
Phenylacetylene	1.02	102.13	0.01														
Sodium azide	1.63	65.01	0.02														
Total	6.76	578.34			0.14		0.00				47.76		0.00				18.00

$$RME = \frac{mass\ of\ isolated\ product}{total\ mass\ of\ reactants} \times 100$$

$$AE = \frac{molecular\ weight\ of\ product}{total\ moleculr\ weight\ of\ reactants} \times 100$$

$$mass\ intensity = \frac{total\ mass\ in\ a\ process\ or\ process\ step}{mass\ of\ product}$$

$$OE = \frac{RME}{AE} \times 100$$

Metric	Value	Flag	
Yield	94.0	●	94.0
Conversion	100.0	●	100.0
Selectivity	94.0	●	94.0
AE	82.2		
RME	66.1	OE	80.5
PMI total	16.3		
PMI Reaction	12.2		
reagents, catlyst	1.5		
PMI reaction solvents	10.7		
PMI Workup	4.0		
PMI Workup chemical	0.0		
PMI workup solvents	4.0		

	Mass	MW	Mol
Product	4.469	475.454	0.01
	mass		
Unreacted limiting reactant	0.00		

Solvents (First Pass)

		List solvents below
**Preferred solvents**	**water,** EtOH, nBuOH, AcOipr, AcOnBu, PhOMe, MeOH, tBuOH, BnOH, ethylene glycol, acetone, MEK, MIBK, **AcOEt,** sulfolane	TBAB: Glycerol (1: 4)
**Problematic solvents:** (acceptable only if substitution does not offer advantages)	DMSO, cyclohexanone, DMPU, AcOH, Ac2O, Acetonitrile, AcOMe, THF, heptane, Me-cyclohexane, toluene, xylene, MTBE, **cyclohexane**, chlorobenzene, formic acid, pyridine, Me-THF	none
**Hazardous solvents**: These solvents have significant health and/or safety concerns.	dioxane, pentane, TEA, diisopropyl ether, DME, DCM, DMF, DMA, NMP, methoxyethanol, hexane	none
**Highly hazardous solvents:** The solvents which are agreed not to be used, even in screening	Et_2O, Benzene, CCl_4, chloroform, DCE, nitromethane, CS_2, HMPA	none

Critical elements

Supply remaining	Flag colour	Note element
5-50 years	Red Flag	
50-500 years	Amber Flag	Cu
+500 years	Green Flag	

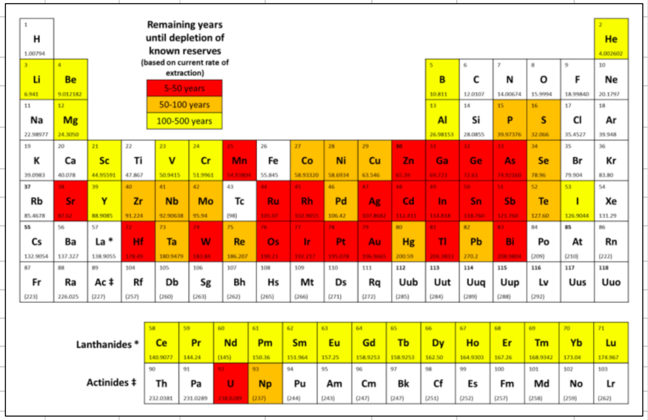

Energy (First Pass)

		Tick
Reaction run between 0 to 70°C	Green Flag	X
Reaction run between -20 to 0 or 70 to 140°C	Amber Flag	
Reaction run below -20 or above 140°C	Red Flag	

		Tick
Reaction run at reflux	Red Flag	
Reaction run 5°C or more below the solvent boiling point	Green Flag	X

Batch/flow

		Tick
Flow	Green Flag	
Batch	Amber Flag	X

Work Up

		List
quenching<br>filtration<br>centrifugation<br>crystallisation<br>Low temperature distillation/evaporation/ sublimation	Green Flag	Filtration
solvent exchange, quenching into aqueous solvent	Amber Flag	
chromatography/ion exchange<br>high temperature<br>multiple recrystallisation	Red Flag	

Health & safety

	Red Flag	Amber Flag	Green Flag
Highly explosive	H200, H201, H202, H203	H205, H220, H224	If no red or amber flagged H codes present then green flag
Explosive thermal runaway	H230, H240, H250	H241	
Toxic	H300, H310, H330	H301, H311, H331,	
Long Term toxicity	H340, H350, H360, H370, H372	H341, H351, H361, H371, H373	
Environmental implications	H400, H410, H411, H420	H401, H412	

List substances and H-codes (Red)	List substances and H-codes (Amber)	List substances and H-codes (Green)
**Phenylacetylene:** H350; **Sodium azide**: H300, H310, H330, H410. **Ammonia solution:** H370.	**Sodium azide:** H373; **Ammonia solution**: H301, H311, H331.	**Acetobromo-α-D-glucose:** H315, H319, H335, **Phenylacetylene**: H226, H315, H319, H335. H350. **Sodium azide:** H300, H310, H330, H373, H410; **Choline Chloride:** H315, H319, H335. H303 **Glycerol**: H319. **Ammonia solution:** H221, H225, H314

Use of chemicals of environmental concern

		List substances of very high concern
Chemical identified as Substances of Very High Concern by ChemSec which are utilised	Red Flag	None

References

S1. R. Ghosh, R. R. Behera, S. Panda, S. K. Behera, N. C. Jana and B. Bagh, *ChemCatChem*, 2022, e202201062.

S2. A. Kushwaha, A. K. Agrihari, K. Manar, C. Yadav, V. K. Tiwari, M. G. B. Drew and N. Singh, *New J. Chem*., 2019, **43**, 8939–8949.

S3. S. Lal and S. Díez-González, *J. Org. Chem*., 2011, **76**, 2367–2373.

S4. Oxford Diffraction (2010). *CrysAlisPro* (Version 1.171.34.44). Oxford Diffraction Ltd., Yarnton, Oxfordshire, UK.

S5. O. V. Dolomanov, L. J. Bourhis, R. J. Gildea. Howard, J. A. K.; Puschmann, H. OLEX2: a complete structure solution, refinement and analysis program. *J. Appl. Crystallogr*., 2009, **42**, 339−341.

S6. G. M. Sheldrick, *Acta Cryst*., 2015, **A71**, 3–8.

S7. G. M. Sheldrick, *Acta Cryst*., 2015, **C71**, 3–8.
